# Measuring team factors thought to influence the success of quality improvement in primary care: a systematic review of instruments

**DOI:** 10.1186/1748-5908-8-20

**Published:** 2013-02-14

**Authors:** Sue E Brennan, Marije Bosch, Heather Buchan, Sally E Green

**Affiliations:** 1 School of Public Health and Preventive Medicine, Monash University, Melbourne, Australia; 2 Central Clinical School, Monash University and National Trauma Research Institute, Melbourne, Australia; 3 Australian Commission on Safety and Quality in Health Care (ACSQHC), Sydney, Australia

**Keywords:** Continuous quality improvement, Primary care, Evaluation, Systematic review, Measurement, Instrument, Conceptual framework, Taxonomy, Teamwork, Team functioning

## Abstract

**Background:**

Measuring team factors in evaluations of Continuous Quality Improvement (CQI) may provide important information for enhancing CQI processes and outcomes; however, the large number of potentially relevant factors and associated measurement instruments makes inclusion of such measures challenging. This review aims to provide guidance on the selection of instruments for measuring team-level factors by systematically collating, categorizing, and reviewing quantitative self-report instruments.

**Methods:**

Data sources: We searched MEDLINE, PsycINFO, and Health and Psychosocial Instruments; reference lists of systematic reviews; and citations and references of the main report of instruments. Study selection: To determine the scope of the review, we developed and used a conceptual framework designed to capture factors relevant to evaluating CQI in primary care (the InQuIRe framework). We included papers reporting development or use of an instrument measuring factors relevant to teamwork. Data extracted included instrument purpose; theoretical basis, constructs measured and definitions; development methods and assessment of measurement properties. Analysis and synthesis: We used qualitative analysis of instrument content and our initial framework to develop a taxonomy for summarizing and comparing instruments. Instrument content was categorized using the taxonomy, illustrating coverage of the InQuIRe framework. Methods of development and evidence of measurement properties were reviewed for instruments with potential for use in primary care.

**Results:**

We identified 192 potentially relevant instruments, 170 of which were analyzed to develop the taxonomy. Eighty-one instruments measured constructs relevant to CQI teams in primary care, with content covering teamwork context (45 instruments measured enabling conditions or attitudes to teamwork), team process (57 instruments measured teamwork behaviors), and team outcomes (59 instruments measured perceptions of the team or its effectiveness). Forty instruments were included for full review, many with a strong theoretical basis. Evidence supporting measurement properties was limited.

**Conclusions:**

Existing instruments cover many of the factors hypothesized to contribute to QI success. With further testing, use of these instruments measuring team factors in evaluations could aid our understanding of the influence of teamwork on CQI outcomes. Greater consistency in the factors measured and choice of measurement instruments is required to enable synthesis of findings for informing policy and practice.

## Background

The use of cross-functional teams to diagnose process-based quality problems, and develop and test process improvements, is an important element of continuous quality improvement (CQI) [1-3]. Cross-functional teamwork aims to capitalize on the varied knowledge and perspectives of team members, encouraging collaboration that is expected to lead to better problem solving, more innovative decisions, and greater engagement in implementing proposed solutions [4-6]. Cross-functional teams may also promote organizational learning [7]. In CQI, the use of cross-functional teams is expected to lead to better processes of care and greater adherence to the new processes [2,3,8]. Realizing these benefits requires teams with both task-specific competencies (knowledge and skills required to use CQI methods) and teamwork competencies (knowledge and skills that enable members to function as an effective team). It also requires a context that enables teams to overcome well-documented barriers to cross-functional teamwork, such as professional boundaries and status differences that hinder collaboration [4,6,9-11].

Despite the centrality of teamwork to CQI, the extent to which team functioning influences CQI outcomes is not well understood [12-14]. In a systematic review of contextual factors thought to influence QI success, thirteen studies examined team-level factors [14]. Team leadership, team climate, team process, and physician involvement in the QI team appeared to be important, but the supporting evidence was scant [14]. Research on QI collaboratives—an approach in which teams use CQI methods to introduce change—has led to a greater focus on the influence of teams on QI outcomes (*e.g.,*[15-20]). Yet in a recent review of the impact of QI and safety teams [13], the authors concluded that existing studies provided limited information about the attributes of successful QI teams or factors that influence team success.

One of the challenges to addressing the limited evidence base is the variability in how team-level factors are conceptualized and measured in QI studies [14]. This variability fragments the evidence base, making it difficult to compare and synthesize findings across studies. Recent calls to address these challenges focus on the need for theory development to explain how CQI works and factors that influence its effectiveness, and the identification of valid and reliable measures to enable theories to be tested [21-26].

In this paper, we report a systematic review of instruments measuring team-level factors thought to influence the success of CQI. This review is part of a larger project aiming to aid the evaluation of CQI in primary care by providing guidance on factors to include in evaluations and the selection of instruments for measuring these factors. The project includes a companion review of instruments measuring organizational, process, and individual-level factors [27], and development of a conceptual framework, the Informing Quality Improvement Research (InQuIRe) in primary care framework.

**Figure 1:**
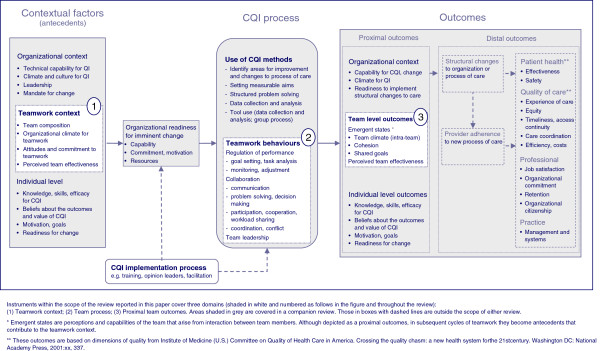
**Conceptual framework for defining the scope of the review – initial version of Informing Quality Improvement Research (InQuIRe) in primary care.** Instruments within the scope of the review reported in this paper cover three domains (shaded in white and numbered as follows in the figure and throughout the review): (**1**) Teamwork context; (**2**) Team process; (**3**) Proximal team outcomes. Areas shaded in grey are covered in a companion review. Those in boxes with dashed lines are outside the scope of either review. * Emergent states are perceptions and capabilities of the team that arise from interaction between team members. Although depicted as a proximal outcomes, in subsequent cycles of teamwork they become antecedents that contribute to the teamwork context. ** These outcomes are based on dimensions of quality from Institute of Medicine (U.S.) Committee on Quality of Health Care in America. Crossing the quality chasm: a new health system for the 21st century. Washington DC: National Academy Press, 2001:xx, 337.

Our initial framework is included in this paper (Figure 1) to illustrate the scope of the review of instruments and as the basis for assessing the coverage of available instruments. The framework reflects our initial synthesis of CQI theory, a summary of which is reported in the companion review of instruments [27]. In brief, we identified recurrent themes about the core components of CQI from landmark papers that stimulated adoption of CQI in healthcare (*e.g.,*[2,28-31]). We built on these themes, refining and adding concepts from the main bodies of CQI research and models of CQI (*e.g.,*[32-35]). We searched for models, frameworks, and theories intended to describe how context influences quality improvement and change (*e.g.,*[36-39]). From these sources, we extracted factors salient to primary care, grouping them thematically using existing models to guide categorization. Our analysis was augmented by frameworks for understanding implementation, learning, and innovation (*e.g.,*[40-43]) and models of teamwork (*e.g.,*[12,44-47]). Both sources contributed new factors and informed the final categorization and structure of our framework. Our analysis of instruments is used to integrate new factors and concepts into the framework. These refinements are reported in the taxonomies presented in the reviews of instruments. The final InQuIRe framework will be reported separately.

This review aims to provide guidance on the selection of instruments for measuring team-level factors in studies of CQI in primary care. The specific objectives are to: identify self-report instruments measuring team-level factors thought to modify the effect of CQI; determine how the factors measured have been conceptualized; develop a taxonomy for categorizing instruments based on our initial framework and new concepts arising from the review of instruments; use the taxonomy to categorize and compare the content of instruments, enabling assessment of the coverage of instruments suitable for evaluating CQI team function in primary care; appraise the methods of development and testing of existing instruments, and summarize evidence of their validity, reliability, and feasibility for measurement in primary care. We focus on self-report instruments because of their utility in quantitative studies examining the relationship between team context, team process, and outcomes. Alternative measurement methods, such as behavioral observation scales, require substantial resources and are generally not feasible for larger scale evaluation.

Our review differs from two existing reviews of instruments measuring teamwork [48,49] in its focus on QI in primary care. The existing reviews consider all forms of teamwork in healthcare, including a large number of instruments relevant only to clinical teams [48,49]. QI teams perform tasks and interact in ways that differ markedly from clinical work [50,51]. For example, collaborative problem solving in QI involves eliciting views on the underlying cause of problems, questioning existing ways of working, and integrating different perspectives to identify changes to care. In primary care, there may be particular challenges and risks in engaging these behaviors, especially in small practices where the same team works closely to deliver and improve care. Team climate (*e.g.*, trust, open communication, norms of decision-making) may have a heightened role in influencing team function [47], and practices often lack the broader organizational structures and resources that facilitate QI work [52-54]. By identifying instruments suitable for primary care, we aim to help researchers measure factors salient to understanding QI teamwork in this understudied context [55].

### Scope of the review: InQuIRe framework

**Figure 2:**
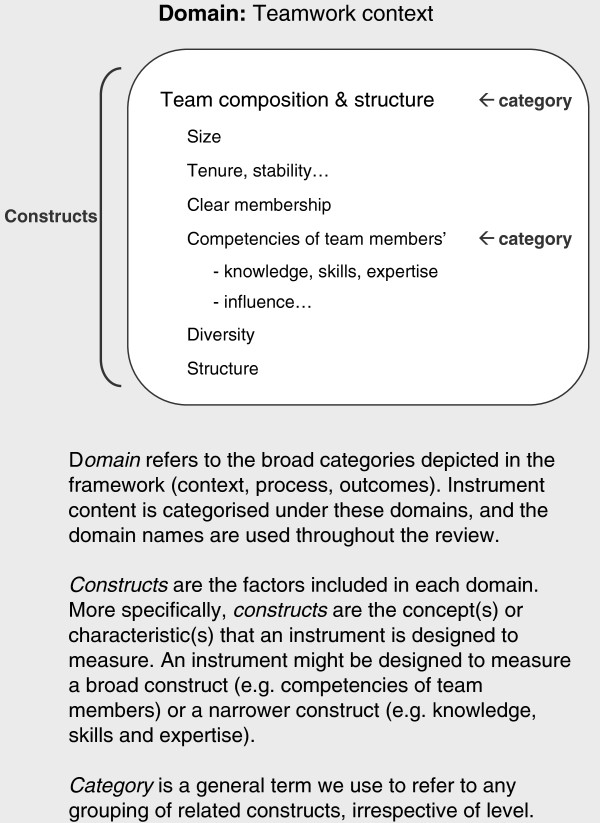
**Terms used to describe the framework, illustrated with content from the *Team composition and structure *category of the *Teamwork context *domain.**

This review covers instruments relevant to three domains of the InQuIRe framework: teamwork context, team process, and proximal team outcomes (shaded in white and numbered one to three in Figure 1). Teamwork context encompasses organizational, team, and individual factors thought to influence how the CQI team functions [44,46,51,56]. Within this domain, organizational climate for teamwork reflects shared perceptions of the extent to which the practice supports and rewards CQI teamwork through its policies, practices, procedures, and behavioral expectations [57]. Team process captures interactions between team members during the CQI process, focusing on behaviors required for members to function as an effective CQI team. These behaviors include knowledge sharing, collaborative problem solving, and making full use of members’ perspectives [45,58]. Proximal outcomes (emergent states and perceived team effectiveness) result from interactions between team members [44-46]. These outcomes include development of a shared understanding of team goals, commitment to achieving these goals, and perceptions of whether the climate within the team is safe for engaging in the behaviors required of CQI teams. In subsequent cycles of teamwork, these factors become antecedents that contribute to the teamwork context. Figure 2 illustrates terms used to describe the framework with an example from the taxonomy (see Additional file 1 for a glossary).

## Methods

**Figure 3:**
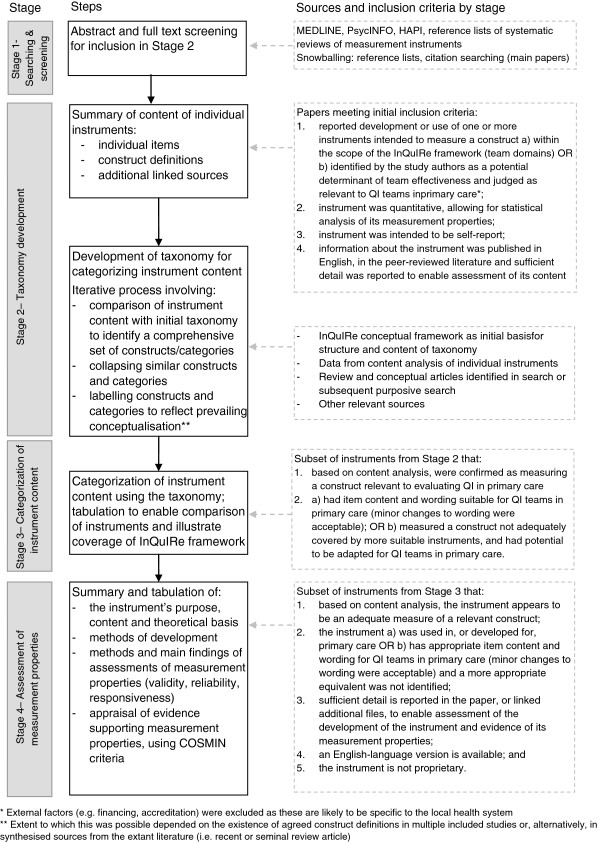
**Stages of data extraction and analysis for the review.** * External factors (e.g. financing, accreditation) were excluded as these are likely to be specific to the local health system. ** Extent to which this was possible depended on the existence of agreed construct definitions in multiple included studies or, alternatively, in synthesised sources from the extant literature (i.e. recent or seminal review article).

Figure 3 summarizes the four stages of the review and lists the criteria for selection of instruments at each stage.

### Stage one: searching and initial screening

#### Data sources and search methods

We searched MEDLINE (from 1950 through October 2011), PsycINFO (from 1967 through October 2011), and Health and Psychosocial Instruments (HaPI) (from 1985 through February 2012) using controlled vocabulary (thesaurus terms and subject headings) and free-text terms. Reference lists of identified systematic reviews were screened. Snowballing techniques (citation searches, reference lists) were used to trace the development and use of instruments included in Stage four. Searches were limited to articles published in English. Search terms and details of the search strategy are reported in Additional file 2. Abstracts and the full text of potentially relevant studies were screened for inclusion by one author (SB). Papers reporting development or use of a quantitative, self-report instrument measuring factors within the scope of the team domains of the InQuIRe framework were included for data extraction (see Figure 3 for initial inclusion criteria).

### Stage two: development of taxonomy for categorizing instrument content

#### Data extraction

One review author extracted data from all studies for all stages of the review (SB). To refine the data extraction guidance and data extraction, a research assistant extracted data from a sub-sample of studies (15 papers, comprising a 10% sample of the studies included in all stages of review). Across the two companion reviews double data extraction was performed on 30 papers. Data extracted for Stage two are described in Table 1. Data included descriptions of the instrument purpose and format, and data to facilitate analysis and categorisation of the content of each instrument (*e.g.*, constructs measured; theoretical basis).

**Table 1:** Data extracted at stage two

**Data extracted**	**Description**
Study characteristics	Study aims
	Study design (categorized as experimental, observational, instrument development, model development)
	Setting in which the instrument was used
Instrument source	Name of instrument
	Source paper for the instrument as cited by the authors
Instrument purpose	Purpose for which the instrument was used (descriptive, predictive or diagnostic, outcome measure/evaluative)
Instrument format	Number of items
	Response scale (Likert, ipsative, etc.); response options
Instrument content and theoretical basis	Constructs and dimensions measured
	Definitions of the constructs; additional description of the content required to illustrate how the construct had been operationalised (*e.g.,* sample items)
	Theoretical basis of the instrument and references cited for the theory

#### Taxonomy development

Methods for developing the taxonomy were based on the framework approach for qualitative data analysis [59]. This approach combines deductive methods (commencing with concepts and themes from an initial framework) with inductive methods (based on themes that emerge from the data). The InQuIRe framework (Figure 1) provided the initial structure and content for our taxonomy. We used content analysis of instruments to refine the taxonomy, aiming to ensure comprehensive coverage of relevant factors.

Instruments confirmed as relevant to one or more of the three domains of our framework were included for content analysis. To capture the breadth of potentially relevant constructs, we included instruments irrespective of whether item content was suitable for primary care (*i.e.,* items that did not resonate in primary care settings or inferred the team worked within a large organization, *e.g.*, ‘I would accept almost any job in order to keep working with this team’ [60]). The content of each instrument (items, sub-scales), and associated construct definitions, was compared with the taxonomy. Instrument content that matched constructs in the taxonomy was summarized using existing labels. The taxonomy was expanded to include missing constructs and new concepts, initially using the labels and descriptions reported by the instrument developers.

To ensure the taxonomy was consistent with the broader literature, we reviewed definitions extracted from review articles and conceptual papers identified from the search. We also searched for and used additional sources to define constructs when included studies did not provide a definition, a limited number of studies contributed to the definition, or the definition provided appeared inconsistent with our initial concept or that in other included studies. Following analysis of all instruments and supplementary sources, related constructs were grouped in the taxonomy. Overlapping constructs were collapsed, distinct constructs were assigned a label reflecting the broader literature, and the dimensions of constructs were specified to create the taxonomy.

### Stage three: categorization of instrument content

One author (SB) categorized the content of all instruments confirmed as measuring a relevant construct with item content and wording suitable for QI teams in primary care (Figure 3, sources and inclusion criteria for Stage 3). For constructs not adequately covered by suitable instruments, we included instruments with potential for adaptation (*e.g.,* instruments with a strong theoretical basis designed to measure attitudes toward clinical teamwork). Categorization of instrument content was based on the final set of items reported in the main report(s) for each instrument. A second author (MB) independently categorized the content of a sub-sample of ten instruments (12%), including all instruments where there was any uncertainty over categorization. The categorization was discussed to identify revisions to the taxonomy and confirm final categorization. All instruments were then re-categorized using the final taxonomy.

### Stage four: assessment of measurement properties

Information about the development and assessment of measurement properties of each instrument was extracted from the main and secondary reports (the latter focusing on studies of QI or change in primary care) (Table 2). These data included descriptions of the methods and findings of assessments of content and construct validity, reliability, and acceptability of the instrument to respondents.

**Table 2:** Data extracted at stage four

**Data extracted**	**Description**^**1**^
Instrument development	Methods used to generate items (*e.g.,* items derived from existing instruments; new items generated from data from interviews or comprehensive review of theory)
	Methods used to refine instrument
Administration & scoring	Method of administration (*e.g.,* self-administered, facilitated)
	Feasibility of administration (*e.g.,* researcher time, resources)
	Acceptability to respondents (*e.g.,* views on burden and complexity)
	Methods of scoring and analysis
Measurement properties	Methods and findings of assessments of:
	Content validity (*e.g.,* clear description of content domain and theoretical basis, expert assessment of items for relevance and comprehensiveness)
	Construct validity
	- Hypothesis testing (*e.g.,* whether scores on the instrument converge with measures of theoretically related variables, discriminate between groups, predict relevant outcomes)
	- Instrument structure (*e.g.,* using factor analytic methods)
	Reliability (*e.g.,* internal consistency, stability over time, inter-rater)
	Responsiveness
Other assessments	Interpretability (potential for ceiling and floor effects; guidance on what constitutes an important change or difference in scale scores)
	Generalizability (sampling methods, description of sample, and response rate reported)

^1^Definitions of the extracted measurement properties are provided in Additional file 3.

We used the COSMIN (COnsensus-based Standards for the selection of health status Measurement Instruments) checklist [61] to appraise the methods used for development and testing of instruments. The COSMIN criteria are intended for studies reporting measures of patient reported outcomes, however the checklist has strong evidence for its content validity [61,62] and mirrors the Joint Committee on Standards for Educational and Psychological Testing [63] (indicating relevance to measures other than health outcomes). Minor changes were incorporated based on guidance from organizational psychology [64-66], and reviews of organizational measures (*e.g.,*[67-69]).

Most instruments had undergone limited testing, and reporting of information required to complete the checklist was sparse. Because of the sparse data, for each instrument we tabulated a summary of the extent of evidence available for each property, and a description of the instrument’s development and testing. We used appraisal data to provide an overall summary of the methods used to develop and test each instrument.

## Results

### Screening and identification of unique instruments

**Figure 4:**
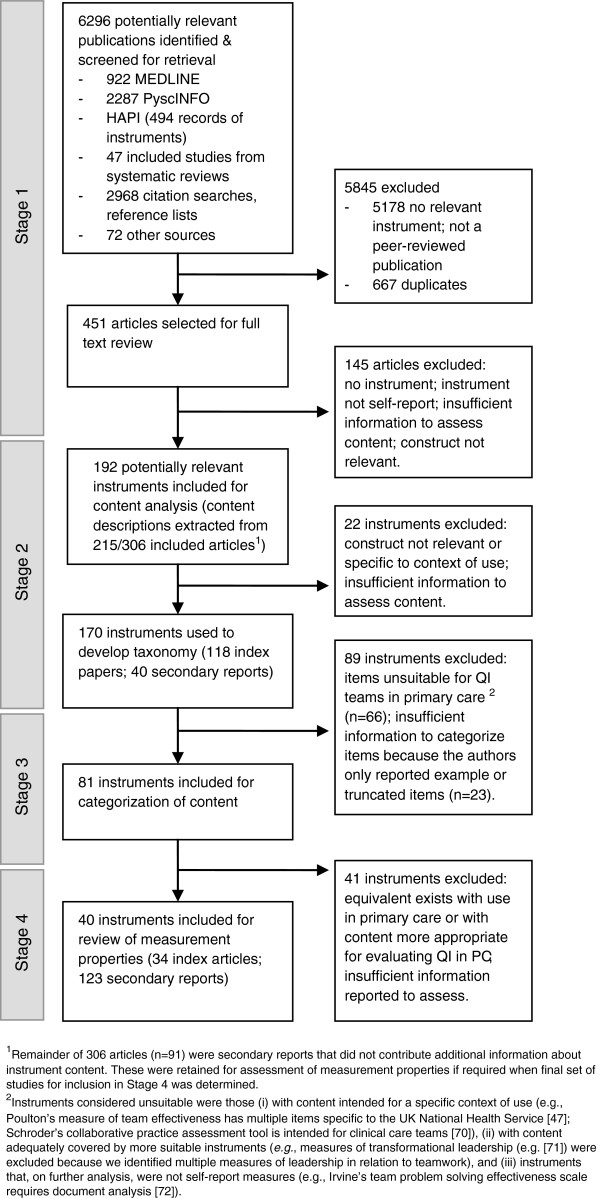
**Flow of studies and instruments through the review. **^1^Remainder of 306 articles (n=91) were secondary reports that did not contribute additional information about instrument content. These were retained for assessment of measurement properties if required when final set of studies for inclusion in Stage 4 was determined. ^2^Instruments considered unsuitable were those (i) with content intended for a specific context of use (e.g., Poulton’s measure of team effectiveness has multiple items specific to the UK National Health Service [47]; Schroder’s collaborative practice assessment tool is intended for clinical care teams [70]), (ii) with content adequately covered by more suitable instruments (e.g., measures of transformational leadership (e.g. [71]) were excluded because we identified multiple measures of leadership in relation to teamwork), and (iii) instruments that, on further analysis, were not self-report measures (e.g., Irvine’s team problem solving effectiveness scale requires document analysis [72]).

Our search identified 5,629 non-duplicate references, 5,178 of which were excluded following abstract screening (Figure 4). We reviewed the full text of 451 articles, and included 306 papers in stage two (Figure 4). Of these, 151 reported studies in healthcare, 49 of which were in primary care. Fifty-five papers had as their primary aim development of an instrument. Observational studies were most commonly reported in papers (n = 214), encompassing descriptive studies and tests of theoretical models. Twenty-one papers reported experimental designs. The remaining papers were conceptual. Individual papers reported between one and four potentially relevant instruments, contributing 339 reports of development or use of 170 unique instruments (Figure 4).

### Development of taxonomy and categorization of instrument content

The content of 170 instruments was analyzed to develop the taxonomy. The analysis led to elaboration and extension of the constructs in our initial framework, as reflected in the revised taxonomy (Additional file 4: Tables S3-S5). The main modifications are described under ‘Content and coverage of the domains of the InQuIRe framework.’ Minor changes were incorporated based on discussion arising from independent categorization of the subset of instruments. These changes involved adding lower level categories to some factors. There was consensus on most categories and no changes to the structure of the taxonomy.

We categorized the content of 81 instruments, of which 45 measured aspects of organizational context, 57 measured team process, and 59 measured proximal team outcomes (some instruments covered more than one domain, hence the total sums to >81). We excluded 89 instruments because items were unsuitable for QI teams in primary care or the authors reported example items only (see Figure 4 for examples [47,70-72]). The categorization of instrument content is reported by domain of the InQuIRe framework (Additional file 4: Tables S3-S5). This serves as a guide to content, however instruments vary substantially in comprehensiveness and the strength of their theoretical basis. In the next section, we review the instruments most suitable for measuring each domain. Used in combination with the results tables (Additional files 5: Table S7 and 6: Table S8), this analysis can be used to select instruments and to identify areas where better instruments are required.

### Content and coverage of domains of the InQuIRe framework

1) Teamwork context - enabling conditions and attitudes to teamwork

Table S3 (Additional file 4) reports the final taxonomy and categorization of instrument content for the teamwork context domain (box number ‘1’ in the InQuIRe framework, Figure 1).

Description of the teamwork context domain

We included instruments in this domain if they measured perceptions of task design, team composition and structure, or organizational climate for teamwork. These three categories comprise enabling conditions*,* a term adopted from Hackman’s model of team effectiveness [73,74]. The conceptualization of enabling conditions reflected in the taxonomy builds on models of team effectiveness (especially, [1,12,75]). Task design is an addition to the initial taxonomy, included to capture views about the nature of the team task (*e.g.,* perceptions of the need for individuals to work interdependently [75]).

We included individual attitudes, beliefs, and values as part of teamwork context. These factors reflect individual views about teamwork in general. They are distinct from emergent states, which are perceptions and cognitions of the team that emerge from interactions between members [44,45]. To reflect this, emergent states (and perceived team effectiveness) are categorized as proximal outcomes (box ‘3’ in the InQuIRe framework, Figure 1). In subsequent cycles of teamwork, these proximal outcomes contribute to teamwork context.

Comprehensive measures of enabling conditions

Wageman 2005 [73] and Campion 1993 [76,77] are the most comprehensive instruments measuring enabling conditions, covering aspects of task design (*e.g.,* interdependence, autonomy), team composition (*e.g.,* competencies, diversity), and organizational climate (*e.g.,* recognition, training). Neither instrument was developed in healthcare, nor have they had much prior use in healthcare contexts. Shortell 2004 [78] was the most comprehensive instrument developed for healthcare QI teams. It includes items measuring task design and organizational climate.

Measures of task design

We identified multiple instruments measuring aspects of task design (*e.g.,* Van den Bossche 2006a [79] and Van der Vegt 1998 [80] measure interdependence; Kirkman 1999a [81] and Langfred 2005 [82] measure autonomy; Edmondson 1999c [83] measures task significance). Items measuring perceptions of task meaningfulness or significance were relatively common in instruments developed for healthcare QI teams (*e.g.,* Mills 2004 [19], Alemi 2001 [84], Lukas 2009 [85], Schouten 2010 [86]).

Measures of team composition and structure

Few instruments measured perceptions of team composition. Those identified focused on perceptions of members’ task-related skill and knowledge (*e.g.,* Mills 2004 [19], Edmondson 1999c [83], Millward 2001 [87]). Diversity was typically measured using indices created from other team composition variables (*e.g.,* knowledge, status) rather than perception-based measures [11]. We identified two potentially relevant instruments measuring team structure. Bunderson 2010a [88] measures specialization (distinct and specialist roles), hierarchy (clear leadership), and formalization (goals and scheduling). Thylefors 2005 [89] measures team structure related to cross-functional clinical team effectiveness. It requires adaptation for QI teams.

Measures of organizational climate for teamwork

Shortell 2004 [78] and Lukas 2009 [85] include items measuring organizational climate for QI teamwork in healthcare, with an emphasis on external leadership support. The salience of leader support for QI was evident in instruments designed for healthcare QI teams; most included items measuring this construct (*e.g.,* Lemieux-Charles 2002a [8] Lubomski 2008 [90], Mills 2004 [19], Schouten 2010 [86]). Instruments measuring external leadership were included if they measured support for teamwork. Of these, instruments measuring support for team self-management were common, however items require adaptation because they reflect the manufacturing origin of this construct (*e.g.,* Manz 1987 [91]).

Measures of individual attitudes, beliefs and values about teamwork

These instruments typically examine views about team process and outcomes (*e.g.,* Dobson 2009a [92]), or beliefs about individual capability for teamwork (*e.g.,* Fulmer 2005 [93], Eby 1997 [94]). Most instruments from healthcare measured views about clinical teamwork. We included those with greatest potential for adaptation (*e.g.,* Fulmer 2005 [93], Baker 2010 [95], Heinemann 1999 [96]). Adaptation would involve rewording and formal assessment of the relevance of scales and items to QI teams. Scales measuring preferences for individual versus collective work (individualism/collectivism) indicate whether individuals value teamwork (*e.g.,* Driskell 2010 [97], Shaw 2000 [98]).

### 2) Team process (teamwork behaviors)

Tables S4a and S4b (Additional file 4) report the final taxonomy and categorization of instrument content within the team process domain (box number ‘2’ in the InQuIRe framework, Figure 1).

Description of the team process domain

Team processes are the *‘*interactions that take place among team members*’* to organize *‘*task-work to achieve collective goals*’*[45] and combine *‘*cognitive, motivational/affective, and behavioral resources*’*[44]. We included instruments in this domain if they measured teamwork behaviors or interactions between team members. We adapted existing frameworks for team processes [45] and teamwork behaviors [45,58] to develop categories within this domain. In the resulting taxonomy, instrument content was categorized as measuring regulation of team performance, collaborative behaviors and interpersonal processes, team maintenance, learning behaviors, and team leadership behaviors.

Regulation of team performance involves behaviors closely reflected in the CQI process. These include goal specification and planning, monitoring task performance, and adjusting to ensure goal attainment [58]. Monitoring teamwork process, feedback, and reflection are other important regulatory behaviors. Collaborative behaviors encompass the social processes that enable teams to develop shared knowledge and understanding, capitalize on diverse knowledge and perspectives, and work collaboratively to achieve goals [44,45,58,79,99]. Team maintenance was added to our taxonomy to delineate behaviors that enhance team viability (*e.g.,* conflict management, motivational behaviors). Most instruments measuring team process covered aspects of regulation, collaboration, and maintenance, hence they are described under a general heading of ‘team process.’

Measures of learning behaviors and team leadership behaviors were distinct from other instruments measuring process (so are described separately). Learning behaviors were an addition to our taxonomy, defined as *‘*an ongoing process of reflection and action, characterized by asking questions, seeking feed-back, experimenting, reflecting on results, and discussing errors or unexpected outcomes of actions.*’* ([83], p353). Team leadership includes behaviors of designated and informal leaders, the latter encompassing shared leadership and when individuals *‘…* emerge informally as a leader*’*[100].

Measures of team process developed for QI teams in healthcare

Schouten 2010 [86] focuses on regulation of performance through goal specification, planning, and monitoring progress toward goals. Alemni 2001 [84] covers similar concepts, but the response format limits psychometric analysis. Shortell 2004 [78] also emphasizes CQI methods used to plan and test changes, focusing on collaborative decision-making. Items were based on Lemieux-Charles 2002a [8], which measures collaborative behaviors during CQI. Wilkens 2006 [101] includes stand-alone scales measuring feedback and conflict. Other measures are tailored to specific projects or provide narrower coverage of constructs (see Lukas 2009 [85], Mills 2004 [19], Irvine 2002 [72], Duckers 2008 [18], Lubomski 2008 [90]).

Broad measures of team process

Hoegl 2001 [102] is a comprehensive measure of collaborative behaviors, with stand-alone scales measuring communication, coordination, workload sharing, and effort. Thompson 2009 [103] provides good coverage of collaborative problem solving and decision making behaviors. Hiller 2006 [104] measures the extent to which teams share responsibility for problem solving, goal specification, and planning. Anderson 1998 [105] focuses on team climate (an emergent state), but includes items measuring regulatory and collaborative behaviors thought to support innovation.

Scales measuring single constructs and complex aspects of team process

Stand-alone scales measuring specific team process constructs (*e.g.,* individual scales measuring decision making) were uncommon in healthcare. Instruments from non-healthcare settings address this gap. Relevant scales include measures of group process during meetings (Kuhn 2000 [106]), the extent to which teams reflect on their performance (reflexivity) (Schippers 2007 [107], Brav 2009 [108], de Jong 2010b [109]), information exchange and knowledge sharing (Bunderson 2010b [88], Staples 2008a [110]), cooperation (Brav 2009 [108]), and effort (de Jong 2010b [109]). Several instruments from non-healthcare settings address complex aspects of interpersonal processes, drawing on substantive bodies of theoretical and empirical research. Exemplars include Janssen 1999 [111] and Tjosvold 1986 [112]. These instruments measure constructive controversy behaviors that enable teams to draw out and integrate divergent knowledge and perspectives, through *‘*skilled discussion*’* of opposing views ([112], p127).

Measures of learning behaviors

Three instruments explicitly measured learning behaviors (Savelsbergh 2009 [113], van den Bossche 2006b [79], Edmondson 1999a [83]). Savelsbergh 2009 is a comprehensive measure of reflection, feedback, and communication behaviors that enable team members to develop a shared understanding of problems. Both Savelsbergh 2009 and van den Bossche 2006b build on theories of the role of discourse in developing shared cognitions [79,114]. Some instruments include direct questions about learning (*e.g., ‘*Members of my team actively learn from one another*’* Mathieu 2006 [115]), but are not measures of behaviors that support learning.

Measures of team leadership behaviors

Despite the perceived importance of leadership in teams, few instruments incorporated measures of leadership behavior. Lemieux Charles 2002a [8] is the most comprehensive measure for QI teams. Other relevant instruments cover shared leadership (*e.g.,* Hiller 2006 [104]), leader behaviors that empower teams (*e.g.,* Arnold 2000 [116]), and coaching (*e.g.,* Wageman 2005 [73]).

### 3) Proximal team outcomes emergent states and perceived team effectiveness

Tables S5a and S5b (Additional file 4) reports the final taxonomy and categorization of instrument content within the proximal team outcomes domain (box number ‘3’ in the InQuIRe framework, Figure 1).

Description of the proximal team outcomes domain

Instruments with content measuring emergent states or perceived team effectiveness were included in this domain. We adopted Marks’ view of emergent states as dynamic properties of a team that result from interaction between members. These proximal outcomes of team process include team-level cognitions, motivations, values, and beliefs [44-46]. Our taxonomy specifies common emergent states, focusing on those most pertinent to QI teams. These are categorized as beliefs about team capability, team knowledge, empowerment, cohesion, trust, team identification and commitment, team climate, and team norms.

We adopt three categories of perceived team effectiveness from Cohen and Bailey [75], and Hackman [73,74]: task performance outcomes, attitudinal outcomes (*e.g.,* satisfaction with the team, team viability), and behavioral outcomes (*i.e.,* changes to teamwork capability).

Multi-dimensional measures of emergent states including team climate

Climate is a multi-dimensional construct that typically focuses on a specific behavior (*e.g.,* climate for learning). Scales measuring individual emergent states can be combined to measure the dimensions of climate (or team context) most relevant to the target behavior. Anderson 1998 [105,117,118] is the most widely used example. Focusing on team climate for innovation, Anderson 1998 includes scales measuring innovation, quality orientation, psychological safety, and shared vision. Other examples include instruments measuring team context for QI (Wilkens 2006 [101]), context for team learning (*e.g.,* Edmondson 1999b [83]; van den Bossche 2006a [79]), and climate for creativity (Barczak 2010 [119]; Caldwell 2003 [120]). Each of these instruments contains scales suitable for measuring single emergent states. Millward 2001 [87] is a more general measure of factors thought to influence team effectiveness (knowledge, identification, cohesion).

Scales measuring single emergent state constructs

Many scales exist for measuring single emergent states, most from non-healthcare settings. We focused on those measuring factors inadequately covered by multi-dimensional instruments. Examples include instruments measuring empowerment (Kirkman 1999a [81]), cohesion (Carless 2000a [121]), team trust (Costa 2011 [122]), team identification and commitment (Janssen 2008 [123]; Bishop [124]), learning orientation (Bunderson 2003 [125]), and transactive memory systems (a team’s shared awareness of and ability to access its members’ knowledge) (Lewis 2003 [126]).

Measures of emergent states developed for QI teams in healthcare

Emergent states were measured in instruments developed for healthcare QI teams; however, items were often combined in a scale with items measuring process (*e.g.,* Shortell 2004 [78], Schouten 2010 [86], Mills 2004 [19]; Lemieux Charles 2002a [8]; Lubomski 2008 [90]). In general, this makes them unsuitable for measuring specific emergent states.

Measures of perceived team effectiveness

Most instruments measuring perceived effectiveness were written for the study in which they were used, with limited information about the basis for their content. We focused on dimensions of effectiveness that could not be measured objectively (*e.g.,* willingness to continue with the team) rather than perceptions of process of care or clinical outcomes. Of the instruments used in healthcare QI teams, Lemieux-Charles 2002b [8] includes items measuring satisfaction and viability, and Irvine 2000b [127] measures perceived success.

### Instrument characteristics, development, and measurement properties

In Stage four, we reviewed the development and measurement properties of 40 instruments with use or potential for use in primary care (unshaded in Tables S3 to S5, Additional file 4). This information is intended to aid selection of instruments based on evidence of reliability, validity, feasibility of administration, and acceptability to respondents.

We summarize characteristics of each included instrument (*e.g.,* purpose, dimensions as described by developers, items, and response scale) and provide examples of use relevant to CQI evaluation (Additional file 5). The characteristics of excluded instruments (shaded in grey in Tables S3 to S5, Additional file 4) and reasons for exclusion are reported in Additional file 6. Instruments are grouped by setting (as per Additional file 4: Tables S3 to S5), and then reported in alphabetical order.

Table S6 (Additional file 4) provides an overview of the development and testing of measurement properties for each instrument. It indicates the extent of evidence in the main report(s) and studies in relevant contexts, and is based on assessments described in Additional file 7: Table S9.

Most reports of instrument development included comprehensive definitions that reflected related research and theory. Reports of instruments from the psychology literature (25 of 40 instruments) provided this detail more frequently than reports from healthcare, and included more comprehensive descriptions of the intended measurement domain. Reports of instruments from healthcare varied widely in how comprehensively they described the intended measurement domain, and some of these instruments appeared to have no theoretical basis. Reporting of the process used to develop items was scant. Only a quarter of studies reported an independent assessment of content validity (*e.g.,* using an expert consensus process).

For most instruments, evidence of construct validity (*e.g.,* through hypothesis testing, analysis of the instrument’s structure) was derived from one or two studies (exceptions include Shortell 1991 [128], Jehn 2008a [99], Campion 1993 [76]). Few instruments had evidence that they were predictive of objective measures of team effectiveness, and only six instruments had evidence that they can differentiate between groups. Across all the criteria, Anderson 1998 (Team Climate for Innovation inventory) was an exception [105,117,118]. With extensive use and testing, it is the only included instrument with multiple tests of construct validity in healthcare (some in primary care). These include tests of whether the measure predicts relevant outcomes and differentiates between groups.

Most studies report Cronbach’s alpha (a measure of internal consistency or the ‘relatedness’ between items) for the scale (or subscales); however, this was sometimes done without checks to ensure that the scale was unidimensional [129,130]. About one-half of the studies assessed some form of inter-rater reliability, to determine within-team agreement. This property should be assessed when inferences are made at team level from individual-level data.

Most reports covered conceptual and analytical issues associated with measuring collective constructs, at a minimum specifying the level at which the construct was defined and interpreted. In most cases, analytical methods were appropriate for the level at which inferences were to be made.

Few studies reported the potential for floor and ceiling effects (which may influence ability to detect change in a construct [130]), and none of the studies provided guidance on what constitutes an important change in scores. The acceptability of the instrument to respondents was reported for less than a quarter of instruments. Missing items, assessment of whether items were missing at random or due to other factors, and the potential for response bias [130,131] were rarely reported.

## Discussion

In this review, we aim to provide guidance for researchers seeking to measure team-level factors that potentially influence the process and outcomes of CQI. We identified many potentially useful instruments and scales, with some novel examples from the psychology literature. Collectively, these instruments cover many of the factors hypothesized to contribute to QI success. Inclusion of these measures in CQI evaluations could help address some key questions about the extent to which team function and the context in which teams work influence CQI outcomes. Many of the included instruments had little or no prior use in healthcare settings, especially in primary care, and some instruments had limited evidence supporting their measurement properties. Additional testing of the measurement properties of most instruments in relevant contexts is therefore required. We consider these issues, and the potential application of available instruments, in relation to each domain of the InQuIRe framework. We then discuss key considerations for researchers in relation to the use and development of instruments.

### Measurement of teamwork context

Enabling conditions provide teams with the structure, resources, and broader organizational support required to do their work [73,74]. Attributes of the task itself provide the motivation for team members to work together and invest effort in achieving team goals. These conditions are important for CQI teams, and there are a broad range of instruments available for their measurement. Among these instruments are many that include short scales (two to three items) that could be used to measure single constructs. Combining these scales to measure factors salient to the context in which evaluations are conducted is the most efficient way to measure aspects of team context. Some of the constructs we included in our taxonomy are almost entirely absent from the QI literature, yet their inclusion could add to our understanding of CQI team function. For example, perceptions of interdependence may be an important determinant of active participation on a CQI team. While individuals may be supportive of teamwork in general (*i.e.*, display positive attitudes toward teamwork), they may believe that their contribution on a CQI team will go unrecognized (outcome interdependence), will not be influential in achieving the team goals (task interdependence), or will not help them achieve personal goals (goal interdependence) [79,80].

### Measurement of team process

Measures of team process are underutilized in studies of CQI, yet they have the potential to illuminate the extent to which teams enact formal teamwork behaviors and the extent to which these behaviors account for variance in CQI outcomes. A team’s ability to capitalize on its collective knowledge and expertise rests on its ability to use effective collaborative and interpersonal processes. Measures of team process are a measure of the fidelity with which the ‘teamwork’ component of CQI is implemented. They provide important explanatory data about why CQI interventions may work in some contexts and not others. They can also provide data for developing targeted interventions to enhance a team’s ability for CQI teamwork. Although there are a number of instruments that are potentially useful, there is an absence of evidence about the extent to which these self-report instruments measure actual behavior. Criterion measures for behavior involve observing teams and rating their behavior on a validated scale (*e.g.,* van Ginkel’s scale for rating ‘elaboration’ during team discussion [132]). Behavioral observation scales were excluded from the review because they require substantial resources (trained observers, some requiring analysis of qualitative data). An important area of inquiry would be to establish the construct validity of self-report measures in studies using observation scales as a criterion measure.

### Measurement of proximal team outcomes (emergent states, perceived team effectiveness)

The measurement of team climate for innovation - an emergent state - is widespread in healthcare, largely due to the popularity of Anderson and West’s Team Climate for Innovation (TCI) inventory [105]. It has a strong theoretical basis, and the body of evidence supporting the measurement properties of this instrument is unrivalled. It is relevant to CQI, yet we found no examples of its use in this context. The dimensions it measures are used to predict a team’s ability to innovate. There are many other emergent states of relevance to QI teams, and we encourage researchers to consider the multitude of short scales available for measuring other factors that shape the interpersonal context in which QI teams work. Instruments that measure context for team learning are a good example (*e.g.,* Edmondson 1999b [83], Van den Bossche 2006a [79]).

Measures of perceived team effectiveness (especially those measuring perceived task outcomes relating to process of care and patient outcomes) have a somewhat limited scope for application in healthcare where objective measures of outcomes are desirable. However, measures of satisfaction with team process, team viability, and improved team functioning are important. These outcomes may have implications for the delivery of care in primary care settings where the same people work together on QI and care teams. A recent study in primary care suggested that positive experiences of QI teamwork contributed to building teamwork capacity for delivering clinical care [133]. Team building is an important outcome of CQI that may enhance the effectiveness of care teams.

### Key considerations for researchers

Researchers using this review to select instruments for use in CQI projects or evaluations will need to weigh up the strengths of different instruments, taking into consideration suitability for the hypotheses they intend to test, acceptable respondent burden, and the availability of evidence supporting measurement properties in settings and conditions pertinent to their study. There is no single instrument suited to all purposes. Given the limited evidence of measurement properties for many instruments, the relevance of instrument content and length are key decision points. Surveys of health professionals typically have low response rates [134], which may introduce bias. Shorter instruments have been shown to achieve higher response rates [135]; however, as yet there is insufficient evidence to determine optimal questionnaire length to maximize response rate [134].

The review findings highlight two other areas that need careful consideration: ensuring conceptual clarity when selecting, interpreting, and developing instruments, and increasing consistency in how team-level factors are conceptualized in QI studies.

#### Ensuring conceptual clarity in measurement

Marks *et al.* describe the common practice of *‘*intermingling*’* items measuring emergent states with those measuring team processes. In their view, this leads to *‘*serious construct contamination*’*[45]. Construct contamination can occur when items measuring different constructs are included in a single scale and was apparent in some instruments we reviewed (*e.g.*, combining an item measuring process ‘the team gathers data from patients’ with an item measuring knowledge ‘the team is familiar with measurement’), Construct contamination makes instruments difficult to interpret, and may prevent synthesis of resulting evidence because it is unclear what construct is being measured. Our categorization reflects the content of the items in each instrument (Additional file 4: Tables S3 to S5), not how items are combined to form a scale (the latter is considered under ‘coverage of the framework’). Statistical tests of an instrument’s structure provide empirical evidence about whether items are related. However, a scale can be formed using factor analysis that does not measure a distinct or meaningful construct. Ideally, the scale’s content validity will be supported by a clear construct definition, *a priori* hypotheses about the relationship between items, and independent assessment of content. In the absence of such information, checking that items in a scale (or subscale) appear to measure a specific construct can guide selection and interpretation of instruments.

#### Increasing consistency in how team-level factors are conceptualized

While researchers have raised concerns about variability in how factors thought to influence CQI outcomes have been defined and measured [14], our findings suggest that many team-level factors are clearly and consistently defined in the teamwork literature arising from psychology. Moreover, many team concepts are underpinned by a body of empirical research and theory (*e.g.,*[44,46]). Adoption of these concepts in studies of CQI could accelerate our ability to develop an evidence base that is cumulative, enabling findings to be compared and synthesized across studies.

### Strengths and limitations

Although other reviews of teamwork instruments exist [48,49], to our knowledge, this is the first review to systematically collate and categorize instruments available for measuring team-level factors in an evaluation of CQI. Our broad, systematic search of the health and psychology literature enabled us to identify a diversity of potentially relevant instruments. Categorizing the content of included instruments using the developed taxonomy enabled direct comparison of instruments. The taxonomy provides a common language for describing instruments and the factors they measure, addressing some of the complexities researchers face when selecting instruments. By basing the taxonomy on a theoretically-based framework for evaluating CQI, we provide guidance on how the identified instruments could be included in an evaluation.

We used a broad search, but may have missed some instruments. We excluded instruments published in books, the grey literature (*e.g.,* theses), and proprietary sources, and those for which items were not reported in full in published sources. Our rationale for these exclusions was that neither the instruments nor information about their measurement properties is readily accessible to researchers. We excluded papers published in languages other than English. One author extracted data from the majority of studies. This approach was based on checks on a subset of studies that confirmed that there were no important discrepancies between two data extractors. One author developed the taxonomy and categorized the content of instruments (SB) with independent categorization from a second author (MB) for a subset of instruments, including those involving more complex decisions. Given the judgment required to make these decisions, alternative categorizations are possible. This is the first application of our taxonomy, and refinement is likely, especially as new research emerges on the factors that influence QI and change in primary care.

## Conclusions

Cross-functional teams are a core element of CQI, yet there is a paucity of evidence about how team-level factors influence the outcomes of CQI. In this review, we aimed to provide guidance to address some of the challenges researchers face in incorporating these factors in evaluations, particularly around the choice of measurement instruments for use in primary care. The conceptualization and measurement of factors that influence CQI is inherently complex, and primary care settings have unique features that heighten the importance of some factors. Yet, there is a need to address these complexities so that we can understand how teamwork influences CQI success and develop and test strategies to optimize team function. Although individual studies can make an important contribution, synthesis of multiple studies in different contexts is needed to identify the factors that contribute to successful CQI. To ensure that individual studies contribute to cumulative knowledge, consistency in the definition and measurement of factors is required. Such consistency enables comparison and synthesis of findings across studies, and underpins our ability to provide guidance for policy and practice on the implementation and outcomes of CQI.

## Competing interests

Heather Buchan is a member of the Implementation Science Editorial Board. The authors have no other competing interests.

## Authors’ contributions

SB and SG conceived the study with input from HB. SB designed the review, conducted the searching, screening, data extraction and analysis. SG, HB and MB provided input on the design, provided comment on the analysis and the presentation of results. SB drafted the manuscript and made subsequent revisions. All authors provided critical review of the manuscript. All authors read and approved the final manuscript.

## Supplementary Material

Additional file 1 Glossary of terms used in the review. Click here for file

Additional file 2 Search strategy and search terms. Click here for file

Additional file 3 Definition of measurement properties. Click here for file

Additional file 4 Tables reporting content of instruments measuring teamwork context (Table S3), team process (Tables S4a and b), and proximal team outcomes (Tables S5a and b), and overview of instrument development and assessment of measurement properties (Table S6). Click here for file

Additional file 5 Table summarizing the characteristics of instruments included for review of measurement properties (Stage four) (Table S7). Click here for file

Additional file 6 Table summarizing the characteristics of instruments included for categorization of content, but excluded from review of measurement properties (Stage four) (Table S8). Click here for file

Additional file 7 Table summarizing the development and measurement properties of instruments included in Stage four of the review (Table S9). Click here for file
