# Supplementary material for: Measuring team factors thought to influence the success of quality improvement in primary care: a systematic review of instruments
Source: Implement Sci. 2013 Feb 14;8:20. doi: 10.1186/1748-5908-8-20 (PMC3602018; doi:10.1186/1748-5908-8-20)
Supplement: Additional file 1 — Glossary of terms used in the review. [file 1748-5908-8-20-S1.pdf]

Glossary

Category	A term used informally in the review to describe any grouping of constructs within a domain.
Construct	Concept(s) or characteristic(s) that cannot be directly observed. Construct refers to both the concept that an instrument is designed to measure, and concepts in a theory. [1-2]
Dimension	A unique aspect of a construct. Constructs may have a single dimension or may be multidimensional. The dimensions of a construct should be predicted by theory and confirmed empirically. [3]
Domain	A broad grouping of constructs, which together form the components of a theory or a defined area of interest. [4]
Instrument	The combination of items used to measure one or more constructs. The instrument may or may not consist of stand-alone scales each measuring unique constructs (or subscales measuring dimensions of a construct). [1]
Item	A single question or statement used to measure a construct. Includes the item stem and response options. [2]
Scale	The set of items that measure a single underlying characteristic. These characteristics are either unique constructs or unique dimensions of a construct. [5]

References

1. Gliner JA, Morgan GA, NetLibrary Inc.: *Research methods in applied settings an integrated approach to design and analysis.* Mahwah, N.J.: Lawrence Erlbaum; 2000.
2. Joint Committee on Standards for Educational and Psychological Testing (U.S.), American Educational Research Association., American Psychological Association., Education. NCoMi: *Standards for educational and psychological testing.* Washington, DC: American Educational Research Association; 1999.
3. Di Iorio CK: *Measurement in health behavior: methods for research and education.* 1st edn. San Francisco: Jossey-Bass; 2005.
4. Miner JB: *Organizational Behavior 1 : Essential Theories of Motivation and Leadership.* Armonk: M.E. Sharpe Inc.; 2005.
5. Streiner DL, Norman GR: *Health measurement scales: a practical guide to their development and use.* 3rd edn. Oxford ; New York: Oxford University Press; 2003.
