# Supplementary material for: Measuring team factors thought to influence the success of quality improvement in primary care: a systematic review of instruments
Source: Implement Sci. 2013 Feb 14;8:20. doi: 10.1186/1748-5908-8-20 (PMC3602018; doi:10.1186/1748-5908-8-20)
Supplement: Additional file 2 — Search strategy and search terms. [file 1748-5908-8-20-S2.pdf]

## Additional file 2: search strategy and search terms

## Search strategy – additional information

The final set of terms (Box 1 and 2) was decided following scoping searches to test search terms for retrieval of known reports of instruments. The scoping searches identified one comprehensive review of measures of team performance [1]. We screened the reference list of this review and others identified from the database searches and other sources. This included systematic reviews of (i) measurement instruments, and (ii) studies of team effectiveness (e.g. reviews of observational studies measuring factors thought to influence team effectiveness). We used a simple search in the HaPI database, mapping the term 'team' to search titles, acronyms, descriptors, measure descriptors, sample descriptors, abstract and source.

Snowballing techniques were used to trace the development and use of instruments and to identify related conceptual papers. We identified the main publication(s) reporting initial development of instruments, screened the reference lists of these studies and conducted citation searches in ISI Web of Science citation databases or Scopus for more recent publications [2]. Snowballing searches were limited to the subset of instruments included in Stage 3 of the review.

### Box 1: controlled vocabulary and free-text terms used to search PsycINFO

1. (team or teams or teamwork).ti.
2. exp teams/
3. 1 or 2
4. sport?.mp.
5. exp Sports/
6. 4 or 5
7. 6 not 3
8. 3 not 7
9. (performance or performing).ti,ab.
10. (work or working).ti,ab.
11. (effective or effectiveness).ti,ab.
12. (function or functioning).ti,ab.
13. climate.ti,ab.
14. (innovation or innovative).ti,ab.
15. or/9-14
16. 8 and 15 [teamwork terms]
17. questionnaire?.mp.
18. (instrument or instruments or instrumentation).ti,ab.
19. (survey or model).ti,ab.
20. (measure or measures or measuring or measurement).ti.
21. (scale or scales).ti.
22. (assessment or assess or assessing).ti.
23. or/17-22 [measurement terms]
24. 16 and 23

## Box 2: controlled vocabulary and free-text terms used to search MEDLINE

1. (team or teams or teamwork).ti.
2. sport?.mp.
3. exp Sports/
4. 2 or 3
5. 1 not 4
6. (performance or performing).ti,ab.
7. (work or working).ti,ab.
8. (effective or effectiveness).ti,ab.
9. (function or functioning).ti,ab.
10. climate.ti,ab.
11. (innovation or innovative).ti,ab
12. or/6-11
13. 5 and 12 [teamwork terms]
14. questionnaire?.mp.
15. (instrument or instruments or instrumentation).ti,ab.
16. (survey or model).ti,ab.
17. (measure or measures or measuring or measurement).ti.
18. or/14-17 [measurement terms]
19. 13 and 18

References


1. Heinemann GD, Zeiss AM (Eds.): Team performance in health care: Assessment and development. New York, NY: Kluwer Academic/Plenum Publishers; 2002.
2. Falagas ME, Pitsouni EI, Malietzis GA, Pappas G: Comparison of PubMed, Scopus, Web of Science, and Google Scholar: strengths and weaknesses. *FASEB J* 2008, 22:338-342.
