# Supplementary material for: Measuring team factors thought to influence the success of quality improvement in primary care: a systematic review of instruments
Source: Implement Sci. 2013 Feb 14;8:20. doi: 10.1186/1748-5908-8-20 (PMC3602018; doi:10.1186/1748-5908-8-20)
Supplement: Additional file 3 — Definition of measurement properties. [file 1748-5908-8-20-S3.pdf]

Additional file 3: Definition of measurement properties

Property	Definition and description of assessments[1]
Validity	"The degree to which accumulated evidence and theory support specific interpretation of test scores entailed by proposed uses of a test." ([1] p184).[2]
Content validity	The extent to which the content of the instrument clearly and comprehensively reflects the construct it is intended to measure.<br><br>Assessment: typically expert or independent assessment of the instrument against a detailed definition of the construct to determine (i) appropriateness of content for intended purpose, (ii) extent to which individual items are relevant to the content domain, and (iii) extent to which the entire set of items comprehensively represents all dimensions of the construct [1-3].
Construct validity – hypothesis testing	The extent to which the instrument measures the construct intended based on accumulated evidence from testing hypotheses about (i) the association between scores on the instrument and theoretically related variables, and (ii) the difference in scores between groups expected to differ on the construct.<br><br>Hypothesis testing should assess whether scores on the instrument (i) 'converge' with related variables -including other measures of the same construct, (ii) 'discriminate' between groups expected to differ on the construct, (iii) predict relevant outcomes, and (iv) concur with scores on a criterion – or gold standard – measure of the construct (e.g. a long form of instrument). Hypotheses should be pre-specified and include the expected direction (positive or negative) and magnitude (absolute or relative) of correlation or difference between groups [1, 3].
Construct validity - structure	The extent to which items on the proposed scale (or subscales) relate to each other in a way that is consistent with the theoretically predicted dimensions of the construct [1].<br><br>Assessment: confirmatory factor analysis (CFA) to test a priori hypotheses about the relationship between items. In the absence of an a priori hypothesis about the dimensions of a construct, exploratory factor analysis of the instrument's structure may be used to (i) identify dimensions, (ii) assess the unidimensionality of scales or subscales to confirm that items can be summed before assessing internal consistency, and (iii) assess whether there are redundant items or items that relate poorly to the construct (i.e. during instrument development). Approaches based on item response theory may also be used.
Reliability[3]	The extent to which an instrument yields scores attributable to the 'true' score and not measurement error. When the 'true' score is unchanged, reliable instruments should produce reproducible scores in a range of conditions.<br><br>Assessments: (i) tests of whether the instrument yields consistent scores on items from the same scale (internal consistency – "the interrelatedness among the items" [4], p742), (ii) stable scores over time (test-retest reliability), and (iii) consistent scores with different raters (inter-rater reliability).

Property	Definition and description of assessments[1]
Other assessments	
Acceptability	Assessments of whether the instrument is acceptable to respondents.<br>Assessments: direct assessment of respondent views on burden and complexity of the questionnaire, or indirect assessment involving (i) time to complete (instructions and response time), (ii) response rate, and (iii) missing responses to items and whether there is potential for response bias.
Feasibility	Assessments of the feasibility of administering and scoring the instrument.<br>Assessments: time to administer, time to score or process data.
Level of analysis	The extent to which the content of the instrument, and the analysis and interpretation of resulting data, is consistent with the level at which the construct is defined.<br>Assessments: Clear statement of (i) the level at which the construct is conceptualised (e.g. group, organisation), and (ii) how the construct is conceptualised (e.g. as a 'shared' property which is meaningful only if there is within group consensus; as a property in which the extent of variation within groups is of interest). Instrument content, data analysis and interpretation is consistent with the conceptualisation of the construct.
Responsiveness	The extent to which an instrument detects changes over time, where changes are actually present. Responsiveness is a form of validity relating to change scores [3, 5].<br>Assessment: analogous to those used to assess construct validity [3, 5], focussing on change scores rather than cross sectional scores.
Interpretability	The extent to the instrument captures the full range of responses relevant to assessing the construct and can detect *important* changes or differences between groups.<br>Assessment: reporting of distribution of scores and potential for ceiling and floor effects; formal assessment of the smallest difference in scores considered important or meaningful (i.e. minimal important change or difference).[3, 5]
Generalisability	Reporting of information to enable assessment of the extent to which the findings about the instrument's measurement properties can be generalised.<br>Assessment: sample frame and selection described; response rate and analytical sample reported.

1. Multiple sources were used to identify, define and describe each property [1-2, 4-10]. Where the definition or description closely matches a particular source, the reference is provided in the text.
2. In this review, 'Test' is considered a synonym for 'instrument' or 'scale'.
3. The definition and description of categories of reliability is based on classical test theory (CTT). Similar concepts exist for generalisability theory (GT) and item response theory (IRT), however different techniques are used to assess measurement error [1]. Definition and description was limited to CTT approaches because they dominated the literature reviewed in this paper.

References

1. Joint Committee on Standards for Educational and Psychological Testing (U.S.), American Educational Research Association., American Psychological Association., Education. NCoMi: *Standards for educational and psychological testing.* Washington, DC: American Educational Research Association; 1999.
2. Di Iorio CK: *Measurement in health behavior: methods for research and education.* 1st edn. San Francisco: Jossey-Bass; 2005.
3. Mokkink L, Terwee C, Knol D, Stratford P, Alonso J, Patrick D, Bouter L, De Vet H: **The COSMIN checklist for evaluating the methodological quality of studies on measurement properties: A clarification of its content.** *BMC Medical Research Methodology* 2010, **10:**22.
4. Mokkink LB, Terwee CB, Patrick DL, Alonso J, Stratford PW, Knol DL, Bouter LM, de Vet HCW: **The COSMIN study reached international consensus on taxonomy, terminology, and definitions of measurement properties for health-related patient-reported outcomes.** *Journal of Clinical Epidemiology* 2010, **63:**737-745.
5. Streiner DL, Norman GR: *Health measurement scales: a practical guide to their development and use.* 3rd edn. Oxford ; New York: Oxford University Press; 2003.
6. Fitzpatrick R, Davey C, Buxton MJ, Jones DR: **Evaluating patient-based outcome measures for use in clinical trials.** *Health technology assessment (Winchester, England)* 1998, **2:**i-iv, 1-74.
7. Mannion R, Davies H, Scott T, Jung T, Bower P, Whalley D, McNally R: **Measuring and assessing organisational culture in the NHS (OC1)**. National Co-ordinating Centre for the National Institute for Health Research Service Delivery and Organisation Programme (NCCSDO) London 2008.
8. Mokkink LB, Terwee CB, Gibbons E, Stratford PW, Alonso J, Patrick DL, Knol DL, Bouter LM, De Vet HC: **Inter-rater agreement and reliability of the COSMIN (COnsensus-based Standards for the selection of health status Measurement Instruments) Checklist.** *BMC Med Res Methodol* 2010, **10:**82.
9. O'Leary-Kelly SW, J. Vokurka R: **The empirical assessment of construct validity.** *Journal of Operations Management* 1998, **16:**387-405.
10. Trochim WM: **The Research Methods Knowledge Base** [http://www.socialresearchmethods.net/kb/]
11. Mokkink LB, Terwee CB, Patrick DL, Alonso J, Stratford PW, Knol DL, Bouter LM, de Vet HC: **COSMIN checklist manual, version 6.0 (February 2010)**. Downloaded from: http://cosmin.nl 2010.
12. Malhotra MK, Grover V: **An assessment of survey research in POM: from constructs to theory.** *Journal of Operations Management* 1998, **16:**407-425.
13. Klein KJ, Conn AB, Smith DB, Sorra JS: **Is everyone in agreement? An exploration of within-group agreement in employee perceptions of the work environment.** *Journal of Applied Psychology* 2001, **Vol.86:**pp.
14. Klein KJ, Kozlowski SWJ: **From Micro to Meso: Critical Steps in Conceptualizing and Conducting Multilevel Research.** *Organizational Research Methods* 2000, **3:**211-236.
15. Chan D: **Functional relations among constructs in the same content domain at different levels of analysis: A typology of composition models.** *Journal of Applied Psychology* 1998, **83:**234-246.
16. Terwee CB, Bot SDM, de Boer MR, van der Windt D, Knol DL, Dekker J, Bouter LA, de Vet HCW: **Quality criteria were proposed for measurement properties of health status questionnaires.** *Journal of Clinical Epidemiology* 2007, **60:**34-42.
17. Hinkin T: **A brief tutorial on the development of measures for use in survey questionnaires.** *Organizational Research Methods* 1998, **1:**104-121.
