# Supplementary material for: Measuring team factors thought to influence the success of quality improvement in primary care: a systematic review of instruments
Source: Implement Sci. 2013 Feb 14;8:20. doi: 10.1186/1748-5908-8-20 (PMC3602018; doi:10.1186/1748-5908-8-20)
Supplement: Additional file 4 — Tables reporting content of instruments measuring teamwork context (Table S3), team process (Tables S4a and b), and proximal team outcomes (Tables S5a and b), and overview of instrument development and assessment of measurement properties (Table S6). [file 1748-5908-8-20-S4.pdf]

Table S3: Content of instruments measuring teamwork context (enabling conditions; individual attitudes) [1,2,3,4,5.]

	Alemni 2001 [1]	Anderson 1998 [2]	Hearnshaw 1998 [3]	Lukas 2009 [4]	Shortell 1991 [5]	Shortell 2004 [6]	Adler-Milstein 2011 [7]	Baker 2010[8]	Caldwell 2003 [9]	Campion 1993 [10]	Costa 2011 [11]	Dobson 2009a [12]	Duckers 2008 [13]	Edmondson 1999c [14]	Fulmer 2005 [15]	Gould 2002 [16]	Heinemann 1999 [17]	Irvine 2000a [18]	Irvine 2002 [19]	Lemieux-Charles 2002a [20]	Lewis 2003 [21]	Lichtenstein 1997 [22]	Lubomski 2008 [23]	Mills 2004 [24]	Millward 2001 [25]	Schouten 2010 [26]	Thylefors 2005 [27]	Allen 1998 [28]	Barczak 2010 [29]	Brav 2009 [30]	Bunderson 2010a [31]	Carless 2000b [32]	Driskell 2010 [33]	Eby 1997 [34]	Kirkman 1999a [35]	Kirkman 1999b [35]	Langfred 2005 [36]	Manz 1987 [37]	Mathieu 2006 [38]	Shaw 2000 [39]	Staples 2008a [40]	Staples 2008c [40]	van den Bossche 2006a [41]	van der Vegt 1998 [42]	Wageman 2005 [43]
	primary care						other health care																					non health care																	
ENABLING CONDITIONS																																													
Task design														#																															
Interdependence (task, goal, outcome)		X				X	X		X	X				X							X*	X			X		X										#					#	#	#	X
Autonomy (structural empowerment)				X		X			X	X			X										X												X	X*	#								X
Significance or meaningfulness	X	X		X		X				X				X										X		X		X							X										X
Complexity & achievability		X																																											X
Team composition & structure								X*						#																															
Size (appropriateness of)										X																		X																	X
Tenure, stability of membership, history working together							X																X	X																					X
Clear membership (bounded)							X																																						X
Competencies of team members’																																													
- knowledge, skills, expertise						X		X*		X		X*		X						X	X*		X	X	X			X	X		X														X
- influence (*e.g.*, ability to access external support/resources)				X		X				X													X					X																	
Diversity (knowledge, perspectives, profession, status etc)										X		X*																																	X
Structure (extent to which team is structured)																															#														
Organizational climate for teamwork														#																															
Perceived support for teamwork																																													
- external leadership (support from, style of)				X		X		X*		X													X	X		X						#				#		#							
- other organizational members				X	X	X				X										X			X	X																					
Collaboration / learning			X		X				X	X										X												X*													
Resources (including time, information, systems)				X		X			X					X						X			X	X	X	X																			X
Rewards and recognition for teamwork				X		X			X	X				X														X																	X
Training, education, external coaching				X		X			X	X				X																		X*													X
ANTECEDENTS (individual level)																																													
Attitudes, beliefs, values about teamwork								#				#				#																													
Team orientation (collective v. individual orientation; preference for teamwork)								X	X	X	X								X														#						X	#	X				
Beliefs about specific teamwork processes								X				X					X																X							X					
Belief about consequences & outcomes of teamwork										X	X	X				X	#													X			X							X					
Beliefs about teamwork capability (self-efficacy)												X			#																		X	#											
Commitment to specific team goals or project		X																X																											

[1] ‘a’ and ‘b’ are used to label instruments when multiple instruments are reported in a single paper. [2] Shading denotes instruments excluded from Stage four (these instruments not in Table S6 or Additional File 7). [3] An ‘X’ may represent a single item, multiple items or an entire scale. [4]A # indicates a focal construct measured by the instrument. For these instruments, constructs marked with an X* are measured in relation to the focal construct (**#**). For example, Baker 2010 is a measure of attitudes (the focal construct). It measures attitudes about team composition and leadership. [5] Few of the instruments had names assigned by their developers; therefore, we used the first authors’ name and year of publication from the first of the main reports.

Table S4a: Content of instruments measuring team process (healthcare settings) [1,2,3,4,5]

	Alemni 2001 [1]	Anderson 1998 [2]	Lukas 2009 [4]	Shortell 1991 [5]	Shortell 2004 [6]	Adler-Milstein 2011 [7]	Baker 2010 [8]	Caldwell 2003 [9]	Campion 1993 [10]	Costa 2011 [11]	Deeter-Schmelz 2003a [44]	Deeter-Schmelz 2003b [44]	Dobson 2009b [12]	Duckers 2008 [13]	Edmondson 1999a [14]	Edmondson 1999b [14]	Fulmer 2005 [15]	Irvine 2000a [18]	Irvine 2000b [18]	Irvine 2002 [19]	Lemieux-Charles 2002a [20]	Lewis 2003 [21]	Lichtenstein 1997 [22]	Lubomski 2008 [23]	Mills 2004 [24]	Millward 2001 [25]	Schouten 2010 [26]	Thompson 2009 [45]	Thylefors 2005 [27]	Vinokur-Kaplan 1995 [46]	Wilkens 2006 [47]
	primary care					other health care																									
Regulation of team performance																															
Goal specification and planning																															
- problem/task analysis	X	X	X								X				X						X						X				
- goal specification	X		X		X						X										X						X				
- planning and strategy formulation	X				X	X					X						X*			?			X		X		X				
Monitoring performance and adjustment							X*																								
- monitoring progress toward goals (*e.g.,* data collection, analysis)	X	X	X		X	X									X									X	X		X				
- monitoring team processes		X				X									X		X*			X											
- feedback and reflection on performance		X	X		X	X		X		X	X				X		X*			X							X	X			X
- seeking external ideas, feedback & support	X		X				X*								X									X	X		X				
- adaptation		X	X		X	X		X		X	X	X*			X		X*									X		X			
- backing up behaviors		X					X*			X																					
Collaborative behaviors and interpersonal processes																															
Communication				X	X	X	X*			X			X	X	X		X*			X			X		X	X		X			
- information exchange & knowledge sharing		X	X	X		X	X*	X	X	X	X	X*								X	X		X			X				X	X
Constructive controversy		X		X											X					X			X					X			X
Collaborative problem solving & decision making	X	X	X	X	X	X		X	X	X	X		X		X		X*		X	X	X		X			X		X		X	
Participation, cooperation & workload sharing		X	X	X	X	X	X*	X	X	X				X		X	X*	X		X	X		X	X	X	X		X		X	
- making full use of members capabilities & perspectives		X		X	X								X			X					X					X		X		X	
- effort	X				X																X							X		X	
Coordination						X								X								X							X		
Conflict		X					X*													X		X		X							#
Team maintenance																															
Conflict management behaviors				X				X					X		X		X*							X	X			X			
Motivation and confidence building																												X			X
Affect management																															
Learning behaviors		X									X				#													X			
Leadership behaviors (intra-team; designated or informal leader)																					X										
Coaching (for task performance or team process)																					X								X		
Empowering				X																	X										
Shared & emergent leadership																	X*			X							X		X		
Leading by example (*e.g.,* modeling, high self standards)							X*																								
Other style or type (*e.g.,* authentic, person or task centered)				X			X*																		X		X		X		

[1] 'a' and 'b' are used to label instruments when multiple instruments are reported in a single paper.

[2] Shading denotes instruments excluded from Stage four: review of measurement properties (hence, they do not appear in Table S6 or Additional File 7).

[3] An 'X' may represent a single item, multiple items or an entire scale.

[4] A # indicates that this is a focal construct measured by this instrument. For these instruments, constructs marked with an X* are measured in relation to the focal construct (**#**). For example, Baker 2010 is a measure of attitudes (the focal construct). It measures attitudes about aspects of team process, but is not a measure of team process (i.e. it does not measure use of specific team process behaviors)

[5] Few of the instruments had names assigned by their developers; therefore, we used the first authors' name and year of publication from the first of the main reports.

Table S4b: Content of instruments measuring team process (non healthcare settings) [1,2,3,4]

	Allen 1998 [28]	Arnold 2000 [48]	Barczak 2010 [29]	Brav 2009 [30]	Bunderson 2003 [49]	Bunderson 2010a [31]	Bunderson 2010b [31]	Coyle-Shapiro 2003 [50]	de Jong 2010a [51]	Driskell 2010 [33]	Eby 1997 [34]	Edmondson 1999c [14]	Hiller 2006 [52]	Hoegl 2001 [53]	Janssen 1999 [54]	Jehn 2008a [55]	Kuhn 2000 [56]	Manz 1987 [37]	Mathieu 2006 [38]	Savelsbergh 2009 [57]	Schippers 2007 [58]	Seers 1989 [59]	Staples 2008a [40]	Tjosvold 1986 [60]	Van den Bossche 2006b [41]	Wageman 2005 [43]
	non health care																									
Regulation of team performance																										
Goal specification and planning																										
- problem/task analysis													X						X	X	X			X		
- goal specification	X					X						X	X					X*	X		X					
- planning and strategy formulation	X					X					X*		X							X	X					
Monitoring performance and adjustment																										
- monitoring progress toward goals (*e.g.,* data collection, analysis)	X	X*							X									X*		X	X					
- monitoring team processes		X*							X									X*		X	X					X
- feedback and reflection on performance		X*		X					X				X					X*		X	**#**	X				X
- seeking external ideas, feedback & support	X			X																X						
- adaptation	X								X											X	X					
- backing up behaviors		X						X					X	X								X				
Collaborative behaviors and interpersonal processes																										
Communication	X										X*			X	X				X	X	X	X		X	X	
- information exchange & knowledge sharing	X	X*		X			**#**							X					X	X		X	**#**	X	X	X
Constructive controversy										X*				X	**#**	X	X			X				**#**	X	
Collaborative problem solving & decision making	X	X*	X							X*	X*		X	X	X		**#**		X	X	X	X		X	X	
Participation, cooperation & workload sharing	X	X*		X			X	X	X		X*		X	X			X					X		X	X	X
- making full use of members capabilities & perspectives	X												X							X						
- effort							X	X	X					X												X
Coordination											X*		X	X												
Conflict														X		**#**										X
Team maintenance																										
Conflict management behaviors	X										X*			X	X	X									X	X
Motivation and confidence building		X*		X				X					X	X				X*	X							X
Affect management		X*											X													
Learning behaviors					X														X	**#**	X			X	**#**	X
Leadership behaviors (intra-team; designated or informal leader)																	X									
Coaching (for task performance or team process)		X											X													X
Empowering		**#**																								X
Shared & emergent leadership											X*		**#**													X
Leading by example (*e.g.,* modeling, high self standards)		X																								
Other style or type (*e.g.,* authentic, person or task centered)		X																								

[1] 'a' and 'b' are used to label instruments when multiple instruments are reported from a single paper.

[2] Shading denotes instruments excluded from Stage four - review of measurement properties (hence, they do not appear in Table S6 or Additional File 7).

[3] An 'X' may represent a single item, multiple items or an entire scale.

[4] A # indicates that this is a focal construct measured by this instrument. For these instruments, constructs marked with an X* are measured in relation to the focal construct (**#**). For example, Arnold 2000 is a measure of leadership behaviors (the focal construct). It measures leadership behaviors intended to support various team processes. It does not measure the team's use of these processes.

[5] Few of the instruments had names assigned by their developers; therefore, we used the first authors' name and year of publication from the first of the main reports.

Table S5a: Content of instruments measuring proximal team outcomes (healthcare settings) [1,2,3,4,5]

	Alemni 2001 [1]	Anderson 1998 [2]	Lukas 2009 [4]	Shortell 1991 [5]	Shortell 2004 [6]	West 2003 [61]	Adler-Milstein 2011 [7]	Caldwell 2003 [9]	Campion 1993 [10]	Costa 2011 [11]	Deeter-Schmelz 2003b [44]	Dobson 2009a [12]	Dobson 2009b [12]	Duckers 2008 [13]	Edmondson 1999b [14]	Irvine 2000a [18]	Irvine 2000b [18]	Irvine 2002 [19]	Lemieux-Charles 2002a [20]	Lemieux-Charles 2002b [20]	Lewis 2003 [21]	Lichtenstein 1997 [62]	Lichtenstein 2004 [63]	Lubomski 2008 [23]	Mills 2004 [24]	Millward 2001 [25]	Schouten 2010 [26]	Thompson 2009 [45]	Thylefors 2005 [64]	Tucker 2007 [65]	Vinokur-Kaplan 1995 [46]	Wilkens 2006 [47]
	primary care						other health care																									
EMERGENT STATES (MEDIATORS)																																
Beliefs about team capability																																
Efficacy			X		#										#				X					X	X							
Potency			X						X	X					X											X						
Knowledge (collective, shared)																																
Mental models (shared understanding of team process, team task or both)																																
- goal clarity (shared understanding of goals)		X			X		X						X											X	X	X	X					
- role clarity (shared understanding of roles, including leadership roles)						#						X*		X								X				X	X					
- task clarity (shared understanding of tasks & strategies)		X		X			X														X											
Transactive memory systems (shared understanding of and ability to use member knowledge & expertise)																					#					X						
Empowerment (perceptions of; psychological)			X		X																		X									
Cohesion																																
- interpersonal/social cohesion		X		X			X		X				X												X	X						
- task cohesion (shared commitment to goal or task)		X			X			X			X		X						X					X	X	X	X		X			
- group pride																										X						
Trust (trustworthiness, propensity to trust)		X		X			X			#			X		X						X											X
Psychological safety (non-threatening & safe for risk taking)		X		X				X							#							X			X							#
Team identification (affiliation with a specific team; individual commitment to the team)										X	X	X				#								X		X						
Team climate (intra-team)		#								X																						
Focus on innovation and improvement		X						#																			X					X
Quality orientation		X		X														X														
Learning orientation (learning v. performance focus)								X																								#
Valuing differences, respect, openness		X		X			X	X		X			X		X				X					X	X	X		X				X
Team norms (behavioral expectations)		X						X	X	X	#				X			X	X			X			X	X			X			X
PERCEIVED TEAM EFFECTIVENESS																																
Task performance outcomes																																
Perceived task performance																																
- team member perception (*e.g.,* perceived impact)	X				X								X				#			X		X			X	X				X		
- external perception (manager, consumer etc)																																
Attitudinal outcomes																																
Team viability (willingness to continue with team)																																
Satisfaction with team (task performance, team process)																			X	X		X									X	
Ongoing commitment to team																				X												
Behavioral outcomes (team functioning)																					X	X			X	X	X					

[1] 'a' and 'b' are used to label instruments when multiple instruments are reported from a single paper.

[2] Shading denotes instruments excluded from Stage four - review of measurement properties (hence, they do not appear in Table S6 or Additional File 7).

[3] An 'X' may represent a single item, multiple items or an entire scale.

[4] A # indicates that this is a focal construct measured by this instrument. For these instruments, constructs marked with an X* in this or other tables are measured in relation to the focal construct (**#**).

[5] Few of the instruments had names assigned by their developers; therefore, we used the first authors' name and year of publication from the first of the main reports.

Table S5b: Content of instruments measuring proximal team outcomes (non healthcare settings) [1,2,3,4,5]

	Allen 1998 [28]	Arnold 2000 [48]	Barczak 2010 [29]	Bishop 2005 [66]	Brav 2009 [30]	Bunderson 2003 [49]	Bunderson 2010a [31]	Carless 2000a [67]	Coyle-Shapiro 2003 [50]	de Jong 2010b [51]	Edmondson 1999c [14]	Guzzo 1993 [68]	Hoegl 2001 [53]	Janssen 1999 [54]	Janssen 2008 [69]	Jehn 2008a [55]	Jehn 2008b [55]	Jehn 2008c [55]	Kirkman 1999a [35]	Kuhn 2000 [56]	Mathieu 2006 [38]	Savelsbergh 2009 [57]	Seers 1989 [59]	Staples 2008b [40]	Tjosvold 1986 [60]	Van den Bossche 2006a [41]	Wageman 2005 [43]
	non health care																										
EMERGENT STATES (MEDIATORS)																											
Beliefs about team capability																											
Efficacy																	X										
Potency									X			#							X							#	X
Knowledge (collective, shared)																											
Mental models (shared understanding of team process, team task or both)	X																										
- goal clarity (shared understanding of goals)	X						X				X		X														X
- role clarity (shared understanding of roles, including leadership roles)	X						X																				
- task clarity (shared understanding of tasks & strategies)	X																						X				
Transactive memory systems (shared understanding of and ability to use member knowledge & expertise)																											
Empowerment (perceptions of; psychological)																			#								
Cohesion																											
- interpersonal/social cohesion			X					#	X				X				X									#	X
- task cohesion (shared commitment to goal or task)								#	X				X												X	#	
- group pride				X					X				X														X
Trust (trustworthiness, propensity to trust)			#						#	#							X				X			#		X	
Psychological safety (non-threatening & safe for risk taking)			X		X																	X			X	#	
Team identification (affiliation with a specific team; individual commitment to the team)	X			#											#												X
Team climate (intra-team)									#																		
Focus on innovation and improvement			#			X																X					X
Quality orientation	X	X*	X																			X					
Learning orientation (learning v. performance focus)						#																					
Valuing differences, respect, openness			X														X				X	X			X	X	
Team norms (behavioral expectations)	X									X						X					X	X				X	X
PERCEIVED TEAM EFFECTIVENESS																											
Task performance outcomes																											
Perceived task performance																											
- team member perception (*e.g.,* perceived impact)	X																		X								X
- external perception (manager, consumer etc)																											
Attitudinal outcomes																											
Team viability (willingness to continue with team)																		#									X
Satisfaction with team (task performance, team process)	X												X	#	X			X		X							X
Ongoing commitment to team															X												
Behavioral outcomes (team functioning)																											

[1] 'a' and 'b' are used to label instruments when multiple instruments are reported from a single paper.

[2] Shading denotes instruments excluded from Stage four - review of measurement properties (hence, they do not appear in Table S6 or Additional File 7).

[3] An 'X' may represent a single item, multiple items or an entire scale.

[4] A # indicates that this is a focal construct measured by this instrument. For these instruments, constructs marked with an X* are measured in relation to the focal construct (**#**). For example, Arnold 2000 is a measure of leadership behaviors (the focal construct). It measures leadership behaviors that emphasize quality (quality orientation). It does not measure whether the team has a quality orientation.

[5] Few of the instruments had names assigned by their developers; therefore, we used the first authors' name and year of publication from the first of the main reports.

Table S6: Overview of instrument development and assessments of measurement properties reported in included studies [1, 2]

primary care

	Anderson 1998 [2]	Hearnshaw 1998 [3]	Lukas 2009 [4]	Shortell, 1991 [5]	Shortell 2004 [6]
Development and content validity					
- clear description of content domain	✓✓	✓	✓	✓✓	✓
- clear description of theoretical basis	✓✓	✓	✓	✓✓	✓
- item development process reported	✓✓	X	X	✓	X
- formal assessment of content validity	✓✓	X	?	✓	?
Construct validity					
Hypothesis testing					
- hypotheses formulated a priori	✓✓	NA	✓	✓✓	✓
- predictive of related variables	✓✓	X	✓	✓✓	X
- association with other variables	✓✓	X	✓	✓	✓
- differences between groups	✓	X	X	X	X
Structural					
- exploratory	✓✓	X	✓	✓	✓
- confirmatory	✓✓	X	X	X	X
Reliability					
Internal consistency	✓✓	X	✓	✓	✓
Stability over time	✓	X	X	X	X
Other (inter-rater reliability)	✓✓	X	X	X	X
Other assessments					
Acceptability (assessed or inferred - response rate, missing items)	✓	X	X	X	X
Feasibility (completion time, other assessments)	✓	X	X	✓	X
Level of analysis considered	✓✓	✓	✓	✓✓	✓
Interpretability (ceiling/floor effects; important change or difference)	✓	X	✓	✓	✓
Responsiveness	X	X	X	X	X
Generalizability (sampling & response rate reported)	✓	✓	✓	✓	X

other health care

	Caldwell 2003 [9]	Campion 1993 [10]	Costa 2011 [11]	Dobson 2009a [12]	Dobson 2009b [12]	Edmondson 1999a [14]	Edmondson 1999b[14]	Lemieux-Charles 2002a [20]	Lemieux-Charles 2002b [20]	Lewis 2003 [21]	Lichtenstein 1997 [62]	Lubomski 2008 [23]	Mills 2004 [24]	Millward 2001 [25]	Schouten 2010 [26]	Thompson 2009 [45]	Wilkens 2006 [47]
Development and content validity																	
- clear description of content domain	✓	✓✓	✓✓	✓	X	✓✓	✓✓	✓✓	✓✓	✓✓	✓	✓	✓	✓	✓✓	X	✓✓
- clear description of theoretical basis	✓	✓✓	✓✓	✓	X	✓✓	✓✓	✓✓	✓✓	✓✓	✓✓	✓	✓	✓	✓✓	X	✓✓
- item development process reported	✓	✓	✓✓	✓	X	✓	✓	✓	X	✓	✓	X	X	✓	✓	✓	✓
- formal assessment of content validity	X	?	✓✓	✓	?	?	?	?	?	✓	?	?	X	?	✓	X	X
Construct validity																	
Hypothesis testing																	
- hypotheses formulated a priori	✓	✓	✓	✓	NA	✓✓	✓✓	✓	✓	✓	✓✓	✓	✓	?	NA	✓	✓
- predictive of related variables	X	✓	X	X	X	✓	✓	X	X	X	X	✓	X	X	X	X	X
- association with other variables	✓	X	✓	X	X	✓	✓	✓	✓	✓	✓✓	✓	X	✓	X	✓	✓
- differences between groups	X	X	X	✓	X	X	X	X	X	✓	X	X	✓	X	X	X	✓
Structural																	
- exploratory	✓	✓	✓	X	X	✓	✓	✓	X	X	✓	X	✓	X	✓	✓	X
- confirmatory	✓	X	✓	X	X	X	X	X	X	✓	X	X	X	✓	X	X	X
Reliability																	
Internal consistency	✓	✓	✓	X	X	✓	✓	✓	✓	✓	✓	✓	X	✓	✓	✓	✓
Stability over time	X	X	X	X	X	X	X	X	X	X	X	✓	X	X	X	X	X
Other (inter-rater reliability)	X	✓	✓	X	X	✓	✓	✓	X	✓	✓	X	X	✓	X	X	X
Other assessments																	
Acceptability (assessed or inferred - response rate, missing items)	X	X	X	X	X	X	X	X	X	X	X	✓	X	X	✓	X	X
Feasibility (completion time, other assessments)	X	X	X	X	X	X	X	X	X	X	X	✓	X	X	✓	✓	X
Level of analysis considered	✓	✓✓	✓✓	X	X	✓✓	✓✓	✓	✓	✓	✓	?	✓	✓	✓	✓	X
Interpretability (ceiling/floor effects; important change or difference)	X	✓	✓	✓	✓	✓	✓	X	X	X	✓	X	X	X	✓	✓	✓
Responsiveness	X	X	X	X	X	X	X	X	X	X	X	✓	X	X	X	X	X
Generalizability (sampling & response rate reported)	✓	✓	✓	✓	✓	✓	✓	✓	✓	✓	✓	X	✓	✓	✓	✓	✓

non health care

	Arnold 2000 [48]	Bunderson 2003 [49]	Bunderson 2010a [31]	Coyle-Shapiro, 2003 [50]	Driskell 2010 [33]	Edmondson 1999c [14]	Hiller 2006 [52]	Hoegl 2001 [53]	Janssen 1999 [54]	Jehn 2008a [55]	Kirkman 1999a [35]	Kuhn 2000 [56]	Savelsbergh 2009 [57]	Schippers 2007 [58]	Tjosvold 1986 [60]	Van den Bossche 2006a [41]	Van den Bossche 2006b [41]	Wageman 2005 [43]
Development and content validity																		
- clear description of content domain	✓✓	✓✓	✓✓	✓	✓✓	✓✓	✓	✓	✓✓	✓✓	✓✓	✓✓	✓✓	✓✓	✓✓	✓✓	✓✓	✓✓
- clear description of theoretical basis	✓	✓✓	✓✓	✓	✓✓	✓✓	✓	✓	✓✓	✓✓	✓✓	✓✓	✓✓	✓✓	✓✓	✓✓	✓✓	✓✓
- item development process reported	✓✓	X	X	X	✓	✓	✓	X	✓	✓	✓	X	✓✓	✓	X	✓	✓	✓
- formal assessment of content validity	X	X	X	X	X	?	✓	X	X	X	X	X	✓	✓	X	✓	✓	X
Construct validity																		
Hypothesis testing																		
- hypotheses formulated a priori	✓	✓	✓	✓	✓	✓✓	✓	✓	✓	✓	✓	✓	✓	✓	✓	✓	✓	✓
- predictive of related variables	X	✓	X	X	X	✓	X	X	X	X	X	X	X	X	X	X	X	X
- association with other variables	✓	✓	✓	✓	✓	✓	✓	✓	✓	✓	✓	X	✓	✓	✓	✓	✓	✓
- differences between groups	X	X	X	X	X	X	X	X	X	X	X	X	X	X	X	X	X	✓
Structural																		
- exploratory	X	X	X	✓	✓	✓	X	✓	✓	✓	✓	X	✓	✓	✓	✓	✓	X
- confirmatory	✓	X	✓	X	X	X	✓	X	X	X	X	X	✓	✓	X	X	X	X
Reliability																		
Internal consistency	✓	✓	✓	✓	✓	✓	✓	✓	✓	✓	✓	✓	✓	✓	✓	✓	✓	✓
Stability over time	X	X	X	X	X	X	X	X	X	X	X	X	X	X	X	X	X	X
Other (inter-rater reliability)	X	✓	✓	X	X	X	✓	✓	X	✓	X	X	X	✓	X	✓	✓	✓
Other assessments																		
Acceptability (assessed or inferred - response rate, missing items)	X	X	X	X	X	X	X	X	X	X	X	X	X	X	X	X	X	X
Feasibility (completion time, other assessments)	X	X	X	X	X	X	X	X	X	X	X	X	X	X	X	X	X	X
Level of analysis considered	X	✓✓	✓✓	X	X	✓	✓	✓	X	✓	✓	X	✓	✓	X	✓	✓	✓
Interpretability (ceiling/floor effects; important change or difference)	✓	✓	✓	✓	✓	✓	✓	✓	✓	✓	✓	X	✓	✓	X	✓	✓	✓
Responsiveness	X	X	X	X	X	X	X	X	X	X	X	X	X	X	X	X	X	X
Generalizability (sampling & response rate reported)	X	✓	✓	✓	X	✓	X	X	✓	X	✓	✓	✓	X	X	✓	✓	X

[1] Scoring indicates the extent of evidence available, not methodological quality of assessments or adequacy of measurement properties. Scoring is based on the assessments summarized in Table S9 (Additional File 7).

[2] Key ✓ - some description OR some assessment (e.g., brief description of construct, implicit theory, single administration), ✓✓ - comprehensive description (e.g., explicit construct definition) OR multiple assessments under relevant conditions, X - not assessed or insufficient detail, ? – unclear, NA – not applicable (e.g., a priori hypotheses not applicable if no hypotheses were tested

References

1. Alemi F, Safaie FK, Neuhauser D: **A survey of 92 quality improvement projects.** *Jt Comm J Qual Improv* 2001, **27:**619-632.
2. Anderson N, West M: **Measuring climate for work group innovation: Development and validation of the Team Climate Inventory.** *J Organ Behav* 1998, **19:**235-258.
3. Hearnshaw H, Reddish S, Carlyle D, Baker R, Robertson N: **Introducing a quality improvement programme to primary healthcare teams.** *Qual Health Care* 1998, **7:**200-208.
4. Lukas CV, Mohr DC, Meterko M: **Team effectiveness and organizational context in the implementation of a clinical innovation.** *Qual Manag Health Care* 2009, **18:**25-39.
5. Shortell SM, Rousseau DM, Gillies RR, Devers KJ, Simons TL: **Organizational assessment in intensive care units (ICUs): construct development, reliability, and validity of the ICU nurse-physician questionnaire.** *Med Care* 1991, **29:**709-726.
6. Shortell SM, Marsteller JA, Lin M, Pearson ML, Wu SY, Mendel P, Cretin S, Rosen M: **The role of perceived team effectiveness in improving chronic illness care.** *Med Care* 2004, **42:**1040-1048.
7. Adler-Milstein J, Neal K, Howell MD: **Residents' and nurses' perceptions of team function in the medical intensive care unit.** *J Crit Care* 2011, **26:**104.e107-115.
8. Baker DP, Amodeo AM, Krokos KJ, Slonim A, Herrera H: **Assessing teamwork attitudes in healthcare: development of the TeamSTEPPS teamwork attitudes questionnaire.** *Qual Saf Health Care* 2010, **19:**e49.
9. Caldwell DF, O'Reilly CA, III: **The determinants of team-based innovation in organizations. The role of social influence.** *Small Gr Res* 2003, **34:**497-517.
10. Campion MA, Medsker GJ, Higgs AC: **Relations between work group characteristics and effectiveness: Implications for designing effective work groups.** *Pers Psychol* 1993, **46:**823-850.
11. Costa AC, Anderson N: **Measuring trust in teams: Development and validation of a multifaceted measure of formative and reflective indicators of team trust.** *Eur J Work Org Pscychol* 2011, **20:**119-154.
12. Dobson RT, Stevenson K, Busch A, Scott DJ, Henry C, Wall PA: **A quality improvement activity to promote interprofessional collaboration among health professions students.** *Am J Pharm Educ* 2009, **73:**64.
13. Duckers MLA, Wagner C, Groenewegen PP: **Developing and testing an instrument to measure the presence of conditions for successful implementation of quality improvement collaboratives.** *BMC Health Serv Res* 2008, **8:**172.

14. Edmondson A: **Psychological safety and learning behavior in work teams.** *Adm Sci Q* 1999, **44:**350-383.
15. Fulmer T, Hyer K, Flaherty E, Mezey M, Whitelaw N, Jacobs MO, Luchi R, Hansen JC, Evans DA, Cassel C, et al: **Geriatric interdisciplinary team training program: evaluation results.** *J Aging Health* 2005, **17:**443-470.
16. Gould BE, Grey MR, Huntington CG, Gruman C, Rosen JH, Storey E, Abrahamson L, Conaty AM, Curry L, Ferreira M, et al: **Improving patient care outcomes by teaching quality improvement to medical students in community-based practices.** *Acad Med* 2002, **77:**1011-1018.
17. Heinemann G, Schmitt MH, Farrell M, Brallier S: **Development of an attitudes toward health care teams scale.** *Eval Health Prof* 1999, **22:**123-142.
18. Irvine DM, Leatt P, Evans MG, Baker GR: **The behavioural outcomes of quality improvement teams: the role of team success and team identification.** *Health Serv Manage Res* 2000, **13:**78-89.
19. Irvine Doran DM, Baker GR, Murray M, Bohnen J, Zahn C, Sidani S, Carryer J: **Achieving clinical improvement: an interdisciplinary intervention.** *Health Care Manage Rev* 2002, **27:**42-56.
20. Lemieux-Charles L, Murray M, Baker G, Barnsley J, Tasa K, Ibrahim S: **The effects of quality improvement practices on team effectiveness: a mediational model.** *J Organ Behav* 2002, **23:**533-553.
21. Lewis K: **Measuring transactive memory systems in the field: Scale development and validation.** *J Appl Psychol* 2003, **88:**587-604.
22. Lichtenstein R, Alexander JA, Jinnett K, Ullman E: **Embedded intergroup relations in interdisciplinary teams: Effects on perceptions of level of team integration.** *J Appl Behav Sci* 1997, **33:**413-434.
23. Lubomski LH, Marsteller JA, Hsu YJ, Goeschel CA, Holzmueller CG, Pronovost PJ: **The team checkup tool: evaluating QI team activities and giving feedback to senior leaders.** *Jt Comm J Qual Patient Saf* 2008, **34:**619-623, 561.
24. Mills PD, Weeks WB: **Characteristics of successful quality improvement teams: lessons from five collaborative projects in the VHA.** *Jt Comm J Qual Saf* 2004, **30:**152-162.
25. Millward LJ, Jeffries N: **The team survey: a tool for health care team development.** *J Adv Nurs* 2001, **35:**276-287.
26. Schouten L, Grol R, Hulscher M: **Factors influencing success in quality improvement collaboratives: development and psychometric testing of an instrument.** *Implement Sci* 2010, **5:**84.

27. Thylefors I, Persson O, Hellstrom D: **Team types, perceived efficiency and team climate in Swedish cross-professional teamwork.** *J Interprof Care* 2005, **19:**102-114.

28. Allen JM, Schumacker RE: **Team assessment utilizing a many-facet Rasch model.** *J Outcome Meas* 1998, **2:**142-158.

29. Barczak G, Lassk F, Mulki J: **Antecedents of team creativity: An examination of team emotional intelligence, team trust and collaborative culture.** *Creat Innov Man* 2010, **19:**332-345.

30. Brav A, Andersson K, Lantz A: **Group initiative and self-organizational activities in industrial work groups.** *Eur J Work Org Pscychol* 2009, **18:**347-377.

31. Bunderson J, Boumgarden P: **Structure and learning in self-managed teams: Why "bureaucratic" teams can be better learners.** *Organ Sci* 2010, **21:**609-624.

32. Carless SA, Wearing AJ, Mann L: **A short measure of transformational leadership.** *J Bus Psychol* 2000, **14:**389-405.

33. Driskell JE, Salas E, Hughes S: **Collective orientation and team performance: development of an individual differences measure.** *Hum Factors* 2010, **52:**316-328.

34. Eby LT, Dobbins GH: **Collectivistic orientation in teams: An individual and group-level analysis.** *J Organ Behav* 1997, **18:**275-295.

35. Kirkman BL, Rosen B: **Beyond self-management: antecedents and consequences of team empowerment.** *Acad Manage J* 1999, **42:**58-74.

36. Langfred CW: **Autonomy and performance in teams: the multilevel moderating effect of task interdependence.** *J Manage* 2005, **31:**513-529.

37. Manz CC, Sims HP: **Leading workers to lead themselves: The external leadership of self-managing work teams.** *Adm Sci Q* 1987, **32:**106-129.

38. Mathieu JE, Gilson LL, Ruddy TM: **Empowerment and team effectiveness: an empirical test of an integrated model.** *J Appl Psychol* 2006, **91:**97-108.

39. Shaw JD, Duffy MK, Stark EM: **Interdependence and preference for group work: Main and congruence effects on the satisfaction and performance of group members.** *J Manage* 2000, **26:**259-279.

40. Staples D, Webster J: **Exploring the effects of trust, task interdependence and virtualness on knowledge sharing in teams.** *Inform Syst J* 2008, **18:**617-640.

41. van den Bossche P, Gijselaers WH, Segers M, Kirschner PA: **Social and Cognitive Factors Driving Teamwork in Collaborative Learning Environments: Team Learning Beliefs and Behaviors.** *Small Gr Res* 2006, **37:**490-521.

42. van der Vegt G, Emans B, van de Vliert E: **Motivating effects of task and outcome interdependence in work teams.** *Group Organ Manage* 1998, **23:**124-143.
43. Wageman R, Hackman J, Lehman E: **Team Diagnostic Survey: Development of an Instrument.** *J Appl Behav Sci* 2005, **41:**373-398.
44. Deeter-Schmelz DR, Ramsey RP: **An investigation of team information processing in service teams: exploring the link between teams and customers.** *J Acad Market Sci* 2003, **31:**409-424.
45. Thompson BM, Levine RE, Kennedy F, Naik AD, Foldes CA, Coverdale JH, Kelly PA, Parmelee D, Richards BF, Haidet P: **Evaluating the quality of learning-team processes in medical education: development and validation of a new measure.** *Acad Med* 2009, **84:**S124-127.
46. Vinokur-Kaplan D: **Treatment teams that work (and those that don't): An application of Hackman's group effectiveness model to interdisciplinary teams in psychiatric hospitals.** *J Appl Behav Sci* 1995, **31:**303-327.
47. Wilkens R, London M: **Relationships between climate, process, and performance in continuous quality improvement groups.** *J Vocat Behav* 2006, **69:**510-523.
48. Arnold JA, Arad S, Rhoades JA, Drasgow F: **The Empowering Leadership Questionnaire: the construction and validation of a new scale for measuring leader behaviors.** *J Organ Behav* 2000, **21:**249-269.
49. Bunderson JS, Sutcliffe KM: **Management team learning orientation and business unit performance.** *J Appl Psychol* 2003, **88:**552-560.
50. Coyle-Shapiro JAM, Morrow PC: **The role of individual differences in employee adoption of TQM orientation.** *J Vocat Behav* 2003, **62:**320-340.
51. de Jong B, Elfring T: **How does trust affect the performance of ongoing teams? The mediating role of reflexivity, monitoring, and effort.** *Acad Manage J* 2010, **53:**535-549.
52. Hiller NJ, Day DV, Vance RJ: **Collective enactment of leadership roles and team effectiveness: A field study.** *Leadership Quart* 2006, **17:**387-397.
53. Hoegl M, Gemuenden HG: **Teamwork quality and the success of innovative projects: A theoretical concept and empirical evidence.** *Organ Sci* 2001, **12:**435-449.
54. Janssen O, Van de Vliert E, Veenstra C: **How task and person conflict shape the role of positive interdependence in management teams.** *J Manage* 1999, **25:**117-142.
55. Jehn KA, Greer L, Levine S, Szulanski G: **The effects of conflict types, dimensions, and emergent states on group outcomes.** *Group Decis Negot* 2008, **17:**465–495.
56. Kuhn T, Poole MS: **Do conflict management styles affect group decision making? Evidence from a longitudinal field study.** *Hum Commun Res* 2000, **26:**558-590.

57. Savelsbergh CM, van der Heijden BI, Poell RF: **The development and empirical validation of a multidimensional measurement instrument for team learning behaviors.** *Small Gr Res* 2009, **40:**578-607.

58. Schippers MC, Den Hartog DN, Koopman PL: **Reflexivity in teams: A measure and correlates.** *Appl Psychol Int Rev* 2007, **56:**189-211.

59. Seers A: **Team-member exchange quality: A new construct for role-making research.** *Organ Behav Hum Decis Process* 1989, **43:**118-135.

60. Tjosvold D, Wedley WC, Field RH: **Constructive controversy, the Vroom-Yetton model, and managerial decision-making.** *J Occup Behav* 1986, **7:**125-138.

61. West MA, Borrill CS, Dawson JF, Brodbeck F, Shapiro DA, Haward B: **Leadership clarity and team innovation in health care.** *Leadership Quart* 2003, **14:**393-410.

62. Lichtenstein R, Alexander J, Jinnett A, Ullman E: **Embedded intergroup relations in interdisciplinary teams: Effects on perceptions of level of team integration.** *J Appl Behav Sci* 1997, **33:**413-434.

63. Lichtenstein R, Alexander JA, McCarthy JF, Wells R: **Status differences in cross-functional teams: effects on individual member participation, job satisfaction, and intent to quit.** *J Health Soc Behav* 2004, **45:**322-335.

64. Thylefors I, Persson O, Hellstrom D: **Team types, perceived efficiency and team climate in Swedish cross-professional teamwork.** *J Interprof Care* 2005, **19:**102-114.

65. Tucker AL, Nembhard IM, Edmondson AC: **Implementing New Practices: An Empirical Study of Organizational Learning in Hospital Intensive Care Units.** *Manage Sci* 2007, **53:**894-907.

66. Bishop JW, Scott K, Goldsby MG, Cropanzano R: **A construct validity study of commitment and perceived support variables: A multifoci approach across different team environments.** *Group Organ Manage* 2005, **30:**153-180.

67. Carless SA, De Paola C: **The measurement of cohesion in work teams.** *Small Gr Res* 2000, **31:**71-88.

68. Guzzo RA, Yost PR, Campbell RJ, Shea GP: **Potency in groups: Articulating a construct.** *Br J Soc Psychol* 1993, **32:**87-106.

69. Janssen O, Huang X: **Us and me: Team identification and individual differentiation as complementary drivers of team members' citizenship and creative behaviors.** *J Manage* 2008, **34:**69-88.
