# Supplementary material for: Measuring team factors thought to influence the success of quality improvement in primary care: a systematic review of instruments
Source: Implement Sci. 2013 Feb 14;8:20. doi: 10.1186/1748-5908-8-20 (PMC3602018; doi:10.1186/1748-5908-8-20)
Supplement: Additional file 5 — Table summarizing the characteristics of instruments included for review of measurement properties (Stage four) (Table S7). [file 1748-5908-8-20-S5.pdf]

Additional file 5

Table S7: Characteristics of instruments included for review of measurement properties (Stage four)

**Instrument**<br>(name, index, references for main reports)	**Main purpose**<br>(measurement aim and context of use; examples of use in health care)	**Description of content**<br>(dimensions, sub-dimensions; conceptual/theoretical basis)	**Items & response scale**	**Study design(s)**	**Comments**<br><br>[main domain(s) measured]
Primary care settings					
Team climate for innovation inventory (TCI)	Discriminative/predictive/outcome: to measure team climate as a predictor of innovation in any setting, including primary care. (UK)	Team climate for innovation: vision (clarity, visionary nature, attainability, shared vision and goals), participation safety (active participation; atmosphere of non-threatening trust and support), task orientation (concern for quality, evidenced by monitoring, evaluating and modifying performance; reflection; feedback; exploring opposing views), support for innovation (expectation and practical support for introducing new and improved ways of working)	38 items, 4 scales; 5 or 7 point response scale.<br><br>14 item short form [1]	Instrument development	A very widely used measure of teamwork in health care, with initial development and multiple examples of use in primary care.
Anderson 1998 [2-3]	Other examples: Short form Kivimaki 1999 [1]. (Finland) Use in primary care includes [4-11]). (UK, Netherlands, Australia)	Based on West and Anderson's four factor model of facet-specific climate for innovation [12-13].			[EMERGENT, PROCESS]
Team culture questionnaire	Diagnostic: pre-intervention measure to help primary care teams identify aspects of their practice culture that are potential targets for change using a total quality management intervention. (UK)	Team culture (perceived importance of …): innovation seeking (new ways of working, problem solving); planning (prior to taking action, long term planning); control and loyalty (managerial control, employee loyalty); cooperation (working collaboratively); communication and information (open communication, sharing ideas); valuing staff (development of staff). Intended to capture beliefs at the practice level, not individual beliefs.	27 items; 7 point Likert scale.	Observational, descriptive.	Reported as a measure of team culture, however items refer to organisational culture. The instrument is included because of its use in a QI study in primary care, however very few of the items relate to teamwork.
Hearnshaw 1998 [14]	Other examples: none identified	Dimensions are based on team characteristics reported to influence introduction of QI in primary care (selected studies [15-17]).			[ANTECEDENT]
Working as a team survey	Predictive: Post-intervention measurement of team factors as predictors of implementation of a clinical innovation in primary and specialist care clinics participating in a QI initiative [18-19]. (USA)	Team problem recognition, team knowledge and skills, team functioning, personal leadership support.	28 items, 4 scales; 5 point Likert scale.	Observational, analytical - longitudinal	The problem recognition and leadership scales include items specific to the QI intervention in this study. These could be reworded as generic items.
Lukas 2009 [18]	Other examples: Selected items used to operationalise theory of planned behaviour (TPB) constructs as predictors of participation in clinic redesign [20]. (USA)	Items derived from two existing instruments [21-22]. The authors' reference selected papers on team functioning and learning, but do not provide an explicit theoretical basis for the instrument.			[PROCESS, CONTEXT]
Organizational assessment measure	Predictive/outcome: To measure managerial and organisational processes as i) potential predictors of healthcare quality and efficiency and ii) outcomes of interventions aiming to improve organisational factors in intensive care units (ICU). (USA)	Managerial and organisational processes: teamwork and leadership (emphasis on excellence, clear goals, responsive to needs, understanding of staff concerns), problem-solving/managing disagreements (collaborative problem-solving, conflict management approach), relationships and communication (openness, accuracy, timeliness, understanding, satisfaction).<br>Authority and perceived effectiveness scales relate wholly to clinical context, so excluded from content analysis because they are unsuitable for CQI teams.	15 items, 2 scales (short version), 5 point Likert scale.	Instrument development	Not explicitly a measure of teamwork; however, the scales have been widely used and adapted as measures of teamwork in health care. Some scales are unsuitable for CQI because of focus on clinical care.
Shortell, 1991 [23-24]	Other examples: Version for primary care [24], used as an outcome measure in a randomized trial of a guideline implementation strategy [25-26]. Adapted to measure teamwork in long term care settings [27]. (USA)	Based on theoretical framework derived from organisational behaviour literature (multiple references for each construct).			[CONTEXT, PROCESS, EMERGENT, OUTCOME]

**Instrument** (name, index, references for main reports)	**Main purpose** (measurement aim and context of use; examples of use in health care)	**Description of content** (dimensions, sub-dimensions; conceptual/theoretical basis)	**Items & response scale**	**Study design(s)**	**Comments** [main domain(s) measured]
Perceived team effectiveness survey	Predictive: Post-intervention measurement of team effectiveness as a predictor of changes to chronic illness care in teams participating in a national evaluation of QICs. (USA)	Perceived team effectiveness: overall perceived effectiveness, perceived team skill, perceived participation and goal agreement, perceived organisational support.	25 items, 4 scales; 7 point Likert scale for most items.	Observational – analytical, longitudinal	Based on Lemieux-Charles 2002 measure of team effectiveness [21], this adaptation is substantive enough to warrant inclusion as a separate instrument.
Shortell 2004 [22, 28]	Other examples: Used to assess convergent validity of the Team Check-up Tool (TCT) in teams participating in a QI intervention in intensive care units [29]. (USA)	Intended to measure “aspects of team effectiveness potentially relevant to quality improvement work” (p1041) as identified in Lemieux-Charles’ model [21] and two other sources [30-31].			[CONTEXT, PROCESS, EMERGENT, OUTCOME]
Other health care settings					
Group innovation inventory	Predictive: to measure work group climate for innovation as a predictor of innovation in high technology organisations [32]. Testing of measurement properties in teams participating in a QIC to improve home-based care and development of short form [33]. (USA, Netherlands)	Group normative climate supporting innovation: support for creativity and risk taking, teamwork, speed of action, tolerance of mistakes.<br><br>Conceptually similar to the team climate inventory [2], but intended to distinguish between norms that support creativity and norms that support implementation.	25 items, 4 scales; 5 point Likert scale.<br><br>19 item short form [33]	Instrument development	Conceptually similar to team climate inventory, but with few examples of use. Single use in QIC in health care [33].
Caldwell 2003 [32-33]	Other examples: none identified	Based on selected empirical and theoretical work on work group climate for innovation, focusing in a model developed by the authors.			[EMERGENT]
Work group characteristics measure	Predictive: to measure work group characteristics predictive of group effectiveness in any organisational setting [34-35]. (USA)	Work group characteristics: Job design (self management, participation, task variety, task significance, task identity), interdependence (task, goal, interdependent feedback and rewards), team composition (heterogeneity, flexibility, relative size, preference for group work), context (training, managerial support, communication/cooperation between groups), process (potency, social support, workload sharing, communication/cooperation within groups)	54 items, 19 scales; 5 point Likert scale.	Instrument development	Scales widely used and adapted, with some examples in health care. Comprehensive measure of enabling conditions.
Campion 1993 [34]	Other examples: Job design scales used to investigate relationship between work design, team process, and care outcomes in multidisciplinary healthcare teams [36]. (USA)	Based on a conceptual framework for work group characteristics developed from a literature review (main sources were [37-40]).			[ENABLING CONDITIONS, PROCESS]
Team trust	Predictor: to measure trust as a mediator of teamwork behaviour and performance. Initial testing with hospital professionals. (Netherlands)	Intra-team trust as measured by four indicators: propensity to trust (willingness to trust others); perceived trustworthiness (belief that others will behave in accordance with commitments, are honest in negotiating commitments, and do not take advantage); cooperative behaviours (willingness to be vulnerable to others and cooperation), and monitoring behaviours (perceived need to monitor and check others).	21 items, 4 scales; 7 point Likert scale.	Instrument development	Construct is narrow but important, and the strong theoretical basis and ability to use individual scales makes this a potentially relevant measure.
Costa 2011 [41]	Other examples: Earlier versions of the instruments used to examine the association between trust and performance in social care teams [42-43] (Netherlands)	Based on theoretical and empirical research on trust.			[EMERGENT]
Knowledge, attitudes and beliefs relating to inter-professional teams	Outcome: to measure change in knowledge, attitudes and beliefs relating to inter-professional teams among health professional students participating in a CQI project. (Canada)	Individual knowledge, attitudes and beliefs relating to working in inter-professional teams (comfort, ease and efficacy for inter-professional team work; understanding of own and other’s roles; beliefs about process and outcomes)	16 items; 7 point scale with bipolar anchor statements	Pre- and post intervention evaluation	A measure of attitudes toward working in an inter-professional team that is not specific to provision of clinical care, and therefore relevant to CQI.
Dobson 2009a [44-45]	Other examples: none identified	Based on a model of inter-professional learning developed from narrative literature			[ANTECEDENT]

Instrument (name, index, references for main reports)	Main purpose (measurement aim and context of use; examples of use in health care)	Description of content (dimensions, sub-dimensions; conceptual/theoretical basis)	Items & response scale	Study design(s)	Comments [main domain(s) measured]
		review of teamwork literature [45-46].			
Group process questionnaire	Outcome: to measure perceptions of group process among health professional students participating in a CQI project. (Canada)	Group process: trust, support for team members, open communication, clear objectives to which team members were committed, performance, conflict management, consideration and use of members ideas and abilities, appropriateness of QI methods.	9 items; 7 point scale with bipolar anchor statements	Pre- and post intervention evaluation	Although described as a measure of process, half the items refer to emergent states. More suitable as a short measure of climate, than team process.
Dobson 2009b [44]	Other examples: none identified	Conceptual basis of measure not explicitly reported.			[EMERGENT, PROCESS]
Team learning behaviour	Predictive: to examine whether team beliefs (psychological safety, efficacy), and team learning behaviours are predictive of team performance, and the relationship between these constructs. (USA)	Team learning behaviours ("… reflection and action, characterized by asking questions, seeking feedback, experimenting, reflecting on results, and discussing errors or unexpected outcomes" [47] p353).	7 items; 7 point Likert scale	Observational, analytical - longitudinal	Developed outside health care but generic and with use in health care.
Edmondson 1999a [47]	Other examples: Learning behaviours scale used to explore learning in hospitals [48]. (USA, Australia)	Based on a model of team learning developed by the author, which draws on organisational learning theory (for examples, see [49-51]).			[PROCESS, EMERGENT]
Team shared beliefs	Predictive: to examine whether team beliefs (psychological safety, efficacy), mediate the effect of team learning behaviours on team performance. (USA)	Shared team beliefs: team efficacy (belief in the team's competence), psychological safety (belief that the team is safe for interpersonal risk taking).	10 items, 2 scales; 7 point Likert scale	Observational, analytical - longitudinal	Developed outside health care but generic and with use in health care. The scales can be used separately.
Edmondson 1999b [47]	Other examples: Psychological safety scale used as a predictor of engagement in QI work in QIC teams [52] and nurses [53], and of performance in CQI teams [54].	Based on a model of team learning developed by the author, which draws on organisational learning theory (for examples, see [49-51]).			[PROCESS, EMERGENT]
Teamwork context and internal team management	Outcome/predictive: to measure teamwork context and internal team management as mediators of the effect of QI practices on perceived effectiveness in hospital CQI teams. (Canada)	Supportive context for teamwork (level and type of support teams receive, including cross-unit cooperation, collaboration and communication). Internal team management: process strategies (leader behaviours in managing team process, effort), decision-making (participation and contributions from all members, considering different ideas and perspectives, applying knowledge and skills), norms (norms of behaviour including strength of goal agreement and commitment).	23 items, 4 scales; 7 point Likert scale.	Observational, analytical – cross sectional	One of the few instruments used to test a model of the relationship between use of QI practices, teamwork, and team effectiveness. Adaptations reported separately [18, 22].
Lemieux-Charles 2002a [21]	Other examples: scales adapted for use in QI teams in primary care [18] and QIC teams [22]. (USA)	Model of QI team effectiveness developed by the authors, primarily based on established models of team effectiveness [30, 55-56].			[PROCESS, EMERGENT, CONTEXT]
Perceived team effectiveness	Outcome: to determine if use of QI practices, teamwork context and internal team management are predictive of perceived effectiveness in hospital CQI teams. (Canada)	Perceived team effectiveness: overall task performance met their expectations, satisfaction with experience as a team member, felt positive about their experience, willingness to work on a similar team.	4 items; 5 point Likert scale.	Observational, analytical – cross sectional	Hackman's conceptualisation of team effectiveness is the most widely used. This measure is included because of use in CQI teams in health care.
Lemieux-Charles 2002b [21]	Other examples: scale adapted by for use in QIC teams [22]. (USA	Based on Hackman's conceptualisation of team effectiveness [39].			[OUTCOME]
Transactive memory system scale	Predictive: to explore the relation between a team's ability to utilise and integrate collective knowledge and expertise (transactive memory systems), and team performance. (USA)	Transactive memory systems: specialisation (recognition of differentiated, specialised knowledge of team members), credibility (willingness to rely on others for task critical information), coordination (an indicator of transactive memory).	15 items, 3 scales; 5 point Likert scale.	Instrument development	Transactive memory systems are important for teams that need to use and integrate knowledge and expertise that is distributed among team members. The measure is

Instrument (name, index, references for main reports)	Main purpose (measurement aim and context of use; examples of use in health care)	Description of content (dimensions, sub-dimensions; conceptual/theoretical basis)	Items & response scale	Study design(s)	Comments [main domain(s) measured]
					particularly relevant for cross-functional project teams [57].
Lewis 2003 [57]	Other examples: Used to examine the relationship between transactive memory systems, perceived team effectiveness and work attitudes in anaesthesia teams [58]. (France)	Based on theories arising from knowledge management, primarily [59-60].			[EMERGENT]
Perceived team integration	Outcome: dependent variable in study examining the relationship between team member diversity (status and other differences) and integration in clinical teams in hospitals. (USA)	Perceived team integration (how well individuals function within a team and how well members relate to each other): participation (frequency of interaction of members of the group and the number of activities in which they participate together), role clarity, individual assessment of team functioning (cooperation, coordination, decision quality, quality of outcomes).	19 items, 3 scales; 7 point Likert scale.	Observational, analytical – cross sectional	Scales have been used in QI studies. Role clarity and team functioning subscales refer to clinical care; would require minor rewording for QI teams.
Lichtenstein 1997 [61]	Other examples: predictor of patient functional status in study of interdisciplinary hospital-based psychiatric care (2 scales) [62-63]; descriptive measure of functioning in QIC teams in primary care (1 scale) [64]. (USA)	Based on social identification theory and embedded intergroup relations theory [65-67].			[PROCESS, OUTCOME, EMERGENT]
Team check-up tool (TCT)	Diagnostic/predictive: to track the progress of team-based QI intervention in the ICU and identify contextual variables linked to progress and outcomes. The initial purpose of the tool was to monitor support and communication between hospital leaders and QI teams [68]. (USA)	Context and progress of team-based QI intervention: intervention activities; perceived unit-level intervention-related behaviour; implementation processes and context (e.g. leadership support, resources); perceived barriers to team progress (knowledge of evidence and QI skills, goal agreement, time, buy-in from other staff, leadership support, autonomy/authority, ability to work as a team). Respondents reporting an inability to work together complete five teamwork items (participation, valuing contributions, team cohesion, conflict, conflict resolution).	31-36 items; response scales varied, 5 point for barriers.	Instrument development	The measure focuses on context. Of 31 items, five relate broadly to team traits or function. Teams reporting an inability to work together complete an additional five items about team function.
Lubomski 2008 [29, 68]	Other examples: used in evaluation of team-based QI interventions in the ICU (multiple studies from same authors).	A conceptual framework for the tool is reported, but the theoretical basis of the framework is not described [29].			[CONTEXT, PROCESS, EMERGENT]
Team characteristics questionnaire	Predictive: To identify characteristics of QI teams and perceived organisational support that predict successful outcomes for QIC teams. (USA)	Team characteristics and organisation support predictive of QI success: team-work skills; organizational leadership; prior experience with quality improvement and measurement; useful information systems.	20 items, 4 scales; 7 point Likert scale.	Pre- and post intervention evaluation	Designed for pre- and post-intervention measurement - 15 of the items are included at both time points and five items are specific to either pre- or post-intervention measurement.
Mills 2004 [69-70]	Other examples: Used in multiple evaluations of QICs in the Veterans Health Administration, mainly in hospital settings [70-73]. Mills and colleagues report analysis of combined data from five of these studies [69]. (USA)	Based on review of selected literature on characteristics of successful change teams and microsystems [74-77]			[ENABLING CONDITIONS, PROCESS, EMERGENT, OUTCOMES]
The team survey	Predictive/outcome: To measure cognitive and motivational causes of effective teamwork in health care and enable evaluation of interventions aiming to change these factors. (UK)	Cognitive and motivational causes of team effectiveness: team potency, team identification, shared mental models, team meta-cognitions (e.g. goal clarity, cohesion) communication, team perspectives, valuing others	39 items, 7 scales; 5 point Likert scale.	Instrument development	First report for instrument, adapted for use with healthcare teams and based on prior work with teams in other contexts.
Millward 2001 [78]	Other examples: none identified	Based on the cognitive-motivational model of team effectiveness (CoMMTE) developed by the authors (unpublished report), and theories of team potency, shared mental models and team identification.			[PROCESS, EMERGENT]

**Instrument** (name, index, references for main reports)	**Main purpose** (measurement aim and context of use; examples of use in health care)	**Description of content** (dimensions, sub-dimensions; conceptual/theoretical basis)	**Items & response scale**	**Study design(s)**	**Comments** [main domain(s) measured]
Factors influencing success in a QIC (teamwork scale)	Predictive/diagnostic: To measure potential determinants of QIC success. The authors suggest retrospective use in QIC evaluations or prospective use by change agents as a checklist to optimise chances of success. (Netherlands)	Effective multidisciplinary teamwork: clearly defined roles, management support, preparation and organisation of collaborative time, use of the improvement model.	18 items, 5 point Likert scale	Instrument development	Scale is one of three from the Schouten 2010 instrument. It is suitable for use as a stand alone scale in an evaluation of team-based QI.
Schouten, 2010 [79]	Other examples: none identified	Based on findings from a systematic search for papers on QIC theory (e.g. papers [80-82])			[PROCESS, EMERGENT, CONTEXT]
Team performance survey (TPS)	Discriminative: to measure differences between groups in the quality of team or small-group interactions among medical students. (USA)	Quality of team member interaction: items encompass participation; eliciting, discussing and incorporating different points of view; paying attention to others; problem solving and decision making; providing feedback on individual performance.	18 items; 7 point Likert scale.	Instrument development	Instrument measures collaborative behaviours, and was originally designed for medical education using team-based learning methods.
Thompson 2009 [83]	Other examples: none identified	Based on review of literature on small group learning (two references provided), but the conceptual basis for items and construct definitions is not explicitly reported.			[PROCESS]
CQI team climate and interpersonal relationships	Predictive: To examine the relationship between team climate, interpersonal relationships and perceived team performance (self- and assessor-rated) in hospital CQI teams. (USA)	Team climate and interpersonal relationships: group climate (self-disclosure; group learning orientation; psychological safety), interpersonal relationships (perceptions of feedback giving and seeking; task and relationship conflict)	33 items, 7 scales; 7 point Likert scale.	Observational, analytical – cross sectional	Many of the scales are adapted from commonly used scales measuring team constructs.
Wilkens 2006 [54]	Other examples: none identified	Overall, based on theories related to CQI and group process (e.g. [84-85]). Each of the scales comprising the measure are based on related theoretical work (e.g. Jehn's scale based on theories of intra-group conflict [86])			[PROCESS, EMERGENT]
Non health care					
Empowering leadership questionnaire (ELQ)	Diagnostic/predictive: to measure empowering leadership behaviours in teams in any setting. (USA)	Empowering leadership: coaching behaviours (providing feedback, encouraging and teaching teamwork behaviours), informing (explaining organisational goals, decisions, policies and expectations in relation to the team's work), leading by example (setting and demonstrating high standards for own performance), showing concern/interacting with the team, participative decision-making (leader's decision-making style is participative)	38 items, 5 scales; 5point Likert scale.	Instrument development	An instrument suitable for measuring leader behaviours that empower teams. Scales can be used individually.
Arnold 2000 [87]	Other examples: none identified in health care.	Based on a model of leader behaviours that support team empowerment developed by the authors from analysis of key informant interviews and related theory (e.g. [88-90]).			[LEADER]
Team learning orientation	Outcome/predictive: to measure learning orientation as an outcome of team structure and process [91], and as a predictor of objectively measured team performance [92]. (USA)	Team learning orientation: extent to which a team emphasizes learning goals (development of skill, knowledge, and competence) over performance goals (demonstrating competence and avoiding failure) (p552-3)	5 items; 7 point Likert scale.	Observational, analytical – longitudinal	Measures an attitudinal construct that may predict whether teams are receptive to adopting learning behaviours, which are an important component of CQI methods.
Bunderson 2003 [91-92]	Other examples: none identified in health care.	Based on theories of group/collective goal orientation and organisational learning (multiple source references provided).			[EMERGENT]
Team structure	Predictive: antecedent variable in a model testing the association between team structure, team process (information sharing), and team learning orientation [91]. (USA)	Team structure: extent to which team has an elaborate division of labour based on specialisation (horizontal division) and hierarchy (vertical division), and the extent to which there are clear procedures for coordinating and prioritising work (formalization).	5 items; 7 point Likert scale.	Observational, analytical – cross sectional	Although measuring a narrow construct, this is one of few instruments measuring team structure with items relevant for CQI teams.
Bunderson 2010a [92]	Other examples: none identified in health care.	Based on conceptualisations of organisational structure from sociological and			[TEAM DESIGN]

Instrument (name, index, references for main reports)	Main purpose (measurement aim and context of use; examples of use in health care)	Description of content (dimensions, sub-dimensions; conceptual/theoretical basis)	Items & response scale	Study design(s)	Comments [main domain(s) measured]
		organisational theory (e.g. [93]).			
Teamwork and trust in colleagues	Outcome: Dependent variable in study in manufacturing sector exploring determinants of individual adoption of a TQM orientation. (UK)	Teamwork (extent of teamwork based on: team spirit and cooperation, willingness to support and encourage other team members, value placed on team performance), trust (competence, ability to rely on workmates)	12 items, 2 scales; 7 point Likert scale	Observational – analytical, longitudinal	Developed for CQI teams in the manufacturing sector, however item wording is generic and suitable for any context.
Coyle-Shapiro, 2003 [94]	Other examples: none identified in health care.	Based on a review of TQM literature (selected references cited).			[EMERGENT]
Collective orientation scale	Predictor: to measure collective orientation as a predictor of team performance, in any team. (USA)	Collective orientation: affiliation (preference for working with others versus. working alone), dominance (self-interest, dominance, and control versus other-interest and cooperation)	15 items; 5 point Likert scale.	Instrument development	Scales are conceptually relevant, measuring preferences for teamwork over individual work, and have a sound theoretical basis.
Driskell 2010 [95]	Other examples: Selected items used to develop a new measure of teamwork among emergency medical technicians [96], but no examples of use of instrument or scales.	Based on theory and empirical research on individualism and collectivism (e.g. [97])			[ANTECEDENT]
Team context	Predictive: to examine whether team structures are predictive of shared beliefs, learning behaviour and team performance. (USA)	Supportiveness of organisational context (information, expert assistance and training, rewards or recognition), team leader coaching, task design (significance, feedback), clear direction, team composition (capabilities).	14 items, 5 scales; 7 point Likert scale	Observational, analytical - longitudinal	Includes scales from other sources (e.g [55]). Not all scales were included in model tested by the authors.
Edmondson 1999c [47]	Other examples: none identified	Based on a model of team learning developed by the author, which draws on organisational learning theory (for examples, see [49-51]).			[ENABLING CONDITIONS, EMERGENT]
Collective leadership scales	Predictive: to measure collective leadership roles and behaviours as mediators of team performance. (USA)	Collective leadership behaviours: related to tasks (frequency with which team members share in planning and organising the team's work; problem solving), related to relationships (providing support and consideration for team members; development and mentoring of team members).	25 items,4 scales; 7 point Likert scale.	Instrument development	Scales are relevant to measuring how team members share responsibility for core tasks and can be used individually.
Hiller 2006 [98]	Other examples: none identified in health care.	Based on theories of collective leadership, focusing on behaviours included in the Managerial Practices Survey [99]			[PROCESS, LEADER]
Teamwork quality (TWQ) scales	Predictive: to measure teamwork quality as a predictor of the success of innovation projects, focusing in this study on software development teams. (Germany)	Team collaborative processes: communication (frequent, informal, direct, open), coordination (individual efforts structured and synchronised), balance of member contributions (expertise used to full potential), mutual support (help and support in carrying out tasks), effort (exerted on team tasks), cohesion (motivation to maintain the team).	38 items, 6 scales; 5 point Likert scale.	Instrument development	Measures multiple dimensions of team process and emergent states, focusing on project teams. Content and item wording are suitable for CQI teams in primary care.
Hoegl 2001 [100]	Other examples: multiple studies by the developers, but none identified in health care.	Based on selected literature on teamwork process, focussing on collaborative behaviours that support team innovation (e.g. [39-40, 101-103])			[PROCESS, EMERGENT]
Decision making processes and quality scales	Predictive/outcome: to test a model of the relationship between team interdependence, conflict management processes and perceived decision making quality in management teams. (Netherlands)	Conflict behaviours during decision making processes: use of integrative behaviours (information exchange, search for underlying objectives, redefinition of initially incompatible standpoints, integration of different perspectives into high-quality decisions), use of distributive behaviour (suppressing or trivialising issues to prevent controversy, evading dialectical interactions with opponents). Decision quality (quality compared to initial proposals or individual ideas; best the team was capable of).	16 items measuring process, 2 scales; 3 items measuring satisfaction; 5 point Likert scale.	Observational, analytical – cross sectional	Scales suitable for measuring interpersonal processes during CQI decision making and satisfaction with decisions. Process scales similar in content to scales measuring constructive controversy (e.g. [104]).
Janssen 1999 [105]	Other examples: none identified in health care.	Based on theories of interdependence and conflict in groups (e.g. [86, 106-107]).			[PROCESS, OUTCOME]
Extended Intragroup	Predictive/outcome: to examine the relationship	Intra-group conflict types: relationship conflict (disagreements and incompatibilities	14 items	Observational,	A widely used measure of intra-group

Instrument (name, index, references for main reports)	Main purpose (measurement aim and context of use; examples of use in health care)	Description of content (dimensions, sub-dimensions; conceptual/theoretical basis)	Items & response scale	Study design(s)	Comments [main domain(s) measured]
Conflict Scale	between intra-group conflict, group outcomes (performance, viability) and factors that moderate and mediate the effect of conflict in teams. (USA)	regarding personal issues), task conflicts (disagreements about the task being performed), process conflicts (disagreements about how tasks should be accomplished). Attitudes and beliefs about each conflict type: emotions (arising from conflict), conflict norms (open discussion of differing opinions), resolution efficacy (ability to resolve conflict), importance of the conflict episode (to those involved).	measuring type, 3 scales; 31 items measuring attitudes,3 scales; 7 point Likert scale.	analytical – cross sectional	conflict with use in a range of teams and settings. The extension adds measures of attitudes and beliefs about conflict [108].
Jehn 2008a [86, 108]	Other examples: Selected items and scales from an earlier version used to examine the relationship between (i) interpersonal processes and hospital CQI team performance [54]; (ii) conflict, team learning and performance in clinical care teams [109], (iii) group identification, conflict and productivity in primary care teams [110]. (USA, Netherlands, UK). No examples identified for this new scale.	Based on an extensive body of theoretical and empirical work on intragroup conflict (e.g. [111-112]), extended by the authors using findings from qualitative research [108, 113].			[PROCESS]
Team empowerment	Discriminative/predictive: to enable testing of the relationships between empowerment, its antecedents and performance in work teams. (USA)	Team empowerment, collective beliefs comprised of: group potency (belief the team can be effective), team meaningfulness (experience of the team task as significant, valuable and worthwhile), team autonomy (experience of freedom, independence, discretion in undertaking the task), team impact (experience that the output of the team's work is of importance to the organisation).	26 items, 4 subscales; 7 point Likert scale.<br><br>12 item short version [114]	Observational, analytical – cross sectional	Construct is important for investigating CQI theory, the measure has a strong theoretical basis, and the subscales can be used individually.
Kirkman 1999a [114-115]	Other examples: None identified. Model informed a measure of empowerment in hospital teams [116].	Based on a theoretical model of team empowerment developed by the authors [117], which builds on theories of individual empowerment (e.g. [118])			[EMERGENT]
Group reaction questionnaire	Predictive/outcome: to examine the relationship between conflict management style, task complexity and outcomes of decision making in QI teams in a government agency and a large corporation. (USA)	Group decision making processes: extent to which decision making processes (i.e. those used to analyse problems, establish goals and objectives, and evaluate positive and negative qualitative of solutions and options) are characterised by "inclusive, consensual goal-setting and the ability to analyse problems and propose solutions through a critical discussion that incorporates several perspectives" (p563)	29 items; 7 point Likert scale.	Observational, analytical – longitudinal	Content suitable for evaluating the quality of team decision making processes, focusing on team meetings. Items form a single scale; which may make it unfeasible when used with other measures.
Kuhn 2000 [119]	Other examples: none identified in health care.	Based on functional theory of group decision making (e.g. [120-121])			[PROCESS]
Team learning behaviours instrument	Predictive: to measure team learning behaviours as predictors of team performance. (Netherlands)	Team learning behaviours: co-construction of meaning, exploring different perspectives (explore, share knowledge, opinions and different perspectives), error analysis, error communication, reflection on processes, reflection on outcomes, feedback seeking behaviour, experimenting.	28 items, 8 subscales; 5 point Likert scale.	Instrument development	An important construct for understanding CQI team process. Adapts and extends existing measures of team learning through a systematic development process.
Savelsbergh 2009 [122]	Other examples: none identified	Based on theoretical and empirical research on team learning (e.g. [47, 49, 123])			[PROCESS]
Team reflexivity scales	Predictive/outcome: to measure team reflexivity enabling exploration of *reflection* as a predictor of a team's ability to *adapt* to changing circumstances and team effectiveness. (Netherlands)	Team reflection and adaptation (reflexivity): evaluation and learning (evaluation of completed task-work, reflecting on actions, adaptation), discussing processes (reflecting on team communication, norms and values), adaptation (carrying out planned actions, making agreed upon adaptations). Depth of reflection measured as: shallow ("thinking about and discussing issues related closely to the task at hand"), moderate (taking "a more critical approach toward work processes"), deep ("questioning the prevalent norms and values"). (Schippers 2007 , p195)	19 items, 3 scales; 5 point Likert scale.	Instrument development	Suitable for measuring reflexivity and adaption, an important element of team learning behaviours and relevant to CQI process.

Instrument<br>(name, index, references for main reports)	Main purpose<br>(measurement aim and context of use; examples of use in health care)	Description of content<br>(dimensions, sub-dimensions; conceptual/theoretical basis)	Items & response scale	Study design(s)	Comments<br><br>[main domain(s) measured]
Schippers 2007 [124]	Other examples: selected items used to examine differences in physician and nurse perceptions of team coordination in multi-professional patient care teams in hospital ICUs [125].	Based on theoretical and empirical research on team reflexivity, most notably [126].			[PROCESS]
Constructive controversy scale	Predictive: to measure interactions during problem solving (constructive controversy) as a predictor of decision quality and satisfaction with the decision making process. (Canada)	Constructive controversy: cooperation (collective attitude, not dominating), controversy (considering minority and opposing views), confirmation (disagreement not seen as criticism, non-blaming), collaboration (mutual influence, not controlling), differentiation (understanding the problem and possible solutions before decisions are made).	15 items; 5 point Likert scale.	Observational, analytical – cross sectional	Single scale, that measures an element of collaborative group process that is of relevance to decision making in CQI teams.
Tjosvold 1986 [104]	Other examples: none identified in health care.	Based on theories of the role of constructive controversy in group decision making (e.g. [127])			[PROCESS]
Beliefs about interpersonal context for team learning	Predictive: to examine the relationship between team interpersonal context, team learning behaviour, and team effectiveness. (Netherlands)	Beliefs about the interpersonal context for team learning: psychological safety, task and outcome interdependence, social and task cohesion, group potency.	25 items, 5 scales; response scale not reported	Observational, analytical – cross sectional	This measure combines some commonly used scales (or item thereof) to measure dimensions of context predicted to facilitate team learning. The scales can be used independently.
Van den Bossche 2006a [128]	Other examples: none identified, although the component scales have been used in other studies.	Based on a theoretical framework developed by the authors that integrates cognitive and social theories of group learning (e.g. [129-130]).			[EMERGENT]
Team learning behaviours questionnaire	Outcome/predictor: to examine the relationship between team interpersonal context, team learning behaviour, and team effectiveness. (Netherlands)	Team learning behaviours: construction of meaning (individual explanations of the problem and how to deal with it), co-construction of meaning (behaviours used to develop a shared understanding), constructive conflict (negotiation of the differences in interpretation among team members)	9 items; response scale not reported	Observational, analytical – cross sectional	A very relevant measure for understanding an aspect of CQI team process that may influence team effectiveness.
Van den Bossche 2006b [128, 131]	Other examples: No examples identified in health care. Used to examine the relationship between learning behaviour and quality of medical curricula developed by multi-disciplinary teams [132]. (Netherlands, Germany)	Based on a theoretical framework developed by the authors that integrates cognitive and social theories of group learning (e.g. [129-130]).			[PROCESS]
Team Diagnostic Survey	Predictive/diagnostic/discriminative: to provide a measure of teamwork in organisational settings that is suitable for research and the practical diagnosis of teams' strengths and weaknesses.	Enabling conditions: real team (clear boundaries, interdependence, stability), compelling direction (clear, challenging, consequential, end versus means), enabling structure (team composition, task design, norms of conduct), supportive organisational context, expert coaching (availability, helpfulness, extent and focus of team leader/peer behaviours). Team effectiveness: process criteria (effort, strategy, knowledge and skill), interpersonal processes (interaction quality, satisfaction with team relationships), individual learning and well-being.	103 items, 10 scales (30 subscales); 5 point Likert scale.	Instrument development	Instrument is comprised of multiple scales that can be used independently. Includes a comprehensive measure of Hackman's conceptualisation of perceived team effectiveness. Although one subscale was used in health care,
Wageman 2005 [133]	Other examples: one subscale (real team) was used to examine differences in physician and nurse perceptions of team function in multi-professional patient care teams in hospital ICUs [125].	Based on the model of team effectiveness proposed by Hackman and colleagues (e.g. [39, 55]).			[ENABLING CONDITIONS, EMERGENT, PROCESS, OUTCOME]

References

1. Kivimaki M, Kivimaki M, Elovainio M: **A short version of the Team Climate Inventory: Development and psychometric properties.** *J Occup Organ Psych* 1999, **72:**241-246.
2. Anderson N, West M: **Measuring climate for work group innovation: Development and validation of the Team Climate Inventory.** *J Organ Behav* 1998, **19:**235-258.
3. Anderson N, West M: *The team climate inventory: manual and users guide.* Windsor: Nelson Press; 1994.
4. Borril C, West MA, Shapiro D, Rees A: **Team working and effectiveness in health care** *Br J Healthc Manag* 2000, **6:**34-37.
5. Poulton BC, West MA: **The determinants of effectivenss in primary health care teams.** *J Interprof Care* 1999, **13:**7-18.
6. Bosch M, Dijkstra R, Wensing M, van der Weijden T, Grol R: **Organizational culture, team climate and diabetes care in small office-based practices.** *BMC Health Serv Res* 2008, **8:**180.
7. Hann M, Bower P, Campbell S, Marshall M, Reeves D: **The association between culture, climate and quality of care in primary health care teams.** *Fam Pract* 2007, **24:**323-329.
8. Proudfoot J, Jayasinghe UW, Holton C, Grimm J, Bubner T, Amoroso C, Beilby J, Harris MF: **Team climate for innovation: what difference does it make in general practice?** *Int J Qual Health Care* 2007, **19:**164-169.
9. Howard M, Brazil K, Akhtar-Danesh N, Agarwal G: **Self-reported teamwork in family health team practices in Ontario: organizational and cultural predictors of team climate.** *Can Fam Physician* 2011, **57:**e185-191.
10. Eccles MP, Hrisos S, Francis JJ, Stamp E, Johnston M, Hawthorne G, Steen N, Grimshaw JM, Elovainio M, Presseau J, Hunter M: **Instrument development, data collection, and characteristics of practices, staff, and measures in the Improving Quality of Care in Diabetes (iQuaD) Study.** *Implement Sci* 2011, **6**.
11. Fay D, Borrill C, Amir Z, Haward R, West MA: **Getting the most out of multidisciplinary teams: A multi-sample study of team innovation in health care.** *J Occup Organ Psychol* 2006, **79:**553-567.
12. West M: **The social psychology of innovation in groups.** In *Innovation and creativity at work: Psychological and Organizational Strategies.* Edited by West M, Farr J. Chichester: John Wiley and Sons; 1990: 309-330
13. West M, Anderson N: **Innovation in top management teams.** *J Appl Psychol* 1996, **81:**680-693.
14. Hearnshaw H, Reddish S, Carlyle D, Baker R, Robertson N: **Introducing a quality improvement programme to primary healthcare teams.** *Qual Health Care* 1998, **7:**200-208.

15. Baker R, Thompson J: **Innovation in general practice: is the gap between training and non-training practices getting wider?** *Br J Gen Pract* 1995, **45:**297-300.
16. Senior B: **Team roles and team performance: Is there "really" a link?** *J Occup Organ Psychol* 1997, **70:**241-258.
17. West MA, Wallace M: **Innovation in health care teams.** *Eur J Soc Psychol* 1991, **21:**303-315.
18. Lukas CV, Mohr DC, Meterko M: **Team effectiveness and organizational context in the implementation of a clinical innovation.** *Qual Manag Health Care* 2009, **18:**25-39.
19. VanDeusen Lukas C, Meterko MM, Mohr D, Seibert MN, Parlier R, Levesque O, Petzel RA: **Implementation of a clinical innovation: The case of advanced clinic access in the Department of Veterans Affairs.** *J Ambulatory Care Manage* 2008, **31:**94-108.
20. Mohr DC, VanDeusen Lukas C, Meterko M: **Predicting healthcare employees' participation in an office redesign program: attitudes, norms and behavioral control.** *Implement Sci* 2008, **3:**47.
21. Lemieux-Charles L, Murray M, Baker G, Barnsley J, Tasa K, Ibrahim S: **The effects of quality improvement practices on team effectiveness: a mediational model.** *J Organ Behav* 2002, **23:**533-553.
22. Shortell SM, Marsteller JA, Lin M, Pearson ML, Wu SY, Mendel P, Cretin S, Rosen M: **The role of perceived team effectiveness in improving chronic illness care.** *Med Care* 2004, **42:**1040-1048.
23. Shortell SM, Rousseau DM, Gillies RR, Devers KJ, Simons TL: **Organizational assessment in intensive care units (ICUs): construct development, reliability, and validity of the ICU nurse-physician questionnaire.** *Med Care* 1991, **29:**709-726.
24. Hall CB, Tennen H, Wakefield DB, Brazil K, Cloutier MM: **Organizational assessment in paediatric primary care: development and initial validation of the primary care organizational questionnaire.** *Health Serv Manage Res* 2006, **19:**207-214.
25. Brazil K, Wakefield DB, Cloutier MM, Tennen H, Hall CB: **Organizational culture predicts job satisfaction and perceived clinical effectiveness in pediatric primary care practices.** *Health Care Manage Rev* 2010, **35:**365-371.
26. Cloutier MM, Wakefield DB, Tsimikas J, Hall CB, Tennen H, Brazil K: **Organizational Attributes of Practices Successful at a Disease Management Program.** *J Pediatr* 2009, **154:**290-295.
27. Temkin-Greener H, Gross D, Kunitz SJ, Mukamel D: **Measuring interdisciplinary team performance in a long-term care setting.** *Med Care* 2004, **42:**472-481.
28. Cretin S, Shortell SM, Keeler EB: **An evaluation of collaborative interventions to improve chronic illness care. Framework and study design.** *Eval Rev* 2004, **28:**28-51.

29. Chan KS, Hsu YJ, Lubomski LH, Marsteller JA: **Validity and usefulness of members reports of implementation progress in a quality improvement initiative: Findings from the Team Check-up Tool (TCT).** *Implement Sci* 2011, **6**.
30. Cohen SG, Bailey DE: **What makes teams work: Group effectiveness research from the shop floor to the executive suite.** *J Manage* 1997, **23:**239-290.
31. Fried B, Topping S, Rundall T: **Groups and teams in health services organizations.** In *Health care management: Organization design and behavior.* 5th edition. Edited by Shortell SM, Kaluzny AD. Albany, NY: Delmar; 2006: 174-211
32. Caldwell DF, O'Reilly CA, III: **The determinants of team-based innovation in organizations. The role of social influence.** *Small Gr Res* 2003, **34:**497-517.
33. Strating MMH, Nieboer AP: **Norms for creativity and implementation in healthcare teams: testing the group innovation inventory.** *Int J Qual Health Care* 2010, **22:**275-282.
34. Campion MA, Medsker GJ, Higgs AC: **Relations between work group characteristics and effectiveness: Implications for designing effective work groups.** *Pers Psychol* 1993, **46:**823-850.
35. Campion MA, Papper EM, Medsker GJ: **Relations between work team characteristics and effectiveness: A replication and extension.** *Pers Psychol* 1996, **49:**429-452.
36. Brewer BB: **Relationships among teams, culture, safety, and cost outcomes.** *West J Nurs Res* 2006, **28:**641-653.
37. Gladstein DL: **Groups in context: A model of task group effectiveness.** *Adm Sci Q* 1984, **29:**499-517.
38. Guzzo RA, Shea GP: **Group performance and intergroup relations in organizations.** In *Handbook of industrial and organizational psychology, Vol 3 (2nd ed).* Edited by Dunnette MDEH, Leaetta M [Ed]. Palo Alto: Consulting Psychologists Press; 1992
39. Hackman JR: **The design of work teams.** In *Handbook of organizational behavior.* Edited by Lorsch JW. Englewood Cliffs, NJ: Prentice-Hall; 1987: 315-342
40. Tannenbaum SI, Beard RL, Salas E: **Team building and its influence on team effectiveness: An examination of conceptual and empirical developments.** In *Issues, theory, and research in industrial/organizational psychology.* Edited by Kelley KE. Amsterdam: Elsevier; 1992: 117-153
41. Costa AC, Anderson N: **Measuring trust in teams: Development and validation of a multifaceted measure of formative and reflective indicators of team trust.** *Eur J Work Org Pscychol* 2011, **20:**119-154.
42. Costa AC: **Work team trust and effectiveness.** *Pers Rev* 2003, **32:**605-622.
43. Costa AC, Roe RA, Taillieu T: **Trust within teams: The relation with performance effectiveness.** *Eur J Work Org Pscychol* 2001, **10:**225-244.

44. Dobson RT, Stevenson K, Busch A, Scott DJ, Henry C, Wall PA: **A quality improvement activity to promote interprofessional collaboration among health professions students.** *Am J Pharm Educ* 2009, **73:**64.

45. Clark PG: **Learning on interdisciplinary gerontological teams: intructional concepts and methods.** *Educ Gerontol* 1994, **20:**349-364.

46. Clark PG, Spence DL, Sheehan JL: **Challenges and barriers to interdisciplinary gerontological team training in the academic setting.** *Gerontol Geriatr Educ* 1987, **7:**93-110.

47. Edmondson A: **Psychological safety and learning behavior in work teams.** *Adm Sci Q* 1999, **44:**350-383.

48. Chan CA: **Examining the relationships between individual, team and organizational learning in an Australian hospital.** *Learning in Health and Social Care* 2003, **2:**223-235.

49. Argote L, Gruenfeld D, Naquin C: **Group learning in organizations.** In *Groups at work: theory and research.* Edited by Turner ME. Mahwah, N.J.: L. Erlbaum Associates, Publishers; 2001: xiv, 552 p.

50. Argyris C: *Reasoning, Learning and Action: Individual and Organizational.* San Francisco: Jossey-Bass; 1982.

51. Argyris C, Schön DA: *Organizational learning: a theory of action perspective.* Reading, MA: Addison-Wesley Publishing Company; 1978.

52. Nembhard IM, Edmondson AC: **Making it safe: the effects of leader inclusiveness and professional status on psychological safety and improvement efforts in health care teams.** *J Organ Behav* 2006, **27:**941-966.

53. Tucker AL: **An empirical study of system improvement by frontline employees in hospital units.** *Manufacturing and Service Operations Management* 2007, **9:**492-505.

54. Wilkens R, London M: **Relationships between climate, process, and performance in continuous quality improvement groups.** *J Vocat Behav* 2006, **69:**510-523.

55. Hackman J: *Groups that work (and those that don't): creating conditions for effective teamwork.* San Francisco: Jossey Bass; 1990.

56. Yeatts DE, Hyten C: *High performing self-managed teams: a comparison of theory to practice.* Thousand Oaks, CA: Sage; 1998.

57. Lewis K: **Measuring transactive memory systems in the field: Scale development and validation.** *J Appl Psychol* 2003, **88:**587-604.

58. Michinov E, Olivier-Chiron E, Rusch E, Chiron B: **Influence of transactive memory on perceived performance, job satisfaction and identification in anaesthesia teams.** *Br J Anaesth* 2008, **100:**327-332.

59. Wegner DM: **Transactive memory: A contemporary analysis of the group mind.** In *Theories of group behavior.* Edited by Mullen B, Goethals GR. New York: Springer-Verlag; 1987: 185-208
60. Moreland RL, Myaskovsky L: **Exploring the performance benefits of group training: Transactive memory or improved communication?** *Organ Behav Hum Decis Process* 2000, **82:**117-133.
61. Lichtenstein R, Alexander J, Jinnett A, Ullman E: **Embedded intergroup relations in interdisciplinary teams: Effects on perceptions of level of team integration.** *J Appl Behav Sci* 1997, **33:**413-434.
62. Alexander JA, Lichtenstein R, Jinnett K, Wells R, Zazzali J, Liu D: **Cross-functional team processes and patient functional improvement.** *Health Serv Res* 2005, **40:**1335-1355.
63. Lichtenstein R, Alexander JA, McCarthy JF, Wells R: **Status differences in cross-functional teams: effects on individual member participation, job satisfaction, and intent to quit.** *J Health Soc Behav* 2004, **45:**322-335.
64. Chin MH, Cook S, Drum ML, Jin L, Guillen M, Humikoski CA, Koppert J: **Improving diabetes care in Midwest community health centres with the health disparities collaborative.** *Diabetes Care* 2004, **27:**2-8.
65. Tajfel H: **An integrative theory of intergroup conflict.** In *The social psychology of intergroup relations.* Edited by Worchel S, Austin WG. Monterey, Calif.: Brooks/Cole Pub. Co.; 1979: xii, 369 p.
66. Tajfel H: *Social identity and intergroup relations.* Cambridge: Cambridge University Press; 1982.
67. Alderfer CP: **An intergroup perspective of group dynamics.** In *Handbook of organizational behavior.* Edited by Lorsch JW. Englewood Cliffs, NJ: Prentice-Hall; 1987: ix, 430 p.
68. Lubomski LH, Marsteller JA, Hsu YJ, Goeschel CA, Holzmueller CG, Pronovost PJ: **The team checkup tool: evaluating QI team activities and giving feedback to senior leaders.** *Jt Comm J Qual Patient Saf* 2008, **34:**619-623, 561.
69. Mills PD, Weeks WB: **Characteristics of successful quality improvement teams: lessons from five collaborative projects in the VHA.** *Jt Comm J Qual Saf* 2004, **30:**152-162.
70. Weeks WB, Mills PD, Dittus RS, Aron DC, Batalden PB: **Using an improvement model to reduce adverse drug events in VA facilities.** *Jt Comm J Qual Improv* 2001, **27:**243-254.
71. Weeks WB, Mills PD, Waldron J, Brown SH, Speroff T, Coulson LR: **A model for improving the quality and timeliness of compensation and pension examinations in VA facilities.** *J Healthc Manag* 2003, **48:**252-261.
72. Mills PD, Weeks WB, Surott-Kimberly BC: **A multihospital safety improvement effort and the dissemination of new knowledge.** *Jt Comm J Qual Saf* 2003, **29:**124-133.
73. Neily J, Howard K, Quigley P, Mills PD: **One-year follow-up after a collaborative breakthrough series on reducing falls and fall-related injuries.** *Jt Comm J Qual Patient Saf* 2005, **31:**275-285.

74. Nelson EC, Batalden PB, Huber TP, Mohr JJ, Godfrey MM, Headrick LA, Wasson JH: **Microsystems in health care: Part 1. Learning from high-performing front-line clinical units.** *Jt Comm J Qual Improv* 2002, **28:**472-493.

75. Parker VA, Wubbenhorst WH, Young GJ, Desai KR, Charns MP: **Implementing quality improvement in hospitals: the role of leadership and culture.** *Am J Med Qual* 1999, **14:**64-69.

76. Brannick MT, Salas E, Prince C (Eds.): **Team performance and assessment and measurement: theory, methods and applications.** Mahwah: Lawrence Erlbaum Associates; 1997.

77. DiBella AJ, Nevis EC: *How organizations learn : an integrated strategy for building learning capability.* 1st edn. San Francisco, Calif.: Jossey-Bass; 1998.

78. Millward LJ, Jeffries N: **The team survey: a tool for health care team development.** *J Adv Nurs* 2001, **35:**276-287.

79. Schouten L, Grol R, Hulscher M: **Factors influencing success in quality improvement collaboratives: development and psychometric testing of an instrument.** *Implement Sci* 2010, **5:**84.

80. Kilo CM: **Improving care through collaboration.** *Pediatrics* 1999, **103:**384-393.

81. Ovretveit J, Bate P, Cleary P, Cretin S, Gustafson D, McInnes K, McLeod H, Molfenter T, Plsek P, Robert G, et al: **Quality collaboratives: lessons from research.** *Qual Saf Health Care* 2002, **11:**345-351.

82. Wilson T, Berwick DM, Cleary PD: **What do collaborative improvement projects do? Experience from seven countries.** *Joint Commission Journal of Quality and Safety* 2003, **29:**85-93.

83. Thompson BM, Levine RE, Kennedy F, Naik AD, Foldes CA, Coverdale JH, Kelly PA, Parmelee D, Richards BF, Haidet P: **Evaluating the quality of learning-team processes in medical education: development and validation of a new measure.** *Acad Med* 2009, **84:**S124-127.

84. Hackman J: **Total quality mangement: empirical, conceptual and practical issues.** *Adm Sci Q* 1995, **40:**309-329.

85. Hackman JR, Wageman R: **A Theory of Team Coaching.** *Acad Manage Rev* 2005, **30:**269-287.

86. Jehn KA: **A multimethod examination of the benefits and detriments of intragroup conflict.** *Adm Sci Q* 1995, **40:**256-282.

87. Arnold JA, Arad S, Rhoades JA, Drasgow F: **The Empowering Leadership Questionnaire: the construction and validation of a new scale for measuring leader behaviors.** *J Organ Behav* 2000, **21:**249-269.

88. Manz CC, Sims HP: **Leading workers to lead themselves: The external leadership of self-managing work teams.** *Adm Sci Q* 1987, **32:**106-129.

89. Liden RC, Tewksbury TW: **Empowerment and work teams.** In *Handbook of Human Resource Management.* Edited by Reffis GR, Rosen SD, Barnum DT. Cambridge, Mass: Blackwell Publishers; 1995: 386-403

90. Schein EH: *Organizational culture and leadership.* 2nd edn. San Francisco: Jossey-Bass; 1992.

91. Bunderson JS, Sutcliffe KM: **Management team learning orientation and business unit performance.** *J Appl Psychol* 2003, **88:**552-560.

92. Bunderson J, Boumgarden P: **Structure and learning in self-managed teams: Why "bureaucratic" teams can be better learners.** *Organ Sci* 2010, **21:**609-624.

93. Grinyer PH, Yasai-Ardekani M: **Dimensions of organizational structure: A critical replication.** *Acad Manage J* 1980, **23:**405-421.

94. Coyle-Shapiro JAM, Morrow PC: **The role of individual differences in employee adoption of TQM orientation.** *J Vocat Behav* 2003, **62:**320-340.

95. Driskell JE, Salas E, Hughes S: **Collective orientation and team performance: development of an individual differences measure.** *Hum Factors* 2010, **52:**316-328.

96. Patterson PD, Weaver MD, Weaver SJ, Rosen MA, Todorova G, Weingart LR, Krackhardt D, Lave JR, Arnold RM, Yealy DM, Salas E: **Measuring teamwork and conflict among emergency medical technician personnel.** *Prehosp Emerg Care* 2011, **16:**98-108.

97. Oyserman D, Coon HM, Kemmelmeier M: **Rethinking individualism and collectivism: Evaluation of theoretical assumptions and meta-analyses.** *Psychol Bull* 2002, **128:**3-72.

98. Hiller NJ, Day DV, Vance RJ: **Collective enactment of leadership roles and team effectiveness: A field study.** *Leadership Quart* 2006, **17:**387-397.

99. Yukl G, Lepsinger R: **Preliminary report on validation of the Managerial Practices Survey.** In *Measures of leadership.* Edited by Clark KE, Clark MB. West Orange, NJ: Leadership Library of America; 1990: 223−237

100. Hoegl M, Gemuenden HG: **Teamwork quality and the success of innovative projects: A theoretical concept and empirical evidence.** *Organ Sci* 2001, **12:**435-449.

101. Pinto MB, Pinto JK: **Project team communication and crossfunctional cooperation in new program development.** *J Prod Innovat Manag* 1990, **7:**200-212.

102. Seers A, Petty MM, Cahsman JF: **Team-member exchange under team and traditional management: A naturally occuring quasi-experiment.** *Group Organ Manage* 1995, **20:**18-38.

103. Mullen B, Copper C: **The relation between group cohesiveness and performance: An integration.** *Psychol Bull* 1994, **Vol.115:**pp.

104. Tjosvold D, Wedley WC, Field RH: **Constructive controversy, the Vroom-Yetton model, and managerial decision-making.** *J Occup Behav* 1986, **7:**125-138.

105. Janssen O, Van de Vliert E, Veenstra C: **How task and person conflict shape the role of positive interdependence in management teams.** *J Manage* 1999, **25:**117-142.

106. Deutsch M: *The resolution of conflict: Constructive and destructive processes. .* New Haven: Yale University Press; 1973.

107. Tjosvold D: *The conflict-positive organization.* Boston: Addison-Wesley; 1991.

108. Jehn KA, Greer L, Levine S, Szulanski G: **The effects of conflict types, dimensions, and emergent states on group outcomes.** *Group Decis Negot* 2008, **17:**465–495.

109. van Woerkom M, van Engen ML: **Learning from conflicts? The relations between task and relationship conflicts, team learning and team performance.** *Eur J Work Org Pscychol* 2009, **18:**381-404.

110. Richter AW, West MA, Van Dick R, Dawson JF: **Boundary spanners' identification, intergroup contact, and effective intergroup relations.** *Acad Manage J* 2006, **49:**1252-1269.

111. De Dreu CKW, Weingart LR: **Task versus relationship conflict, team performance, and team member satisfaction: A meta-analysis.** *J Appl Psychol* 2003, **88:**741-749.

112. Barki H, Hartwick J: **Conceptualizing The Construct of Interpersonal Conflict.** *Int J Confl Manage* 2004, **15:**216–244.

113. Jehn KA: **A Qualitative Analysis of Conflict Types and Dimensions in Organizational Groups.** *Adm Sci Q* 1997, **42:**530-557.

114. Kirkman BL, Rosen B, Tesluk PE, Gibson CB: **The impact of team empowerment on virtual team performance: the moderating role of face-to-face interaction.** *Acad Manage J* 2004, **47:**175-192.

115. Kirkman BL, Rosen B: **Beyond self-management: antecedents and consequences of team empowerment.** *Acad Manage J* 1999, **42:**58-74.

116. Proenca EJ: **Team dynamics and team empowerment in health care organizations.** *Health Care Manage Rev* 2007, **32:**370-378.

117. Kirkman BL, Rosen B: **A model of work team empowerment** In *Research in organizational change and development: An annual series featuring advances in theory, methodology, and research. Volume* 10. Edited by Pasmore WA, Woodman RW: Elsevier Science/JAI Press; 1997: xii, 322

118. Spreitzer GM: **Psychological empowerment in the workplace: dimensions, mesurement and validation.** *Acad Manage J* 1995, **38:**1442-1465.

119. Kuhn T, Poole MS: **Do conflict management styles affect group decision making? Evidence from a longitudinal field study.** *Hum Commun Res* 2000, **26:**558-590.

120. Gouran DS, Hirokawa RY: **Functional theory and communication in decision making and problem-solving groups: An expanded view** In *Communication and group decision making.* 2nd edition. Edited by Hirokawa RY, Poole MS. Thousand Oaks, CA Sage; 1996: 55-80

121. Mayer ME: **Behaviors leading to more effective decisions in small groups embedded in organizations.** *Communication Reports* 1998, **11:**123-132.

122. Savelsbergh CM, van der Heijden BI, Poell RF: **The development and empirical validation of a multidimensional measurement instrument for team learning behaviors.** *Small Gr Res* 2009, **40:**578-607.

123. Kasl E, Marsick V, Dechant K: **Teams as learners: A research-based model on team learning.** *J Appl Behav Sci* 1987, **33:**227-246.

124. Schippers MC, Den Hartog DN, Koopman PL: **Reflexivity in teams: A measure and correlates.** *Appl Psychol Int Rev* 2007, **56:**189-211.

125. Adler-Milstein J, Neal K, Howell MD: **Residents' and nurses' perceptions of team function in the medical intensive care unit.** *J Crit Care* 2011, **26:**104.e107-115.

126. West MA: **Reflexivity and work group effectiveness: A conceptual integration.** In *Handbook of work group psychology.* Edited by West MA. Chichester, UK: John Wiley; 1996: 159-187

127. Mann L, Janis I: **Decisional conflict.** In *Productive Conflict Management. Perspectives For Organizations.* Edited by Tjosvold D, Jonson DW. Irvington, New York; 1983: 14-40

128. van den Bossche P, Gijselaers WH, Segers M, Kirschner PA: **Social and Cognitive Factors Driving Teamwork in Collaborative Learning Environments: Team Learning Beliefs and Behaviors.** *Small Gr Res* 2006, **37:**490-521.

129. Roschelle J: **Learning by collaborating: Convergent conceptual change.** *J Learn Sci* 1992, **2:**235-276.

130. Dillenbourg P, Baker M, Blaye A, O'Malley C: **The evolution of research on collaborative learning.** In *Learning in humans and machines : towards an interdisciplinary learning science.* Edited by Reimann P, Spada H. New York: Pergamon; 1995

131. van den Bossche P, Gijselaers W, Segers M, Woltjer G, Kirschner P: **Team learning: Building shared mental models.** *Instr Sci* 2011, **39:**283-301.

132. Stalmeijer RE, Gijselaers WH, Wolfhagen IHAP, Harendza S, Scherpbier AJJA: **How interdisciplinary teams can create multi-disciplinary education: The interplay between team processes and educational quality.** *Med Educ* 2007, **41:**1059-1066.

133. Wageman R, Hackman J, Lehman E: **Team Diagnostic Survey: Development of an Instrument.** *J Appl Behav Sci* 2005, **41:**373-398.
