# Supplementary material for: Measuring team factors thought to influence the success of quality improvement in primary care: a systematic review of instruments
Source: Implement Sci. 2013 Feb 14;8:20. doi: 10.1186/1748-5908-8-20 (PMC3602018; doi:10.1186/1748-5908-8-20)
Supplement: Additional file 6 — Table summarizing the characteristics of instruments included for categorization of content, but excluded from review of measurement properties (Stage four) (Table S8). [file 1748-5908-8-20-S6.pdf]

Additional file 6

Table S8: Characteristics of instruments included for categorisation of content but excluded from review of measurement properties (Stage four)

**Instrument** (name, index, references for main reports)	**Main purpose** (measurement aim and context of use)	**Description of content** (dimensions, sub-dimensions; conceptual/theoretical basis)	**Items & response scale**	**Study design(s)**	**Reason for exclusion and comments** [main domain measured]
Primary care settings					
Survey of improvement practices	Descriptive: to characterise the practices used by QI teams in hospitals or hospital affiliated clinics. (USA)	Items related to team process: time spent on improvement, patterns of problem solving, methods used for problem analysis and planning, methods used to make team meetings effective.	37 items; scales varied.	Observational, descriptive	Content is relevant, but would require modification to address varied format of items and response scales that makes the instrument unsuited to psychometric analysis.
Alemi 2011 [1]		Conceptual basis of measure not explicitly reported.			[PROCESS]
Team leadership clarity	Predictive: to examine the relationship between leadership clarity, team process and innovation in health care teams, including primary care teams. (UK)	Leadership clarity: extent to which there is an overall coordinator or leader in the team. Option include clear leader, multiple leaders, no leader, conflict of leadership, shared leadership among all team members.	1 item; 5 response options each describing a different leader approach.	Observational, analytical – cross sectional	Single item measure that could be used in primary care QI teams. Narrow construct, covered in other measures.
West 2003 [2]		Based on team performance literature (selected references cited)			[LEADER]
Other health care settings					
Team function questionnaire	Discriminative: to measure differences in physician and nurse perceptions of team function in multi-professional patient care teams in hospital ICUs. (USA)	Team function: presence of a real team (boundedness, interdependence, stability), communication (openness, accuracy, timeliness, satisfaction), collaborative decision-making, coordination (team planning, action after team planning)	33 items; 7 point Likert scale.	Observational, analytical - cross sectional	Three of the four scales are from instruments included in the review [3-5]. The fourth requires rewording for QI as it focuses on clinical care [6].
Adler-Milstein 2011 [7]		Authors reference related empirical and theoretical work, but conceptual framework for the overall study was not explicitly reported. The scales were intended to capture “multiple perspectives on team function” (p11).			[PROCESS, TEAM]
TeamSTEPPS teamwork attitudes questionnaire	Discriminative/outcome: to measure teamwork attitudes to assess training needs or evaluate attitudinal outcomes of the TeamSTEPPS training program. (USA)	Individual attitudes toward teamwork: team structure, leadership, mutual support, situation monitoring, communication. Scales focus on attitudes toward behaviours relevant to delivering team-based clinical care. Example items: “Patients are a critical component of the care team”, “It is appropriate to assert a patient safety concern until you are certain that it has been heard”, “Poor communication is the most common cause of reported errors”. (appendix, p1-3)	30 items, 5 scales; 5 point Likert scale.	Instrument development	Measures attitudes toward teamwork outcomes and behaviours in care teams. Some scales are not relevant to QI teams (situation monitoring), others require rewording of items and check for relevance.
Baker 2010 [8-9]		Based on the TeamSTEPPS training model from the Agency for Healthcare Research and Quality (AHRQ), which aims to develop core teamwork skills required of patient care teams.			[ANTECEDENT]
Team information processing scales	Predictive: to measure information exchange and utilisation (information processing) in clinical care teams as a predictor of patient satisfaction. (USA)	Team information processing behaviours: information exchange (sharing, discussing, and evaluating of information), information utilization (use of information transformed by the team).	9 items, 2 scales; 7 point Likert scale.	Observational – analytical, cross-sectional	Constructs are relevant to CQI teams, but scales refer to patient care. Would need rewording for QI team, and check to ensure content is relevant.

Instrument<br>(name, index, references for main reports)	Main purpose<br>(measurement aim and context of use)	Description of content<br>(dimensions, sub-dimensions; conceptual/theoretical basis)	Items & response scale	Study design(s)	Reason for exclusion and comments<br>[main domain measured]
Deeter-Schmelz 2003a [10]		Based on organisational learning and knowledge management theory (selected references) and model developed for the study by the authors.			[PROCESS]
Team norms scales	Predictive: to measure team behavioural norms as predictors of information processing in clinical care teams and patient satisfaction. (USA)	Team behavioural norms: norms of flexibility (willingness to adapt to change), norms of information exchange (willingness to exchange information), norms of solidarity (commitment to team relationships and goals).	9 items, 3 scales; 7 point Likert scale.	Observational – analytical, cross-sectional	Constructs are relevant, however items wording refers to norms of day-to-day work rather than project work and would require some modification for QI project teams.
Deeter-Schmelz 2003b [10]		Based on organisational learning and knowledge management theory (selected references) and model developed for the study by the authors.			[EMERGENT]
Supportive conditions for QI collaborative (QIC) implementation	Discriminative/predictive: Measurement of QIC implementation as a predictor of process and outcome improvement in QI teams participating in a national, hospital based QIC. (Netherlands)	QIC team organisation: roles, task interdependence, shared goals, communication (one of 3 scales in the overall instrument).	5 items, 1 scale; 7 point Likert scale	Instrument development	A very general measure with limited utility as a stand alone measure of team factors (each construct covered by a single item). The full instrument is very relevant to measuring QI context (included in CQI context review).
Duckers, 2008 [11]		Based on literature pertaining to QIC success, team performance, and the role of leaders and change agents' in supporting adoption of innovations.		Pre- and post-intervention evaluation	[PROCESS, ANTECEDENTS, EMERGENT]
Team skills scale	Outcome: to measure self-assessed team skills following geriatric interdisciplinary team training (GITT). (USA)	Individual skills for performing behaviours required of an interdisciplinary team (ability to work effectively on a team; perform roles relevant to their professional; handle disagreements; strengthen cooperation and participation; contribute to meetings, team planning and strategies; monitor team performance and provide feedback)	17 items; 5 point Likert scale.	Pre- and post-intervention evaluation	Individual level measure of self-reported skill for teamwork behaviours required in interdisciplinary care teams. Would need some rewording for QI teams, and check to ensure relevance of behaviours.
Fulmer 2005 [12]		Based on the learning objectives of the geriatric interdisciplinary team training program.			[ANTECEDENT]
CQI knowledge, attitudes and beliefs (team approach items)	Outcome: to evaluate change in knowledge, attitudes and beliefs about CQI following an undergraduate course in CQI methods. (USA)	Attitudes toward the use of a team approach in CQI, focussing on perceived outcomes of teamwork (one of six scales in overall instrument).	7 items; 5 point Likert scale.	Pre- and post-intervention evaluation	Instrument is CQI specific, but no evidence that the scale can be used as a stand alone measure (construct not defined, no assessment of structure).
Gould 2002 [13]		Conceptual basis of measure not reported.			[ANTECEDENT]
Attitudes Toward Health Care Teams Scale	Outcome: to evaluate clinically based team training programs for medical and health profession students. (USA)	Attitudes toward team structure, process and functioning: Quality of care and care process; physician centrality.	19 items; 6 point Likert scale.	Instrument development	Individual level measure of attitudes toward teamwork outcomes and behaviours in interdisciplinary care teams. Would need some rewording for QI team, and check to ensure relevance of behaviours and outcomes.
Heinemann 1999 [14]		Based empirical research on attitudes of health professionals to teamwork (multiple sources cited).			[ANTECEDENT]

Instrument (name, index, references for main reports)	Main purpose (measurement aim and context of use)	Description of content (dimensions, sub-dimensions; conceptual/theoretical basis)	Items & response scale	Study design(s)	Reason for exclusion and comments [main domain measured]
Team identification scale	Predictive: to examine whether team identification and perceived team success are predictive of attitudinal changes and individual outcomes in staff participating in CQI teams in hospitals. (Canada)	Team identification: being accepted as a member of the team; personal contribution to the outcome of the team; and responsibility for the outcomes of the team	3 items; 5 point Likert scale.	Observational, analytical – longitudinal	Short scale, used in study of CQI teams in hospital setting. Items not reported in full.
Irvine 2000a [15]		Based on social identification theory and empirical testing of this theory in QI circles [16].			[EMERGENT]
Perceived team effectiveness scale	Predictive: to examine whether team identification and perceived team success are predictive of attitudinal changes and individual outcomes in staff participating in CQI teams in hospitals. (Canada)	Perception of team success: degree of effectiveness of the CQI team in generating solutions to problems, implementation of suggested by organisation, overall success of CQI team.	7 items; 5 point Likert scale.	Observational, analytical – longitudinal	Used in study of CQI teams in hospital setting, but only 3 of 7 items reported. Similar to Lemieux-Charles 2002 measure of perceived team effectiveness [17].
Irvine 2000b [15]		Based on social identification theory as applied to QI circles [16].			[OUTCOME]
Group interaction scales	Outcome: to measure team member interactions as an outcome of an intervention to train hospital based health professionals in QI theory and methods. (Canada)	Group interaction: positive interaction (expectation and integration, organisation, communication and participation); negative interaction (power struggle, non-involvement).	26 items, 2 scales; 5 point Likert scale.	Experimental – randomised trial	Unclear which items were used from original scale [18]. Original source reports truncated items, providing sufficient data to illustrate content, but not to assess suitability of the measure for primary care.
Irvine 2002 [19]		Uses a subset of items developed to measure group interaction during collaborative tasks [18, 20].			[PROCESS]
Influence discrepancy scale	Predictive: to measure influence over team decisions as a predictor of work satisfaction in cross-functional teams in hospitals. (USA)	Influence discrepancy: discrepancy between 1) the degree of influence an individual thinks they should have over team decisions and 2) the degree of influence an individual perceives they actually have over team decision	2 items; 5 point Likert scale.	Observational, analytical – cross sectional	Scale focuses on influence over clinical decisions, some rewording required for QI teams
Lichtenstein 2004 [21]		Based on the theory of status characteristics and expectation states, and the value attainment theory of job satisfaction.			[EMERGENT]
Team type	Discriminative/predictive: to identify the dominant team type in human service organisations, and the relationship between team type and efficiency. (Sweden)	Categorisation of teams as multi-professional, inter-professional or trans-professional based on team functioning across six dimensions: role specialisation, task interdependence, co-ordination, task specialization, leadership and role interdependence.	6 items; 3 point response scales with descriptors for each rating.	Observational, analytical – cross sectional	Concept is relevant, but items are designed for clinical care teams, so would require rewording and check to ensure relevance in QI teams.
Thylefors 2005 [22]		Based on models of organising cross-functional teams.			[TEAM, PROCESS]
Perceived implementation success	Outcome: to examine the relationship between team learning behaviours and perceived implementation success in neonatal intensive care units (NICU) participating in a QIC. (USA, Canada)	Perceived success of implementing new practices (agreement that the QI projects are making a difference to the process of care, staff behaviours have changed to match new practice, and the projects have improved the care)	3 items; 7 point Likert scale.	Observational, analytical – cross sectional	Measure of perceived team success, used in healthcare QI project in NICU. Could be reworded for other settings.
Tucker 2007 [23]		Measure based on previous studies of QI in NICUs [24].			[OUTCOME]
Enabling conditions for effective teamwork	Predictive: to test a model of the relationship between conditions for teamwork, enabling or mediating conditions, and team outcomes in hospital mental health care teams. (USA)	Team process (enabling conditions): effort (presence at meetings), knowledge and skills applied to taskwork (interdisciplinary collaboration).<br><br>The instrument includes scales measuring initial conditions (task clarity,	10 items; 5 or 7 point Likert scales.	Observational, analytical – cross sectional	Scales were written for interdisciplinary teams developing treatment plans. Would need rewording for QI

Instrument<br>(name, index, references for main reports)	Main purpose<br>(measurement aim and context of use)	Description of content<br>(dimensions, sub-dimensions; conceptual/theoretical basis)	Items & response scale	Study design(s)	Reason for exclusion and comments<br>[main domain measured]
		feelings of influence over teammates, availability of consultation, environmental support) and outcomes (treatment standards, cohesion, team effectiveness). Content requires substantive adaptation for CQI teams, so these scales were excluded from content analysis.			teams, and check to ensure relevance of behaviours.
Vinokur-Kaplan 1995 [25]		Instrument used in this study is designed to operationalise Hackman's model of team effectiveness [26-27].			[PROCESS]
Non health care					
Team performance questionnaire	Discriminative: to analyse work team performance for the purpose of remunerating manufacturing teams. (USA)	Team performance: open communication ; team comfort; decision making; team differences; ground rules; problem solving; satisfaction; resources; data-based decisions; quality; planning; cooperation.	70 items, 12 subscales; 5 point Likert scale.	Instrument development	Scales appear relevant, but content is covered by measures with a clear conceptual basis and provenance (e.g. [28-30]).
Allen 1998 [31]	Citation searches did not identify any studies referencing this paper.	No information reported about the conceptual basis of the instrument, or empirical or theoretical research to which this measure might relate. The source of instrument content is not reported, and the constructs are not described or defined.			[CONTEXT, PROCESS, OUTCOME]
Trust, creativity and collaboration scales	Predictive: to examine the relationship between intra-team trust, collaboration and of team creativity in a laboratory study (undergraduate student teams). (USA)	Group trust (affect and cognitive), team creativity, collaborative culture	22 items, 4 scales; 7 point Likert scale.	Observational, analytical – cross sectional	Constructs are relevant but well covered by instruments with use in healthcare (e.g. [28, 32-33])
Barczak 2010 [34]	Citation searches did not identify any studies referencing this paper.	Based on empirical research on collaboration and creativity in teams, the authors adapted pre-existing scales.			[EMERGENT]
Team commitment scale	Outcome/predictor: to examine the relationship between commitment and support at organisational, group and team level in a teams from a range of organisational settings. (USA)	Team commitment: identification with the team as characterised by a) commitment to the team goals, b) willingness to exert effort on behalf of the team, c) desire to remain as a member of the team (e.g. "I would accept almost any job in order to keep working on this team").	8 items; 7 point Likert scale.	Observational, analytical – cross sectional	Construct is relevant, but scale requires rewording for QI teams in primary care as items infer that the team is the respondent's primary work team (see example item).
Bishop 2005 [35-36]	Items from this scale have been used and adapted in other papers (e.g. [37-39])	Adapted from a measure of affective organisational commitment [40].			[EMERGENT]
Group process scales	Predictive/outcome: to examine the relationships between job design, group processes (reflexivity, cooperation, social support), group initiative, and self-organisational activities in industrial work groups. (Sweden)	Group process: reflexivity, cooperation, social support	15 items; 7 point Likert scale.	Observational, analytical – cross sectional	Items largely derived from other instruments included in the review [41-42]. These scales reflect an adaption of these measures rather than new scales.
Brav 2009 [43]		Based on theories relating job design and group process to innovation, primarily from a review on West [44].			[PROCESS]
Information sharing	Predictive: antecedent variable in a model testing the association between team structure, process (information sharing) and learning orientation in teams in the manufacturing sector [45] (USA)	Information sharing among team members (freedom with which information is shared; priority and effort made to share information and ensure all members are informed of issues affecting the team).	4 items; 7 point Likert scale.	Observational, analytical – cross sectional	One of multiple instruments measuring information sharing. Wording of this scale is less suited to project teams than other included measures (e.g. [30, 46-47]).
Bunderson 2010b [48]		Based on empirical and theoretical research on team learning (e.g. [41, 45])			[PROCESS]

**Instrument** (name, index, references for main reports)	**Main purpose** (measurement aim and context of use)	**Description of content** (dimensions, sub-dimensions; conceptual/theoretical basis)	**Items & response scale**	**Study design(s)**	**Reason for exclusion and comments** [main domain measured]
Team cohesion scales	Predictive: to examine the relationship between models of work group cohesion and team outcomes (perceived effectiveness, job satisfaction) in public service teams. (Australia)	Team cohesion: social cohesion, task cohesion, individual attraction to the group	10 items; 9 point Likert scale.	Instrument development	Item wording implies the team is the respondent's primary work team. The task cohesion scale is used in another included instrument [47].
Carless 2000a [49]	The task cohesion scale was used in another included instrument [47], with a social cohesion scale from another source suitable for QI teams.	Based on conceptual and theoretical work on cohesion in teams e.g. [50]			[EMERGENT]
Global transformational leadership scale	Predictive/discriminative: to develop a short measure of transformational leadership. Initial testing in the finance sector. (Australia)	Transformational leadership behaviours: communicates vision; supports staff development; gives encouragement and recognition; empowers team members fostering trust, cooperation and involvement; encourages innovative thinking; leads by example; exhibits charisma through competence and instilling pride and respect.	7 items; 5 point Likert scale.	Instrument development	Scale is shorter than widely used measures [51-52], and content is suitable for primary care however it measures leadership in general, not in relation to teams or QI.
Carless 2000b [53]	Used to measure leadership style as a predictor of team efficacy, job satisfaction, and well-being in health professionals [54].	Based on the theory of transformational leadership, in particular the conceptualisation from Podsakoff and colleagues [55].			[LEADER - EXTERNAL]
Team process scale	Outcome/predictor: to examine the effect of intra-team trust on team process and performance in tax consulting teams. (Netherlands)	Team process: reflexivity (reflection on goals, strategies, process), team monitoring (monitoring whether members meet obligations, perform as expected, progress satisfactorily), effort (made toward achieving team goals)	15 items, 3 scales; 5 point Likert scale.	Observational, analytical – cross sectional.	Short, generic scales but constructs well covered by other instruments (e.g. [4, 30]). Focus on monitoring individual (rather than team) is inconsistent with CQI principles.
de Jong 2010a [56]		Based on Mark's framework for understanding team process [57]. Reflexivity based on West's theoretical work on reflexivity in teams [58].			[PROCESS]
Team trust scale	Predictive: to examine the relationship between intra-team trust, team process and performance in tax consulting teams. (Netherlands)	Team trust (to help and keep members informed, take other members' interests into account, overall trust)	5 items; 5 point Likert scale.	Observational, analytical – cross sectional.	Single generic scale, but construct is well covered by instruments with use in healthcare (e.g. [33]) and in QI teams (e.g. [59]).
de Jong 2010b [56]		Based on existing measures of team trust in Dutch (e.g. [60-61]).			[EMERGENT]
Self-efficacy for teamwork	Predictive: to identify the relationship between individual difference variables (including self-efficacy for teamwork), individual preferences for teamwork (collectivistic orientation), cooperation and team performance among managerial students. (USA)	Self-efficacy for teamwork - perceived ability to: contribute, facilitate communication, delegate, coordinate tasks, resolve conflict, integrate multiple views in planning, and assume a leadership role.	8 items; 5 point Likert scale.	Observational, analytical – longitudinal.	Single generic scale, but construct is covered by healthcare specific measures, for example measures of efficacy for working on inter-disciplinary teams [62].
Eby 1997 [63]		Based on Bandura's theory of self-efficacy.			[ANTECEDENT]
Group potency scale	Predictive/outcome: to explore the determinants and outcomes of group potency in any context. (USA)	Group potency (shared belief among team members that the group "can be effective" (p87)): the team has confidence in itself, can produce quality work, solve problems and be productive, will be known as high performing, and will have influence.<br>Example items: "This team expects to be known as a high-performing team", "This team expects to have a lot of influence around here".	8 items; 5 point Likert scale.	Conceptual paper	Very widely used and adapted scale, but this construct is well covered by other measures and would need rewording for primary care (items suggest team is part of a larger organisational structure).

Instrument (name, index, references for main reports)	Main purpose (measurement aim and context of use)	Description of content (dimensions, sub-dimensions; conceptual/theoretical basis)	Items & response scale	Study design(s)	Reason for exclusion and comments [main domain measured]
Guzzo 1993 [64]	Adapted versions of the scale used in included measure [47].	Based on theoretical work that distinguishes potency from collective efficacy, and collective motivation. Note, Guzzo defines collective efficacy as an individual's perception of the group. Others conceive collective efficacy as a shared perception that differs from potency in that is task specific [65].			[EMERGENT]
Team identification scale	Predictive: to examine the relationship between team identification and individual team member behaviours (citizenship, creativity) in middle management teams. (Netherlands)	Team members' identification with the team: self-categorization (identifying with other members, sees team as reflection of self), team self-esteem (pride and respect for the team), team commitment (desire to continue with the team).<br>Example items: "My team is an important reflection of who I am", "I would rather not tell anyone I work with this team", "I would rather belong to another team".	10 items; 7 point Likert scale.	Observational, analytical – cross sectional.	Construct is relevant, but items infer that team is the respondent's primary work unit and one of multiple teams of which the respondent could be a member. Hence not suited to QI teams in primary care.
Janssen 2008 [66]		Based on social identity theory and related empirical research in teams (e.g. [67]). Items adapted from another source [68].			[EMERGENT]
Emergent states scale	Predictive: to examine the relationship between intra-group conflict, group outcomes (performance, viability) and factors that moderate and mediate the effect of conflict in teams in a laboratory study (undergraduate students). (USA)	Attitudes, values, motivations and cognitions arising from working as a team: trust, respect, cohesiveness.<br>Example items: "How well did members seem to know each other in this team", "How concerned were you about maintaining a friendship with the other team members".	9 items; 7 point Likert scale.	Observational, analytical – cross sectional.	A short, generic measure that may be useful for CQI evaluation if longer scales are not feasible. However, wording of some items may be incongruent with the relations in a small primary care setting and constructs are well covered by other measures.
Jehn 2008b [69]		Conceptual basis and source of items not reported.			[EMERGENT]
Team viability scale	Outcome: to examine the relationship between intra-group conflict, group outcomes (performance, viability) and factors that moderate and mediate the effect of conflict in teams in a laboratory study (undergraduate students). (USA)	Team viability: "member satisfaction and their behavioural intent to continue working together" (p467)	4 items; 7 point Likert scale.	Observational, analytical – cross sectional.	A short measure of viability, with items suitable for QI teams. However construct is covered by measures with use in QI teams in health care (e.g. [17])
Jehn 2008c [69]		Based on conceptualisations of team viability (primarily [70]).			[OUTCOME]
Leadership climate scale	Predictive: to measure leadership climate as a potential antecedent of work team empowerment in diverse organisations. (USA)	Leadership behaviours that support team empowerment, as exhibited by a leader external to team (giving responsibility for setting its own goals and conducting its work, asking for advice in decision making, articulating high performance expectations, exhibiting trust).	14 items; 7 point Likert scale.	Observational, analytical – cross sectional.	Construct is relevant, but only 7 of 14 items are reported (sufficient for assessing overall content, but not for inclusion for full review).
Kirkman 1999b [71]		Based on a theoretical model of team empowerment developed by the authors [72], with empowering leadership behaviours based on previous empirical research [73].			[LEADER]
Team autonomy and interdependence scales	Predictive: to examine interactions between individual and team level autonomy, interdependence and team performance in manufacturing teams. (USA)	Team level autonomy (discretion and freedom in decision making, planning and conduct of tasks) and interdependence (extent to which task requires coordination and contribution from all team members).	15 items, 2 scales; 9 point Likert scale.	Observational, analytical – cross sectional.	Constructs relevant to QI teams and items are short and generic. However, content is well covered by measures developed for QI teams in health care (e.g. [74-75]) and other measures [5, 71].
Langfred 2005 [76]		Based on theoretical and empirical research on interdependence (e.g. [77]) and autonomy (e.g. [78]).			[TASK]

**Instrument** (name, index, references for main reports)	**Main purpose** (measurement aim and context of use)	**Description of content** (dimensions, sub-dimensions; conceptual/theoretical basis)	**Items & response scale**	**Study design(s)**	**Reason for exclusion and comments** [main domain measured)
Self-Management Leadership Questionnaire (SMLQ)	Predictive: to identify leadership behaviours that support manufacturing teams to self-manage. (USA)	Self-management leader behaviours: encouraging rehearsal, self goal setting, self criticism, self reinforcement, self expectation and self-observation/evaluation.<br>Example items: In relation to external leader behaviour "He/she encourages us to practice a new task before we do it the first time", "He/she encourages us to praise each other if we have done a job well".	22 items, 6 scales; 7 point Likert scale.	Instrument development	Item wording reflects the manufacturing origins of the concept of self-managed teams, inferring a managerial role unlikely to resonate in health care.
Manz 1987 [79]	Used and adapted in multiple studies (e.g. [71, 80-81])	Based on self-management theory, socio-technical systems theory, and leadership, reinforcement, and goal-setting theories			[LEADER]
Team process	Predictive: to examine the relationship between team inputs (HR practices, leadership, support, work design), empowerment, team processes, and team performance in engineering teams. (Canada)	Team process: transition processes (planning, analysis, goal setting), action processes (tasks work, coordinating actions, monitoring progress), and interpersonal processes (managing conflicts, motivation, affect).<br>Example items: "members of my team discuss what we can do day to day to make our performance vision a reality", "members of my team effectively communicate with each other throughout the workday". (p102)	9 items, 3 scales; 5 point Likert scale.	Observational, analytical – cross sectional.	Short scales. Some items infer that the team is the respondent's primary work team, so require rewording. However, there are multiple included instruments that focus on behaviours required of CQI teams (e.g. [17, 75]).
Mathieu 2006 [82]		Based on three categories from Mark's framework and taxonomy of team processes [57].			[PROCESS]
Team member exchange (TMX) quality questionnaire	Predictive: to examine the relationship between the exchange relationships (individuals with other team members and with team leaders) and job attitudes in teams in the manufacturing sector. (USA)	Quality of an individual's exchange with other team members (willingness to help, share ideas and give feedback; how readily assistance, information and feedback are given).<br>Example items: "Other members recognize my potential", "Other members understand my problem", "I let others know when they affect my work".	14 items; 5 point Likert scale.	Observational, analytical - longitudinal	The concept of interaction quality is relevant, but items focus on individual perceptions of their relations with others. Measures of how the team interacts as a whole are more salient for CQI.
Seers 1989, Eby 1997 [63, 83]	Used and adapted in multiple other studies (e.g. [63, 84-85])	Based on an extension of theories about role-making and social exchange, especially those relating to leader-member exchange.			[PROCESS]
Preference for group work, collective and individual orientation scales	Predictive: to examine how collective versus individual orientations influence satisfaction with teamwork and perceived performance in a laboratory study (undergraduate student teams). (USA)	Collectivism: preference for working in groups, collective (beliefs about team process and outcomes) or individualistic orientation (beliefs about individual work).<br>Example items: "I get upset when people win", "only those who depend on themselves get ahead in life", "I tend to work harder when I am competing against other people".	22 items, 5 scales; 7 point Likert scale.	Observational, analytical - longitudinal	Measures attitudes toward teamwork, but construct is covered by instruments more suitably worded for primary care (e.g. [62, 86]).
Shaw 2000 [87]		Based on theoretical and empirical research on individual preferences for group work (individualism/collectivism), especially Wagner [88].			[ANTECEDENT]
Knowledge sharing scale	Outcome: to examine the effects of trust and task interdependence in technology teams with varying degrees of virtualness. (Canada)	Knowledge sharing: exchange of tacit and explicit knowledge, including ideas, expertise and perspectives.	5 items; 7 point Likert scale.	Observational, analytical – cross sectional	Short, generic scale but measures a construct extensively covered by other included measures that are more useful for CQI (e.g. [30, 46-47])
Staples 2008a [89]		Based on a three dimensional conceptualisation of knowledge (explicitness, reach, life-cycle) [90].			[PROCESS]
Trust within teams scale	Predictive: to examine the effects of trust and task interdependence in technology teams with varying degrees of virtualness. (Canada)	Trust in other team members (overall feelings of trust, comfort with others taking responsibility for important issues or critical tasks, trust that others will complete tasks and can be relied upon for help)	6 items; 7 point Likert scale.	Observational, analytical – cross sectional	Short generic scale, but construct is well covered by instruments with use in

**Instrument** (name, index, references for main reports)	**Main purpose** (measurement aim and context of use)	**Description of content** (dimensions, sub-dimensions; conceptual/theoretical basis)	**Items & response scale**	**Study design(s)**	**Reason for exclusion and comments** [main domain measured)
					healthcare (e.g. [33]) and in QI teams (e.g. [59]).
Staples 2008b [89]		Instrument from an unpublished source. Conceptual basis not reported.			[EMERGENT]
Interdependence scale	Predictive: to examine the effects of trust and task interdependence in technology teams with varying degrees of virtualness. (Canada)	Interdependence: extent to which tasks performed by different members must be coordinated and relate each other, individual goal attainment depends on others achieving goals and contributions from other members, success for one implies success for all in the team.	6 items; 7 point Likert scale.	Observational, analytical – cross sectional	Construct is relevant, but well covered by other included instruments (especially [5, 42, 47])
Staples 2008c [89]		Items were derived from other sources [29, 35].			[TASK]
Team interdependence	Predictive: to examine the relationship between interdependence, other job characteristics, and work outcomes in teams in the police force and schools. (Netherlands)	Team interdependence: task (extent to which task depends on sharing of information, expertise, completion of others' work), goal (extent to which there is collective responsibility for team goals), outcome (extent to which personal benefits accrue – including achievement of own goals - from team performance). Task interdependence comprise of: initiated (extent to which others depend on an individual), received (extent to which an individual depends on others).<br>Example items: "to what extent do your colleagues depend on you for doing their work well", "it is advantageous for me when my colleagues succeed in their jobs".	20 items, 4 scales; 5 point Likert scale.	Observational, analytical – cross sectional	Construct is relevant, but well covered by other included instruments (especially [5, 42, 47]). Some items would require rewording for QI teams.
van der Vegt 1998 [91-92]	Widely used and adapted (e.g. [39, 47, 93]), including development by the authors of an additional scale measuring goal interdependence [92].	Based on extensions of Hackman's job characteristics model to teams [77, 94].			[TASK]

References

1. Alemi F, Safaie FK, Neuhauser D: **A survey of 92 quality improvement projects.** *Jt Comm J Qual Improv* 2001, **27:**619-632.
2. West MA, Borrill CS, Dawson JF, Brodbeck F, Shapiro DA, Haward B: **Leadership clarity and team innovation in health care.** *Leadership Quart* 2003, **14:**393-410.
3. Shortell SM, Rousseau DM, Gillies RR, Devers KJ, Simons TL: **Organizational assessment in intensive care units (ICUs): construct development, reliability, and validity of the ICU nurse-physician questionnaire.** *Med Care* 1991, **29:**709-726.
4. Schippers MC, Den Hartog DN, Koopman PL: **Reflexivity in teams: A measure and correlates.** *Appl Psychol Int Rev* 2007, **56:**189-211.
5. Wageman R, Hackman J, Lehman E: **Team Diagnostic Survey: Development of an Instrument.** *J Appl Behav Sci* 2005, **41:**373-398.
6. Baggs JG: **Development of an instrument to measure collaboration and satisfaction about care decisions.** *J Adv Nurs* 1994, **20:**176-182.
7. Adler-Milstein J, Neal K, Howell MD: **Residents' and nurses' perceptions of team function in the medical intensive care unit.** *J Crit Care* 2011, **26:**104.e107-115.
8. Baker DP, Amodeo AM, Krokos KJ, Slonim A, Herrera H: **Assessing teamwork attitudes in healthcare: development of the TeamSTEPPS teamwork attitudes questionnaire.** *Qual Saf Health Care* 2010, **19:**e49.
9. Baker DP, Krokos KJ, Amodeo AM: **Instrument: TeamSTEPPS Teamwork Attitudes Questionnaire (T-TAQ)** [http://teamstepps.ahrq.gov/abouttoolsmaterials.htm]
10. Deeter-Schmelz DR, Ramsey RP: **An Investigation of Team Information Processing in Service Teams: Exploring the Link Between Teams and Customers.** *J Acad Market Sci* 2003, **31:**409-424.
11. Duckers MLA, Wagner C, Groenewegen PP: **Developing and testing an instrument to measure the presence of conditions for successful implementation of quality improvement collaboratives.** *BMC Health Serv Res* 2008, **8:**172.
12. Fulmer T, Hyer K, Flaherty E, Mezey M, Whitelaw N, Jacobs MO, Luchi R, Hansen JC, Evans DA, Cassel C, et al: **Geriatric interdisciplinary team training program: evaluation results.** *J Aging Health* 2005, **17:**443-470.
13. Gould BE, Grey MR, Huntington CG, Gruman C, Rosen JH, Storey E, Abrahamson L, Conaty AM, Curry L, Ferreira M, et al: **Improving patient care outcomes by teaching quality improvement to medical students in community-based practices.** *Acad Med* 2002, **77:**1011-1018.

14. Heinemann G, Schmitt MH, Farrell M, Brallier S: **Development of an attitudes toward health care teams scale.** *Eval Health Prof* 1999, **22:**123-142.

15. Irvine DM, Leatt P, Evans MG, Baker GR: **The behavioural outcomes of quality improvement teams: the role of team success and team identification.** *Health Serv Manage Res* 2000, **13:**78-89.

16. Steel RP, Mento AJ, Dilla BL, Ovalle Ii NK, Lloyd RF: **Factors Influencing the Success and Failure of Two Quality Circle Programs.** *J Manage* 1985, **11:**99.

17. Lemieux-Charles L, Murray M, Baker G, Barnsley J, Tasa K, Ibrahim S: **The effects of quality improvement practices on team effectiveness: a mediational model.** *J Organ Behav* 2002, **23:**533-553.

18. Watson W, Michaelsen LK, Sharp W: **Member Competence, Group Interaction, and Group Decision Making: A Longitudinal Study.** *J Appl Psychol* 1991, **76:**803-809.

19. Irvine Doran DM, Baker GR, Murray M, Bohnen J, Zahn C, Sidani S, Carryer J: **Achieving clinical improvement: an interdisciplinary intervention.** *Health Care Manage Rev* 2002, **27:**42-56.

20. Watson WE, Michaelsen LK: **Group Interaction Behaviors that Affect Group Performance on an Intellective Task.** *Group Organ Stud* 1988, **13:**495-516.

21. Lichtenstein R, Alexander JA, McCarthy JF, Wells R: **Status differences in cross-functional teams: effects on individual member participation, job satisfaction, and intent to quit.** *J Health Soc Behav* 2004, **45:**322-335.

22. Thylefors I, Persson O, Hellstrom D: **Team types, perceived efficiency and team climate in Swedish cross-professional teamwork.** *J Interprof Care* 2005, **19:**102-114.

23. Tucker AL, Nembhard IM, Edmondson AC: **Implementing New Practices: An Empirical Study of Organizational Learning in Hospital Intensive Care Units.** *Manage Sci* 2007, **53:**894-907.

24. Baker GR, King H, MacDonald JL, Horbar JD: **Using organizational assessment surveys for improvement in neonatal intensive care.** *Pediatrics* 2003, **111:**e419-425.

25. Vinokur-Kaplan D: **Treatment teams that work (and those that don't): An application of Hackman's group effectiveness model to interdisciplinary teams in psychiatric hospitals.** *J Appl Behav Sci* 1995, **31:**303-327.

26. Hackman J: *Groups that work (and those that don't): creating conditions for effective teamwork.* San Francisco: Jossey Bass; 1990.

27. Hackman JR: **The design of work teams.** In *Handbook of organizational behavior.* Edited by Lorsch JW. Englewood Cliffs, NJ: Prentice-Hall; 1987: 315-342

28. Anderson N, West M: **Measuring climate for work group innovation: Development and validation of the Team Climate Inventory.** *J Organ Behav* 1998, **19:**235-258.

29. Janssen O, Van de Vliert E, Veenstra C: **How task and person conflict shape the role of positive interdependence in management teams.** *J Manage* 1999, **25:**117-142.

30. Savelsbergh CM, van der Heijden BI, Poell RF: **The development and empirical validation of a multidimensional measurement instrument for team learning behaviors.** *Small Gr Res* 2009, **40:**578-607.

31. Allen JM, Schumacker RE: **Team assessment utilizing a many-facet Rasch model.** *J Outcome Meas* 1998, **2:**142-158.

32. Caldwell DF, O'Reilly CA, III: **The determinants of team-based innovation in organizations. The role of social influence.** *Small Gr Res* 2003, **34:**497-517.

33. Costa AC, Anderson N: **Measuring trust in teams: Development and validation of a multifaceted measure of formative and reflective indicators of team trust.** *Eur J Work Org Pscychol* 2011, **20:**119-154.

34. Barczak G, Lassk F, Mulki J: **Antecedents of team creativity: An examination of team emotional intelligence, team trust and collaborative culture.** *Creat Innov Man* 2010, **19:**332-345.

35. Bishop JW, Dow Scott K: **An examination of organizational and team commitment in a self-directed team environment.** *J Appl Psychol* 2000, **85:**439-450.

36. Bishop JW, Scott K, Goldsby MG, Cropanzano R: **A Construct Validity Study of Commitment and Perceived Support Variables: A Multifoci Approach Across Different Team Environments.** *Group Organ Manage* 2005, **30:**153-180.

37. Drach-Zahavy A, Freund A: **Team effectiveness under stress: A structural contingency approach.** *J Organ Behav* 2007, **28:**423-450.

38. Han G, Harms P: **Team identification, trust and conflict: A mediation model.** *Int J Confl Manage* 2010, **21:**20-43.

39. van Der Vegt G, van De Vliert E, Oosterhof A: **Informational dissimilarity and organizational citizenship behavior: The role of intrateam interdependence and team identification.** *Acad Manage J* 2003, **46:**715-727.

40. Allen NJ, Meyer JP: **The measurement and antecedents of affective, continuance and normative commitment to the organization.** *J Occup Psychol* 1990, **63:**1-18.

41. Edmondson A: **Psychological safety and learning behavior in work teams.** *Adm Sci Q* 1999, **44:**350-383.

42. Campion MA, Medsker GJ, Higgs AC: **Relations between work group characteristics and effectiveness: Implications for designing effective work groups.** *Pers Psychol* 1993, **46:**823-850.

43. Brav A, Andersson K, Lantz A: **Group initiative and self-organizational activities in industrial work groups.** *Eur J Work Org Pscychol* 2009, **18:**347-377.
44. West MA, Hirst G, Richter A, Shipton H: **Twelve steps to heaven: Successfully managing change through developing innovative teams.** *Eur J Work Org Pscychol* 2004, **13:**269-299.
45. Bunderson JS, Sutcliffe KM: **Management team learning orientation and business unit performance.** *J Appl Psychol* 2003, **88:**552-560.
46. Hoegl M, Gemuenden HG: **Teamwork quality and the success of innovative projects: A theoretical concept and empirical evidence.** *Organ Sci* 2001, **12:**435-449.
47. van den Bossche P, Gijselaers WH, Segers M, Kirschner PA: **Social and Cognitive Factors Driving Teamwork in Collaborative Learning Environments: Team Learning Beliefs and Behaviors.** *Small Gr Res* 2006, **37:**490-521.
48. Bunderson J, Boumgarden P: **Structure and learning in self-managed teams: Why "bureaucratic" teams can be better learners.** *Organ Sci* 2010, **21:**609-624.
49. Carless SA, De Paola C: **The measurement of cohesion in work teams.** *Small Gr Res* 2000, **31:**71-88.
50. Zaccaro SJ: **Nonequivalent associations between forms of cohesiveness and group-related outcomes: Evidence for multidimensionality. Jun 1991 1991.** *J Soc Psychol* 1991, **131:**387-399.
51. Avolio BJ, Bass BM, Jung DI: **Re-examining the components of transformational and transactional leadership using the Multifactor Leadership Questionnaire.** *J Occup Organ Psychol* 1999, **72:**441–462.
52. Kouzes JM, Posner BZ: *Leadership practices inventory [LPI]: facilitator's guide.* 3rd edn. San Francisco: Pfeiffer & Co; 2003.
53. Carless SA, Wearing AJ, Mann L: **A short measure of transformational leadership.** *J Bus Psychol* 2000, **14:**389-405.
54. Nielsen K, Yarker J, Randall R, Munir F: **The mediating effects of team and self-efficacy on the relationship between transformational leadership, and job satisfaction and psychological well-being in healthcare professionals: a cross-sectional questionnaire survey.** *Int J Nurs Stud* 2009, **46:**1236-1244.
55. Podsakoff PM, MacKenzie SB, Moorman RH, Fetter R: **Transformational leader behaviors and their effects on followers' trust in leader, satisfaction, and organizational citizenship behaviors.** *Leadership Quart* 1990, **1:**107-142.
56. de Jong B, Elfring T: **How does trust affect the performance of ongoing teams? The mediating role of reflexivity, monitoring, and effort.** *Acad Manage J* 2010, **53:**535-549.

57. Marks MA, Mathieu JE, Zaccaro SJ: **A temporally based framework and taxonomy of team processes.** *Acad Manage Rev* 2001, **26:**356-376.

58. West MA: **Reflexivity and work group effectiveness: A conceptual integration.** In *Handbook of work group psychology.* Edited by West MA. Chichester, UK: John Wiley; 1996: 159-187

59. Coyle-Shapiro JAM, Morrow PC: **The role of individual differences in employee adoption of TQM orientation.** *J Vocat Behav* 2003, **62:**320-340.

60. Cook J, Wall T: **New work attitude measures of trust, organizational commitment and personal need non-fulfilment. Mar 1980 1980.** *J Occup Psychol* 1980, **Vol.53:**pp.

61. Costa AC: **Work team trust and effectiveness.** *Pers Rev* 2003, **32:**605-622.

62. Dobson RT, Stevenson K, Busch A, Scott DJ, Henry C, Wall PA: **A quality improvement activity to promote interprofessional collaboration among health professions students.** *Am J Pharm Educ* 2009, **73:**64.

63. Eby LT, Dobbins GH: **Collectivistic orientation in teams: An individual and group-level analysis.** *J Organ Behav* 1997, **18:**275-295.

64. Guzzo RA, Yost PR, Campbell RJ, Shea GP: **Potency in groups: Articulating a construct.** *Br J Soc Psychol* 1993, **32:**87-106.

65. Gully SM, Incalcaterra KA, Joshi A, Beaubien JM: **A meta-analysis of team-efficacy, potency, and performance: Interdependence and level of analysis as moderators of observed relationships.** *J Appl Psychol* 2002, **87:**819-832.

66. Janssen O, Huang X: **Us and me: Team identification and individual differentiation as complementary drivers of team members' citizenship and creative behaviors.** *J Manage* 2008, **34:**69-88.

67. Van Knippenberg D: **Work motivation and performance: A social identity perspective.** *Appl Psychol Int Rev* 2000, **49:**357-371.

68. Ellemers N, Kortekaas P, Ouwerkerk JW: **Self-categorisation, commitment to the group and group self-esteem as related but distinct aspects of social identity. Mar-May 1999 1999.** *Eur J Soc Psychol* 1999, **Vol.29:**pp.

69. Jehn KA, Greer L, Levine S, Szulanski G: **The effects of conflict types, dimensions, and emergent states on group outcomes.** *Group Decis Negot* 2008, **17:**465–495.

70. Balkundi P, Harrison D: **Ties, leaders, and time in teams: Strong inference about network structure's effects on team viability and performance.** *Acad Manage J* 2006, **49:**49-68.

71. Kirkman BL, Rosen B: **Beyond self-management: antecedents and consequences of team empowerment.** *Acad Manage J* 1999, **42:**58-74.

72. Kirkman BL, Rosen B: **A model of work team empowerment** In *Research in organizational change and development: An annual series featuring advances in theory, methodology, and research. Volume* 10. Edited by Pasmore WA, Woodman RW: Elsevier Science/JAI Press; 1997: xii, 322

73. Manz CC, Sims HP: **Leading workers to lead themselves: The external leadership of self-managing work teams.** *Adm Sci Q* 1987, **32:**106-128.

74. Lukas CV, Mohr DC, Meterko M: **Team effectiveness and organizational context in the implementation of a clinical innovation.** *Qual Manag Health Care* 2009, **18:**25-39.

75. Shortell SM, Marsteller JA, Lin M, Pearson ML, Wu SY, Mendel P, Cretin S, Rosen M: **The role of perceived team effectiveness in improving chronic illness care.** *Med Care* 2004, **42:**1040-1048.

76. Langfred CW: **Autonomy and Performance in Teams: The Multilevel Moderating Effect of Task Interdependence.** *J Manage* 2005, **31:**513-529.

77. Kiggundu MN: **Task interdependence and job design: Test of a theory.** *Organ Behav Hum Perform* 1983, **31:**145-172.

78. Breaugh JA: **The measurement of work autonomy.** *Hum Relat* 1985, **38:**551-579.

79. Manz CC, Sims HP: **Leading workers to lead themselves: The external leadership of self-managing work teams.** *Adm Sci Q* 1987, **32:**106-129.

80. Cohen SG, Chang L, Ledford GE, Jr.: **A hierarchical construction of self-management leadership and its relationship to quality of work life and perceived work group effectiveness.** *Pers Psychol* 1997, **50:**275-308.

81. Cohen SG, Ledford GE, Jr., Spreitzer GM: **A predictive model of self-managing work team effectiveness.** *Hum Relat* 1996, **49:**643-676.

82. Mathieu JE, Gilson LL, Ruddy TM: **Empowerment and team effectiveness: an empirical test of an integrated model.** *J Appl Psychol* 2006, **91:**97-108.

83. Seers A: **Team-member exchange quality: A new construct for role-making research.** *Organ Behav Hum Decis Process* 1989, **43:**118-135.

84. Ford LR, Seers A: **Relational leadership and team climates: Pitting differentiation versus agreement.** *Leadership Quart* 2006, **17:**258-270.

85. Tse HH, Dasborough MT, Ashkanasy NM: **A multi-level analysis of team climate and interpersonal exchange relationships at work.** *Leadership Quart* 2008, **19:**195-211.

86. Driskell JE, Salas E, Hughes S: **Collective orientation and team performance: development of an individual differences measure.** *Hum Factors* 2010, **52:**316-328.

87. Shaw JD, Duffy MK, Stark EM: **Interdependence and preference for group work: Main and congruence effects on the satisfaction and performance of group members.** *J Manage* 2000, **26:**259-279.
88. Wagner JA, Moch MK: **Individualism-collectivism: Concept and measure.** *Group Organ Stud* 1986, **11:**280-303.
89. Staples D, Webster J: **Exploring the effects of trust, task interdependence and virtualness on knowledge sharing in teams.** *Inform Syst J* 2008, **18:**617-640.
90. Nissen ME, Jennex ME: **Knowledge as a multidimensional concept: a call for action.** *Int J Knowl Manage* 2005, **1:**i-v.
91. van der Vegt G, Emans B, van de Vliert E: **Motivating effects of task and outcome interdependence in work teams.** *Group Organ Manage* 1998, **23:**124-143.
92. van der Vegt G, Emans BJ, van de Vliert E: **Patterns of interdependence in work teams: A two-level investigation of the relations with job and team satisfaction.** *Pers Psychol* 2001, **54:**51-69.
93. van der Vegt GS, van de Vliert E: **Effects of Perceived Skill Dissimilarity and Task Interdependence on Helping in Work Teams.** *J Manage* 2005, **31:**73-89.
94. Hackman JR, Oldham GR: **Motivation through the design of work: Test of a theory.** *Organ Behav Hum Perform* 1976, **Vol.16**.
