# Supplementary material for: Measuring team factors thought to influence the success of quality improvement in primary care: a systematic review of instruments
Source: Implement Sci. 2013 Feb 14;8:20. doi: 10.1186/1748-5908-8-20 (PMC3602018; doi:10.1186/1748-5908-8-20)
Supplement: Additional file 7 — Table summarizing the development and measurement properties of instruments included in Stage four of the review (Table S9). [file 1748-5908-8-20-S7.pdf]

Additional file 7

Table S9: Summary of instrument development and assessments of measurement properties (Stage four)

**Instrument**<br>(name, index, references for main reports)	**Development & content validity**<br>(item generation; content validity assessments)	**Construct validity**	**Reliability**	**Other assessments**<br>(eg feasibility, acceptability, interpretability)
Primary care settings				
Team climate for innovation inventory (TCI)<br><br>Anderson 1998 [1-2]	Scales and items from published measure of climate were examined for coverage of the four dimensions of the conceptual model and consistency with team level measurement. New items were written for most scales. Factor analysis was used to refine the scales.<br><br>Content validity: face validity and acceptability assessed from comments from pilot testing with 16 nursing and hospital management teams.<br><br>Initial testing with hospital management teams (RR 155/243, 64% from 27 teams). Confirmatory factor analysis with teams from various settings (35/121 teams from primary care). Short form developed from data from social service and healthcare teams (RR 52%, sample 1=1494 and 2=771) [2].	Hypothesis testing (examples from primary care): Association between scale scores and i) quality of care indicators [3-7], ii) patient assessment of care [3, 7-8], iii) perceived team effectiveness [3, 9], iv) innovation quality in primary care teams [10], v) quality of work life indicators [11-12].<br><br>Structure: Four factors predicted from conceptual model, five were empirically supported (exploratory and confirmatory factor analysis). Three factors were comprised of items from scales as predicted (vision, innovation, task orientation); items from the participative safety scale formed two factors.	Internal consistency: Cronbach's alpha for the five scales ranged from 0.84 to 0.94 [1] with similar findings in primary care teams (e.g. [13]).<br><br>Stability over time: scale scores and factor structure were stable over two administrations [14]<br><br>Inter-rater reliability: intraclass correlation coefficients (ICCs) indicate acceptable within-group agreement and ANOVA confirms that differences between groups can be discriminated [1].	Short form exhibits comparable properties to long form; factor structure is similar, items load on factors expected and content of scales covers main elements of constructs; long and short form scales correlate with each other as expected, and both forms appear to predict innovativeness [2]. Short form version has been used in primary care (e.g. [13])<br><br>Versions available in multiple languages.
Team culture questionnaire<br><br>Hearnshaw 1998 [15]	Methods of item generation not reported. One source reference was provided [16]. Unclear whether this is a source reference for the instrument.<br><br>Content validity: no independent assessments reported<br><br>Administered to members of primary care practices participating in a total quality management program (RR 82 members from 6/6 practices. Individual RR not reported).	Hypothesis testing: none reported<br><br>Structure: not assessed	Internal consistency: not reported<br><br>Stability over time: not assessed<br><br>Inter-rater reliability: not assessed	The authors report mean scale scores, but not standard deviations, for each practice.<br><br>No other assessments reported.
Working as a team survey<br><br>Lukas 2009 [17]	Items were adapted from two instruments for assessing team effectiveness [18-19]<br><br>Content validity: no independent assessments reported.<br><br>Administered post-intervention to clinical and non-clinical staff participating in an evaluation of a national QI initiative in primary care and specialist clinics in the Department of Veterans Affairs. (RR 3870/9053, 42% of staff from 257 clinics, 74 of which were primary care)	Hypothesis testing: i) association between team effectiveness (knowledge and skills, functioning, problem recognition) and implementation of the QI intervention [17, 20], ii) team effectiveness as a mediator of the effect of leadership on implementation. Mixed support for hypotheses.<br><br>Structure: Four factors were identified (exploratory factor analysis), three measuring team effectiveness, and one measuring management support.	Internal consistency: Cronbach's alpha for the four scales ranged from 0.71 to 0.92.<br><br>Stability over time: not assessed<br><br>Inter-rater reliability: not assessed (hierarchical linear modelling was used for hypothesis testing, accounting for clustering).	No other assessments reported

**Instrument**<br>(name, index, references for main reports)	**Development & content validity**<br>(item generation; content validity assessments)	**Construct validity**	**Reliability**	**Other assessments**<br>(eg feasibility, acceptability, interpretability)
Perceived team effectiveness survey<br><br>Shortell 2004 [19, 21]	The instrument is an adaptation of Lemieux-Charles 2002 [18]. Rationale for changes not reported.<br><br>Content validity: No independent assessments reported.<br><br>Administered post-intervention to team members participating in an evaluation of national QICs to improve chronic illness care (261 members from 40 QI teams provided data for analyses; RR not reported).	Hypothesis testing: Association between team effectiveness (overall and subscales) and i) presence of a team champion, ii) organisational culture, iii) organisational commitment to QI and iv) number and depth of changes made to care processes. Most hypotheses supported.<br><br>Structure: Factor analysis was performed to ensure items loaded onto scales as predicted. Results not reported.	Internal consistency: Cronbach's alpha for the four scales ranged from 0.85 to 0.95<br><br>Stability over time: not assessed<br><br>Inter-rater reliability: ANOVA indicated greater between team than within team variation, supporting team level measurement.	No other assessments reported.<br><br>Individual level responses were aggregated to team level based on ANOVA (methods and results not reported).
Organizational assessment measure<br><br>Shortell, 1991 [22-23]	Constructs and dimensions based on literature review of practices and processes key to organisational effectiveness. Items were written for each identified factor. Some items were derived from existing scales. Items from four scales in the original instrument were later adapted for primary care [23].<br><br>Content validity: Instrument pilot-tested with ICU nurses and physicians (n=187). Based on findings, a second profession-specific version was written and some items revised for clarity. No independent assessments of content reported.<br><br>Initial testing with ICU staff (1418 returned, RR 73%) [22]. Primary care version administered to all clinicians and staff in practices participating in randomised trial (RR 353/420, 84% from all 36 practices) [23].	Hypothesis testing: Association between scale scores and outcomes: i) perceived technical quality of care, ii) unit ability to meet family member needs, and iii) nursing turnover. Association between scale scores. Most hypotheses supported. Multiple hypotheses subsequently tested in primary care sample [24-25].<br><br>Structure: Subscales for each of the constructs were identified using exploratory factor analysis. Most theoretically predicted scales in the primary care version were empirically supported (principal components analysis) [23].	Internal consistency: Cronbach's alphas for scale scores ranged from 0.64 to 0.88. Primary care version, 0.70 to 0.93.<br><br>Stability over time: not assessed<br><br>Inter-rater reliability: ANOVA indicated greater between team than within team variation for all scales, supporting team level measurement.	Feasibility: completion time for short form of ICU version approximately 20 minutes.
Other health care settings				
Group innovation inventory<br><br>Caldwell 2003 [26-27]	Items written by investigators based on a list of norms and beliefs that promote innovation in groups elicited from discussion with participants in executive training programs.<br><br>Content validity: no independent assessments reported<br><br>Initial administration to postgraduate business students (147 students representing 147 teams; plus 97/150 team members from 30 of these teams 65%). Subsequent testing and development of short form using data from teams participating in a QIC to improve home-based care (n=261 (baseline), n=129 (follow up). RR not reported) [27].	Hypothesis testing: Association between scale scores and i) self-reported innovation [26] and ii) score on team climate inventory scales [27]. Inter-correlation between scale scores. Mixed support for hypotheses, some scales scores did not correlate as expected.<br><br>Structure: Four factors were identified (principal components analysis) [26] and confirmed in a subsequent study (confirmatory factor analysis) [27].	Internal consistency: Cronbach's alpha for four scales ranged from 0.45 to 0.79 [27].<br><br>Stability over time: not assessed<br><br>Inter-rater reliability: not assessed (most analyses performed on individual level data)	Strating and colleagues report a short form of the instrument, which correlated as expected with the original scales. Item wording and length appear more suitable for healthcare context [27].

**Instrument** (name, index, references for main reports)	**Development & content validity** (item generation; content validity assessments)	**Construct validity**	**Reliability**	**Other assessments** (eg feasibility, acceptability, interpretability)
Work group characteristics measure<br><br>Campion 1993 [28]	Items were written to operationalise 19 work group characteristics across five themes in a conceptual model developed from literature review.<br><br>Content validity: no independent assessments reported<br><br>Administered to members of financial services teams (RR study 1: 391/400, from 80 groups and 77/80 group managers [28]. Study 2: 357 from 60 groups, 64% [29]).	Hypothesis testing: Association between scale scores and team performance measured by i) productivity over 6 months (routine data), ii) employee satisfaction (routine data), iii) manager rating of group effectiveness (4 item survey). Inter-correlation between scale scores [28-29]. Mixed support for hypotheses.<br><br>Structure: Most items loaded on scales as predicted (exploratory factor analysis), providing empirical support for the 19 dimensions. Replication in a separate sample [29] using 16 scales produced similar findings and largely supported the five categories in the conceptual framework.	Internal consistency: Cronbach's alpha for 19 scales ranged from 0.47 to 0.90 (five scales <0.5). In subsequent study, Cronbach's alpha >0.7 for all 16 scales (2 scales dropped, 1 single item).<br><br>Stability over time: not assessed<br><br>Inter-rater reliability: correlation of scale scores between team members was 'modest' and inter-rater agreement was 'moderate to high' (Campion 1993 p835).	No other assessments reported.<br><br>Individual scales have been widely used in other studies (for examples see [30-32]), although very few examples were identified in health care and none in primary care.
Team trust<br><br>Costa 2011 [33]	For each of the hypothesised dimensions, new items were generated or items from existing measures of team trust were adapted (following extensive literature review).<br><br>Content validity: Subject matter experts reviewed items for comprehensibility, length and singularity, then for i) relevance and coverage of the four hypothesised dimensions, ii) relevance to work teams, iii) redundancy of content.<br><br>First administration to hospital teams (98 members from 14 teams, RR 48%); second administration to social care teams (395 members from 112 teams, RR 71%)	Hypothesis testing: Association between scale scores and scores on team commitment and organisational commitment scales. Most hypotheses supported.<br><br>Structure: Four theoretical dimensions were predicted, all four were empirically supported (exploratory and confirmatory factor analysis on two samples).	Internal consistency: Cronbach's alpha for four scales ranged from 0.70 to 0.88<br><br>Stability over time: not assessed<br><br>Inter-rater reliability: r_{wg} and ANOVA indicated acceptable within group agreement and between group differences; intraclass correlations indicated that most variance was accounted for at team level and supported the reliability of the measure at team level	No other assessments reported.
Knowledge, attitudes and beliefs relating to inter-professional teams<br><br>Dobson 2009 [34-35]	Items generated from definitions of key dimensions of teamwork derived from a literature review [35].<br><br>Content validity: the original version of the instrument was reviewed by members of the same teaching faculty for appropriateness of wording and content [35].<br><br>Administered pre- and post intervention to healthcare students participating in an inter-professional QI project (RR 134/223 team members 76%).	Hypothesis testing: Differences between scores pre and post participation in an intervention to promote inter-professional competencies.<br><br>Structure: not assessed	Internal consistency: not assessed<br><br>Stability over time: not assessed<br><br>Inter-rater reliability: not assessed	No other assessments reported.
Group process<br><br>Dobson 2009 [34]	Methods of item generation not reported.<br><br>Content validity: no independent assessments reported.<br><br>Administered post intervention to healthcare students participating in an inter-professional QI project (RR 132/223 team members 74%).	Hypothesis testing: none reported.<br><br>Structure: not assessed	Internal consistency: not assessed<br><br>Stability over time: not assessed<br><br>Inter-rater reliability: not assessed	Post-intervention means and standard deviations reported for each of the items.<br><br>No other assessments reported.

**Instrument**<br>(name, index, references for main reports)	**Development & content validity**<br>(item generation; content validity assessments)	**Construct validity**	**Reliability**	**Other assessments**<br>(eg feasibility, acceptability, interpretability)
Team learning behaviour<br><br>Edmondson 1999 [36]	Items were written to operationalise constructs in a theoretical model of team learning [36], using definitions from published theory and qualitative data to ensure appropriate language for context of use.<br><br>Content validity: no independent assessments reported.<br><br>Administered to team members in a manufacturing firm (RR 427/496, 86% of members 51/53 teams. RR per team 90%).	Hypothesis testing: Associations between team beliefs (psychological safety, efficacy), context support, team learning behaviour, and team performance (customer satisfaction) [36]. Most hypotheses supported.<br><br>Structure: A single scale was empirically supported (principal components analysis), with items loading as predicted.	Internal consistency: Cronbach's alpha for the learning behaviour scale was 0.78.<br><br>Stability over time: not assessed<br><br>Inter-rater reliability: The scale had a high ICC, indicating acceptable within-group agreement.	No other assessments reported<br><br>Scores from self-report learning behaviour scale and a behavioural observation scale correlated as expected.
Team shared beliefs<br><br>Edmondson 1999 [36]	Items were written to operationalise constructs in a theoretical model of team learning [36], using definitions from published theory and qualitative data to ensure appropriate language for context of use.<br><br>Content validity: no independent assessments reported.<br><br>Administered to team members in a manufacturing firm (RR 427/496, 86% of members 51/53 teams. RR per team 90%). Subsequent administration of psychological safety scale to QI teams in hospitals (RR study 1: 1440/3130, 46% of members from 23/23 units; study 2: 49/76 team members, 64% from 8 CQI teams) [37-38].	Hypothesis testing: Associations between team beliefs (psychological safety, efficacy), context support, team learning behaviour, and team performance (customer satisfaction) [36]. Associations between professional status, leader inclusiveness, psychological safety and QI participation [37]. Most hypotheses supported.<br><br>Structure: The two constructs were empirically supported (principal components analysis), with items loading on scales as predicted.	Internal consistency: Cronbach's alpha for the psychological safety and efficacy scales was 0.82 and 0.63 respectively.<br><br>Stability over time: not assessed<br><br>Inter-rater reliability: Both scales had high ICCs, indicating acceptable within-group agreement.	No other assessments reported
Teamwork context and internal team management<br><br>Lemieux-Charles 2002a [18]	Items were written to operationalise factors thought to influence QI team effectiveness as identified from on selected literature. Methods of item generation not reported.<br><br>Content validity: no independent assessments reported<br><br>Administered to QI teams from acute care hospitals (RR 506/732, 69% of team members from 97 teams. Data from 79 teams available for analysis)	Hypothesis testing: Association between context and i) internal team management (norms, process strategies, decision-making) and ii) QI practices. Internal team management as a mediator of the effect of QI practices and context on perceived team effectiveness (self-rated; externally rated). Mixed support for hypotheses.<br><br>Structure: Four theoretically meaningful dimensions related to teamwork and context were identified (exploratory factor analysis).	Internal consistency: Cronbach's alpha for the four scales ranged from 0.78 to 0.92.<br><br>Stability over time: not assessed<br><br>Inter-rater reliability: average deviation index calculated for each of the scales confirmed that individual scores could be aggregated to team level. For all four scales, about 90% of individual scores were below the cutoff for aggregation.	No other assessments reported.<br><br>See also, adaptations of this measure in Shortell 2004 [19] and Lukas 2009 [17].
Perceived team effectiveness<br><br>Lemieux-Charles 2002b [18]	Items were written to operationalised Hackman's definition of team effectiveness [39].<br><br>Content validity: no independent assessments reported<br><br>Self-report measure administered to QI teams from acute care hospitals (RR 506/732, 69% of team members from 97 teams. Data from 79 teams available for analysis)	Hypothesis testing: Association between perceived team effectiveness (self-rated; externally rated) and i) use of QI practices, ii) context, and iii) internal team management (norms, process strategies, decision-making). Correlation between self-rated effectiveness and an external (manager) rating at two times points. Mixed support for hypotheses.<br><br>Structure: not assessed	Internal consistency: Cronbach's alpha for the team member rated scale was 0.93.<br><br>Stability over time: not assessed<br><br>Inter-rater reliability: not assessed	No other assessments reported.

Instrument (name, index, references for main reports)	Development & content validity (item generation; content validity assessments)	Construct validity	Reliability	Other assessments (eg feasibility, acceptability, interpretability)
Transactive memory system (TMS) scale<br><br>Lewis 2003 [40]	Items were written for each of three manifestations of transactive memory, based on conceptual definitions from theoretical and empirical research.<br><br>Content validity: Content experts reviewed the items for clarity and appropriateness, resulting in rewording of one item.<br><br>Administered to three samples: (i) 372 members of 34 student teams, (ii) 260 members of 64 student teams (iii) 146/158 members of 27 managerial teams. French version [41] administered to anaesthesiology teams to test association with outcome measures (RR 151/193, 78%) [42].	Hypothesis testing: Association between scale scores and (i) observer ratings of TMS, (ii) scale score for constructs with which the measure is expected to converge (communication) and discriminate (motivation, cohesion, autonomy), (iii) outcome measures (perceived team performance, team identification, job satisfaction). Difference between groups (trained groups compared to untrained). Most hypotheses supported.<br><br>Structure: Three theoretical dimensions were predicted and empirically supported (confirmatory factor analysis, samples 1 and 2).	Internal consistency: Cronbach's alpha for the three scales ranged from 0.72 to 0.91.<br><br>Stability over time: not assessed<br><br>Inter-rater reliability: intra-group agreement (r_{wg}) was assessed for all three scales, and indicated acceptable within-group agreement.	No other assessments reported.
Perceived team integration<br><br>Lichtenstein 1997 [43]	Items were adapted from existing instruments (e.g. [39]). The rationale and nature of the changes to the scales were not described.<br><br>Content validity: no independent assessments reported<br><br>Administered to interdisciplinary treatment teams participating in a QI initiative in psychiatric hospitals (RR 1746/1801, 97%; analytical sample 1004 individuals from 105 teams). Subset of data (from 40 teams) used in subsequently published analyses [44].	Hypothesis testing: Association between perceptions of team integration and i) team member diversity (age, sex, professional role, role tenure, organisational tenure), ii) team size [43] and iii) patient functional status [44]. Mixed support for hypotheses. Scale correlated with each other as predicted.<br><br>Structure: Three factors were identified (principal components analysis), forming theoretically meaningful scales.	Internal consistency: Cronbach's alpha for the three scales ranged from 0.90 to 0.91.<br><br>Stability over time: not assessed<br><br>Inter-rater reliability: correlation of scale scores between team members was high, indicating acceptable within group agreement [44].	Interclass correlations indicated that two of the scales (role clarity, team functioning) were meaningful as group level constructs, while the third scale (participation) reflected an individual level construct [43].<br><br>No other assessments reported.
Team check-up tool (TCT)<br><br>Lubomski 2008 [45-46]	Methods of item generation not reported.<br><br>Content validity: no independent assessments reported.<br><br>Administered every month for 12 months to team members participating in a RCT of a QI intervention in 46 hospital ICUs. (RR varied, on average 51% of ICUs submitted ≥ one TCT per month over the first 12 months). Data available for analyses ranged from 31 ICUs for reliability analyses to 15 ICUs for tests of predictive and discriminant validity.	Hypothesis testing: Association between barriers to team progress and i) infection prevention behaviour scale, and ii) scale scores for measures of unrelated and related constructs (team function scale [19]). Whether barriers are predictive of central line-associated bloodstream infection. Mixed support for hypotheses.<br><br>Structure: not assessed. Items relating to infection prevention behaviour and barriers expected to form a scale.	Internal consistency: Cronbach's alpha for barriers scale 0.91 and for infection prevention behaviour scale 0.78.<br><br>Stability over time: ICU level agreement statistic calculated from two consecutive administrations of scales during a period when scores were not expected to change. Most barriers items (10 of 13) demonstrated moderate correlation between the two administrations.	Responsiveness: Scores changed in expected direction during periods in which they were expected change.<br><br>Acceptability: respondents reported completion time of 7-10 minutes<br><br>The five items that focus on team function are not included in any of the assessments. Hence, there are no data to assess the measurement properties of these items.

**Instrument** (name, index, references for main reports)	**Development & content validity** (item generation; content validity assessments)	**Construct validity**	**Reliability**	**Other assessments** (eg feasibility, acceptability, interpretability)
Team characteristics questionnaire<br><br>Mills 2004; Weeks 2001 [47-48]	Items based on microsystem and teamwork literature. Methods of item development not reported.<br><br>Content validity: no independent assessments reported<br><br>Administered to teams participating in five QICs in the Veterans' Health Administration, most in hospital settings (RR 134/144 teams, 93%). QICs focussed on i) adverse drug events, ii) safety in high risk areas, iii) home-based dementia care, iv) pension and compensation processing, v) falls and injury prevention.	Hypothesis testing: Difference between successful and unsuccessful teams on scores on individual items and overall scales (teams categorised based on predefined criteria for successful improvement of process and clinical outcomes). Some differences between teams for individual items, but not overall scales. Limited support for hypotheses.<br><br>Structure: Four dimensions were identified (principal components analysis), with the aim of controlling for inter-correlations between items.	Internal consistency: Cronbach's alpha for the four scales was not reported.<br><br>Stability over time: not assessed<br><br>Inter-rater reliability: not assessed	No other assessments reported.<br><br>The assessments summarised here are primarily from the analysis of aggregated data reported in Mills 2004 [47]. Individual studies contributing data to these analyses are listed in table S7.
The team survey<br><br>Millward 2001 [49]	Items were derived from existing scales, some of which were used in a version of the team survey designed for industrial settings. Content and items were refined based on focus group discussion with health professionals.<br><br>Content validity: no independent assessments reported. Unclear whether focus groups were used to elicit feedback on instrument content or more general information about healthcare teamwork.<br><br>Administered to healthcare teams in UK National Health Service Trust. Unclear if primary care teams were included. (RR 99/124, 79.8% of members from 10 teams).	Hypothesis testing: i) association between scale scores and independently rated team performance and ii) inter-correlations between scales on the instrument. Limited support for association between scale scores and performance. Scales correlated with each other, one negatively with other scales.<br><br>Structure: Seven theoretical dimensions were predicted. Five factors were empirically supported (confirmatory factor analysis), only four of which were theoretically meaningful and items comprising scales were not as predicted.	Internal consistency: Cronbach's alpha for the four scales ranged from 0.73 to 0.93.<br><br>Stability over time: not assessed<br><br>Inter-rater reliability: correlation between team member ratings (using Split-Half method) indicated consistency between team members' scores.	No other assessments reported.<br><br>Sample size is small, particularly for factor analysis and hypothesis testing (the latter, performed on scores aggregated to group level) hence, results are preliminary.
Factors influencing success in a QIC<br><br>Schouten, 2010 [50]	Items were written to operationalised determinants of QIC success identified from a systematic review of QIC theory. Overlapping items were combined, and vague, ambiguous or redundant items were removed.<br><br>Content validity: content experts reviewed items for "readability, comprehensibility, ease of response, content validity" (p2). Items were accepted or deleted based on expert agreement.<br><br>Initial testing in a sample of QIC participants (RR 44/46 teams, 95%)	Hypothesis testing: Correlation between scales. All hypotheses supported.<br><br>Structure: Four theoretical dimensions were predicted, one of which was multidisciplinary teamwork. Items from this scale and 'use of the improvement model' loaded on the same factor (factors identified by principal component analysis). The resulting scale contained items deemed to be a theoretically meaningful measure of teamwork.	Internal consistency: Cronbach's alpha for the single scale was 0.89.<br><br>Stability over time: not assessed.<br><br>Inter-rater reliability: not assessed (unclear whether analyses on individual or team level data)	No other assessments reported.
Team performance survey (TPS)<br><br>Thompson 2009 [51]	Items were based on selected literature on team based learning and discussion among the authors. Factor analysis was used to refine the scale.<br><br>Content validity: no independent assessments reported.<br><br>Administered to undergraduate medical students enrolled in a course using team-based learning methods (RR 309/325, members from 60 teams 95%).	Hypothesis testing: Association between scores on the TPS and variability in peer-ratings of team member performance. Hypothesis supported.<br><br>Structure: No a priori hypotheses about the number of constructs or dimensions measured by the instrument. Items formed a single scale (exploratory factor analysis).	Internal consistency: Cronbach's alpha for the scale was 0.97.<br><br>Stability over time: not assessed<br><br>Inter-rater reliability: not assessed	Respondents reported that the scale could be completed in five minutes.

**Instrument** (name, index, references for main reports)	**Development & content validity** (item generation; content validity assessments)	**Construct validity**	**Reliability**	**Other assessments** (eg feasibility, acceptability, interpretability)
CQI team climate and interpersonal relationships<br><br>Wilkens 2006 [38]	Measure is comprised of two new scales and five adaptations of existing scales [36, 52-56]. Methods of item development for new scales not reported and adaptations of existing scales not described.<br><br>Content validity: no independent assessments reported.<br><br>Administered to members of CQI teams in a university hospital (RR 49/76 members from 8 CQI teams, 64%).	Hypothesis testing: Association between team climate (three subscales), interpersonal relationships (four subscales) and self-rated team performance and creativity. Difference between high and low performing teams (based on rating from interviews with team facilitator). Mixed support for hypotheses.<br><br>Structure: Factor analysis not performed. Correlation between scales was examined. Climate and interpersonal subscales correlated as predicted.	Internal consistency: Cronbach's alpha for seven scales ranged from 0.68 to 0.95.<br><br>Stability over time: not assessed<br><br>Inter-rater reliability: not assessed	No other assessments reported.
Non health care				
Empowering leadership questionnaire (ELQ)<br><br>Arnold 2000 [57]	Empowering leadership behaviours were elicited in interviews with team leaders and members. Content analysis used to identify and categorise behaviours and determine item content. Inter-item and item-total correlation and CFA were used to refine the instrument.<br><br>Content validity: the investigators removed items that were complex, ambiguous or did not describe the behaviours.<br><br>Initial administration to team members in manufacturing and customer service (n=205, RR not reported). Validation study and testing with data from members of healthcare, finance and food processing teams (n=374, RR not reported).	Hypothesis testing: Association between scale scores and scores on two other instruments measuring leader behaviours (Managerial Practices Survey and Considering and subscales from Leader Behaviour Description Questionnaire). Scale scores all highly correlated.<br><br>Structure: Eight theoretical dimensions were predicted. Five factors were empirically supported (confirmatory factor analysis on data from two samples) and theoretically meaningful.	Internal consistency: Cronbach's alpha for the five scales ranged from 0.89 to 0.93.<br><br>Stability over time: not assessed<br><br>Inter-rater reliability: not assessed	No other assessments reported.
Team learning orientation<br><br>Bunderson 2003 [52]	Items based on an previously reported instrument designed to measure individual learning orientation [55, 58].<br><br>Content validity: no independent assessments reported.<br><br>Administered to i) business management teams (RR 438 members from 44/45 teams, 84%) [52]; ii) members of manufacturing teams (RR 228/369 members from 40/44 teams, 90% provided sufficient data for the analysis) [59].	Hypothesis testing: Association between scale score and i) team structure), ii) team process (information sharing, psychological safety, conflict frequency) [59]. Whether scale scores are predictive of objective performance indicators (measured 12 months post scale administration) [52]. Most hypotheses supported.<br><br>Structure: not assessed for five item scale.	Internal consistency: Cronbach's alpha for the single scale was 0.92.<br><br>Stability over time: not assessed<br><br>Inter-rater reliability: r_{wg} and ANOVA indicated acceptable within group agreement and between group differences; intraclass correlations indicated that most variance was accounted for at team level and supported the reliability of the measure at team level.	No other assessments reported.

**Instrument** (name, index, references for main reports)	**Development & content validity** (item generation; content validity assessments)	**Construct validity**	**Reliability**	**Other assessments** (eg feasibility, acceptability, interpretability)
Team structure<br><br>Bunderson 2010 [59]	Methods of item generation not reported. Items were based on a definition of team structure from conceptualisations in the sociological literature.<br><br>Content validity: no independent assessments reported.<br><br>Administered to members of manufacturing teams (RR 228/369 members from 40/44 teams, 90% provided sufficient data for the analysis).	Hypothesis testing: Association between scale score i) team process (information sharing, psychological safety, conflict frequency), ii) team learning orientation (equally weighted rating by team and supervisions). Most hypotheses supported.<br><br>Structure: a single factor was empirically supported (confirmatory factor analysis).	Internal consistency: Cronbach's alpha for the single scale was 0.75.<br><br>Stability over time: not assessed<br><br>Inter-rater reliability: r_{wg} and ANOVA indicated acceptable within group agreement and between group differences; intraclass correlations indicated that most variance was accounted for at team level and supported the reliability of the measure at team level.	No other assessments reported.
Teamwork and trust in colleagues<br><br>Coyle-Shapiro, 2003 [60]	Teamwork items based on selected literature. Methods of item generation not reported. Trust items from an existing scale [61].<br><br>Content validity: no independent assessments reported.<br><br>Administered pre- and post- implementation of TQM in an automotive supply company (RR pre 186/200, 93%; 9 months post 166/186, 89%; 32 months post 118/186, 71%)	Hypothesis tested: Association between TQM orientation (teamwork dimension) and potential predictors of TQM orientation: i) individual factors (organisational commitment, trust in colleagues, higher order need strength) and ii) organisation level factors (management support, supervisor support). Mixed support for hypotheses.<br><br>Structure: Teamwork and trust items formed two separate scale (principal components analysis), with items loading as predicted.	Internal consistency: Cronbach's alpha for the single teamwork scale 0.90 and for the trust scale 0.86.<br><br>Stability: not assessed<br><br>Inter-rater reliability: not assessed for teamwork scale. Trust was conceptualised as an individual level measure.	No other assessments reported.
Collective orientation scale<br><br>Driskell 2010 [62]	An initial pool of items was generated following review of theory, empirical research and existing measures of cooperation and interdependence. Inter-item correlations were used to remove poorly correlating items and those with poor ability to discriminate between respondents.<br><br>Content validity: no independent assessments reported.<br><br>Administered to undergraduate students and armed services (3 samples, 2 to refine instrument and the 3rd for factor analysis. different analyses. RR X/Y, Z%).	Hypothesis testing: Association between scales scores and scores on i) 8 scales with which positive, negative or no correlation was predicted, ii) scores on four types of team tasks (outcome variables). Most hypotheses supported.<br><br>Structure: Two dimensions were empirically supported (exploratory factor analysis)	Internal consistency: Cronbach's alpha for the two scales was 0.85 and 0.75.<br><br>Stability over time: not assessed<br><br>Inter-rater reliability: scores were aggregated to team level for hypothesis testing (ii), but inter-rater reliability was not reported.	No other assessments reported.
Team context<br><br>Edmondson 1999 [36]	Two existing scales used [63] and new items written for team composition, clear direction and task design scales.<br><br>Content validity: no independent assessments reported.<br><br>Administered to team members in a manufacturing firm (RR 427/496, 86% of members 51/53 teams. RR per team 90%).	Hypothesis testing: Association between context support and coaching and i) team beliefs, ii) and team learning behaviour. Most hypotheses supported. Scores from self-report scales and independent rating based on analysis of interview data were correlated.<br><br>Structure: Three of four constructs were empirically supported (principal components analysis), with items loading on scales as predicted (context items loaded on two factors). Items from the composition scale loaded onto other scales.	Internal consistency: Cronbach's alpha for context and coaching scales was 0.65 and 0.80 respectively. Not reported for other scales.<br><br>Stability over time: not assessed	No other assessments reported<br><br>Scales measuring task design, clear direction and team composition were used to provide feedback to teams, but were not tested in the model of team learning.

Instrument (name, index, references for main reports)	Development & content validity (item generation; content validity assessments)	Construct validity	Reliability	Other assessments (eg feasibility, acceptability, interpretability)
Collective leadership scales<br><br>Hiller 2006 [64]	Items were written to measure behaviours relevant to collective leadership, as identified from an existing instrument [65]<br><br>Content validity: Subject matter experts reviewed items for relevance to the construct and each of the hypothesised dimensions. Items for which there was poor agreement were removed.<br><br>Administered to members of road maintenance teams (RR not reported. Team excluded if <50% of members responded. 277 members from 52 teams included in analysis, with an average response rate from these teams of 74%).	Hypothesis testing: Association between scale scores and i) team performance (supervisor rated), ii) predictors of collective leadership. Mixed support for hypotheses.<br><br>Structure: Four theoretical dimensions were predicted, all of which were empirically supported (confirmatory factor analysis) with most items loading as expected.	Internal consistency: Cronbach's alpha for the four scales ranged from 0.92 to 0.96<br><br>Stability over time: not assessed<br><br>Inter-rater reliability: equivocal support for the reliability of the scales at team level, results suggesting that there may not be acceptable within group agreement (based on r_{wg}) or consistency of responses (based on ICC1).	No other assessments reported.
Teamwork quality scales<br><br>Hoegl 2001 [66]	Methods of item generation not reported. Some items measuring communication and mutual support were from an existing instrument [67].<br><br>Content validity: No independent assessments reported. Item wording was refined based on pre-testing with teams.<br><br>Administered to members of software development teams (RR not reported. Data from 285 members of 145 teams, with corresponding performance data from 145 team leaders and 145 managers).	Hypothesis testing: Association between scale scores and (i) effectiveness and efficiency of team performance (subjective ratings by team members, team leaders, external managers), (ii) work satisfaction, (iii) individual knowledge and skills. Mixed support for hypotheses.<br><br>Structure: A single theoretical construct (teamwork quality) was predicted and empirically supported (principle components analysis). The dimensionality of the construct was not assessed.	Internal consistency: Cronbach's alpha for the six scales ranged from 0.72 to 0.94.<br><br>Stability over time: not assessed<br><br>Inter-rater reliability: coefficients of inter-rater reliability indicated within-group agreement, supporting the reliability of the measure at team level.	Respondents completed the instrument in 45 minute interviews. Completion time was not reported, however other questions appeared to be a minor component of the interview.
Decision making process and satisfaction scales<br><br>Janssen 1999 [68]	Items were derived from existing scales and adapted to measure conflict behaviour during team decision making.<br><br>Content validity: no independent assessments reported.<br><br>Administered to managers participating in a development program, who were asked to respond in relation to their experience on a managerial team over the previous 12 months (RR 102/115, 89%).	Hypothesis testing: Association between process scale scores and (i) predictors of conflict behaviour (interdependence, task and person conflict), and (ii) outcomes (decision quality, affective response to the team, perceived team effectiveness). Most hypotheses supported.<br><br>Structure: Two theoretical dimensions were predicted (process scales), both were empirically supported (principal components analysis).	Internal consistency: Cronbach's alpha for the two process scales was 0.83 and 0.80, and the satisfaction scale 0.80.<br><br>Stability over time: not assessed<br><br>Inter-rater reliability: not assessed (single respondent per team)	No other assessments reported.

Instrument (name, index, references for main reports)	Development & content validity (item generation; content validity assessments)	Construct validity	Reliability	Other assessments (eg feasibility, acceptability, interpretability)
Extended Intragroup Conflict Scale<br><br>Jehn 2008 [53, 69]	Items were derived from earlier versions of the instrument [53, 70] and other sources.<br><br>Content validity: no independent assessments reported.<br><br>Administered to undergraduate student performing a simulated management team exercise (RR not reported. 223 members from 53 groups).	Hypothesis testing: Association between scale scores and (i) emergent states (trust, respect, cohesion; rated by team members and observers), (ii) outcomes (group performance, viability). Moderating effect of conflict dimensions (resolution efficacy, norms, emotions, importance) on the relationship between conflict type (relationship, task, process), emergent states and outcomes. Mixed support for hypotheses.<br><br>Structure: Three types of conflict and four dimensions of each conflict type were predicted by theory and empirically supported (principal components analysis).	Internal consistency: Cronbach's alpha for the three conflict type scales ranged from 0.83 to 0.90, and for the four conflict dimension scales 0.55 to 0.95.<br><br>Stability over time: not assessed<br><br>Inter-rater reliability: intraclass correlation coefficients for all scales indicated adequate within group agreement and between group variation.	No other assessments reported.<br><br>Selected items from an earlier version of scale used in healthcare teams (see characteristics of included instruments), but no examples of use of this version identified in health care.
Team empowerment<br><br>Kirkman 1999 [71]	Items were adapted from existing scales [72-73] for team-level measurement.<br><br>Content validity: no independent assessments reported.<br><br>Administered to manufacturing teams (RR 98/112 teams, 88%; average team size of 12.75).	Hypothesis testing: Association between scale scores and (i) antecedents of empowerment (external leader behaviour; responsibility for and structuring of work; human resource policies), (ii) team performance (supervisor rated), (iii) work attitudes (team member rated) [71]. All hypotheses supported.<br><br>Structure: Four theoretical dimensions were predicted and were empirically supported (exploratory factor analysis). However, the scales were highly correlated, suggesting the scales cannot be discriminated from each other.	Internal consistency: Cronbach's alpha for the four scales ranged from 0.82 to 0.94.<br><br>Stability over time: not assessed<br><br>Inter-rater reliability: not applicable. Team consensus methods were used to rate team empowerment (see [74] for comparison of aggregation versus consensus approach).	No other assessments reported.<br><br>This instrument has been used in a number of other studies (for example, [75-76]). One study used the model as the basis for a measure of empowerment in health care [77], but items are not reported in full so overlap can't be assessed.
Group reaction questionnaire (GRQ)<br><br>Kuhn 2000 [78]	Methods of item generation not reported.<br><br>Content validity: no independent assessments reported.<br><br>Administered to QI teams in a government agency and a large corporation (RR 5/11 teams, 45% provided data for analysis). Conflict management was measured through analysis of data from multiple observations of team meetings over 8-20 months.	Hypothesis testing: Association between decision making effectiveness and (i) group conflict management styles (observer rated), and (ii) task complexity. No results reported for GRQ alone (see comment under 'other assessments').<br><br>Structure: not assessed	Internal consistency: Cronbach's alpha for scale was 0.79.<br><br>Stability over time: not assessed<br><br>Inter-rater reliability: not assessed	No other assessments reported.<br><br>Scores on the GRQ (team, facilitator, observer rated) provided 3 of 7 measures used to create a composite measure of decision making effectiveness (rated as 'good' or 'other' based on qualitative assessment of the 7 measures). There are no data on which to assess the properties of the GRQ.

**Instrument** (name, index, references for main reports)	**Development & content validity** (item generation; content validity assessments)	**Construct validity**	**Reliability**	**Other assessments** (eg feasibility, acceptability, interpretability)
Team learning behaviours instrument<br><br>Savelsbergh 2009 [30]	Items were derived from existing validated scales [79] [36, 80-84] to operationalise the construct as defined from a review of theoretical literature.<br><br>Content validity: Content experts in organisational behaviour and project teams reviewed items for relevance to the construct.<br><br>Administered to teams in the banking sector (RR 211/274, 77% of members and leaders from 19/38 teams. 19 teams excluded because team level RR was < 50%).	Hypothesis testing: Association between scales scores and perceived team effectiveness (composite score based on rating by team members and team leaders). Correlation between scale scores was used to assess convergent and discriminant validity. Most hypotheses supported.<br><br>Structure: Five theoretical dimensions were predicted. Eight were empirically supported (factors identified by principal component analysis and subsequent confirmatory factor analysis).	Internal consistency: Cronbach's alpha for the eight scales ranged from 0.71 to 0.87.<br><br>Stability over time: not assessed<br><br>Inter-rater reliability: not assessed	No other assessments reported.
Team reflexivity scales<br><br>Schippers 2007 [85]	New items were written to operationalised the construct and combined with items from an existing scale [86].<br><br>Content validity: some items were revised or removed following i) review by content experts, and ii) pilot testing with a team to identify ambiguous, irrelevant or difficult to understand items.<br><br>Administered to teams from public and private sector organisations (RR 453 members from 59/60, teams), then to teams in schools (RR 228/235 members from 59/60 teams) [85]. Subsequent study with public and private sector organisations (RR 238/250, 95% from 32 teams) [87].	Hypothesis testing: Association between scale scores and (i) measures of related constructs (feedback seeking, personality, individual learning style) [85], (ii) transformational leadership, (iii) shared vision, and (iv) perceived team performance (supervisor rated) [87]. Hypotheses supported.<br><br>Structure: Three theoretical dimensions were predicted, two of which were empirically supported (exploratory and confirmatory factor analysis on separate samples). Adaptation items were not included in the analysis.	Internal consistency: Cronbach's alpha for the two scales was 0.90 and 0.76.<br><br>Stability over time: not assessed<br><br>Inter-rater reliability: r_{wg} and intraclass correlation coefficients for both scales indicated adequate within group agreement and between group variation.	No other assessments reported.<br><br>Selected items were used in one study in health care [88], but items were not reported so can't assess whether this study provides additional evidence about the scales measurement properties.
Constructive controversy scale<br><br>Tjosvold 1986 [89]	Methods of item generation not reported<br><br>Content validity: no independent assessments reported.<br><br>Administered to managers with involvement in team-based decision making (RR not reported; 52 managers).	Hypothesis testing: Association between scale score and (i) decision making style (autocratic, consultative, group), (ii) perceived effectiveness, quality, originality and acceptance of the decision, (iii) satisfaction with the decision making process. Most hypotheses supported.<br><br>Structure: Items formed a single scale (identified by exploratory factor analysis).	Internal consistency: Cronbach's alpha for the scale was 0.88.<br><br>Stability over time: not assessed<br><br>Inter-rater reliability: not assessed (single respondent only)	No other assessments reported.
Beliefs about interpersonal context for team learning<br><br>Van den Bossche 2006a [79]	Items were derived from existing validated scales [36, 90-93]. Some items were deleted because they discriminated poorly between the theorised dimensions (based on factor analysis).<br><br>Content validity: content experts reviewed the items and the scales were pre-tested with teams to confirm appropriateness of wording.<br><br>Administered to students participating in team projects (RR 75/90, 75% of team contributed data suitable for analysis).	Hypothesis testing: Association between scale scores and (i) team learning behaviours, (ii) mutually shared cognitions, (iii) perceived team effectiveness (team member rating of performance, viability, global rating of learning). Most hypotheses supported.<br><br>Structure: Five theoretical dimensions were predicted; all were empirically supported (principal component analysis).	Internal consistency: Cronbach's alpha for the five scales ranged from 0.50 to 0.89.<br><br>Stability over time: not assessed<br><br>Inter-rater reliability: r_{wg} indicated acceptable within group agreement for each of the scales.	No other assessments reported.

**Instrument** (name, index, references for main reports)	**Development & content validity** (item generation; content validity assessments)	**Construct validity**	**Reliability**	**Other assessments** (eg feasibility, acceptability, interpretability)
Team learning behaviours questionnaire<br><br>Van den Bossche 2006b [79, 94]	Items were derived from existing validated scales [36, 95-96].<br><br>Content validity: content experts reviewed the items and the scales were pre-tested with teams to confirm appropriateness of wording.<br><br>Administered to students participating in team projects (RR 75/90, 75% of team contributed data suitable for analysis).	Hypothesis testing: Association between scale scores and (i) interpersonal context, (ii) mutually shared cognitions, (iii) perceived team effectiveness (team member rating of performance, viability, global rating of learning). Most hypotheses supported.<br><br>Structure: Items formed a single scale (identified by principal component analysis).	Internal consistency: Cronbach's alpha for the scales was 0.88.<br><br>Stability over time: not assessed<br><br>Inter-rater reliability: r_{wg} indicated acceptable within group agreement for the scale.	No other assessments reported.
Team Diagnostic Survey<br><br>Wageman 2005 [97]	Items were derived from existing validated scales previously developed by the authors.<br><br>Content validity: some items were revised following the first administration to teams to improve unclear or confusing wording. No other assessments of content reported.<br><br>Initial administration to teams from "diverse" organisations (1272 members from 181 teams, RR not reported). Subsequent administration to teams for initial testing (2472 members from 321 teams, RR not reported).	Hypothesis testing: Association between scale scores (discriminant and convergent validity). Difference between groups expected to differ on the scale scores. Most hypotheses supported<br><br>Structure: not assessed	Internal consistency: Cronbach's alpha for the scales ranged from 0.31 to 0.92 (individual level data) and 0.65 to 0.984 (team level data).<br><br>Stability over time: not assessed<br><br>Inter-rater reliability: intraclass correlation coefficients indicated acceptable within group agreement for most scales.	Completion time was reported as 20 minutes.<br><br>No other assessments reported.

References

1. Anderson N, West M: **Measuring climate for work group innovation: Development and validation of the Team Climate Inventory.** *J Organ Behav* 1998, **19:**235-258.
2. Kivimaki M, Kivimaki M, Elovainio M: **A short version of the Team Climate Inventory: Development and psychometric properties.** *J Occup Organ Psych* 1999, **72:**241-246.
3. Bower P, Campbell S, Bojke C, Sibbald B: **Team structure, team climate and the quality of care in primary care: an observational study.** *Qual Saf Health Care* 2003, **12:**273-279.
4. Bosch M, Dijkstra R, Wensing M, van der Weijden T, Grol R: **Organizational culture, team climate and diabetes care in small office-based practices.** *BMC Health Serv Res* 2008, **8:**180.
5. Hann M, Bower P, Campbell S, Marshall M, Reeves D: **The association between culture, climate and quality of care in primary health care teams.** *Fam Pract* 2007, **24:**323-329.
6. Goh TT, Eccles MP, Steen N: **Factors predicting team climate, and its relationship with quality of care in general practice.** *BMC Health Serv Res* 2009, **9:**138.
7. Campbell SM, Hann M, Hacker J, Burns C, Oliver D, Thapar A, Mead N, Safran DG, Roland MO: **Identifying predictors of high quality care in English general practice: observational study.** *BMJ* 2001, **323:**784-.
8. Proudfoot J, Jayasinghe UW, Holton C, Grimm J, Bubner T, Amoroso C, Beilby J, Harris MF: **Team climate for innovation: what difference does it make in general practice?** *Int J Qual Health Care* 2007, **19:**164-169.
9. Poulton BC, West MA: **The determinants of effectivenss in primary health care teams.** *J Interprof Care* 1999, **13:**7-18.
10. Fay D, Borrill C, Amir Z, Haward R, West MA: **Getting the most out of multidisciplinary teams: A multi-sample study of team innovation in health care.** *J Occup Organ Psychol* 2006, **79:**553-567.
11. Elovainio M, Kivimaki M, Eccles M, Sinervo T: **Team climate and procedural justice as predictors of occupational strain.** *J Appl Soc Psychol* 2002, **32:**359-374.
12. Harris MF, Proudfoot JG, Jayasinghe UW, Holton CH, Powell Davies GP, Amoroso CL, Bubner TK, Beilby JJ: **Job satisfaction of staff and the team environment in Australian general practice.** *Med J Aust* 2007, **186:**570-573.

13. Eccles MP, Hrisos S, Francis JJ, Stamp E, Johnston M, Hawthorne G, Steen N, Grimshaw JM, Elovainio M, Presseau J, Hunter M: **Instrument development, data collection, and characteristics of practices, staff, and measures in the Improving Quality of Care in Diabetes (iQuaD) Study.** *Implement Sci* 2011, **6**.

14. Loo R, Loewen P: **A Confirmatory Factor-Analytic and Psychometric Examination of the Team Climate Inventory: Full and Short Versions.** *Small Gr Res* 2002, **33:**254-265.

15. Hearnshaw H, Reddish S, Carlyle D, Baker R, Robertson N: **Introducing a quality improvement programme to primary healthcare teams.** *Qual Health Care* 1998, **7:**200-208.

16. Furnham A, Gunter B: *Corporate culture: auditing a company's personality.* London: Routledge; 1993.

17. Lukas CV, Mohr DC, Meterko M: **Team effectiveness and organizational context in the implementation of a clinical innovation.** *Qual Manag Health Care* 2009, **18:**25-39.

18. Lemieux-Charles L, Murray M, Baker G, Barnsley J, Tasa K, Ibrahim S: **The effects of quality improvement practices on team effectiveness: a mediational model.** *J Organ Behav* 2002, **23:**533-553.

19. Shortell SM, Marsteller JA, Lin M, Pearson ML, Wu SY, Mendel P, Cretin S, Rosen M: **The role of perceived team effectiveness in improving chronic illness care.** *Med Care* 2004, **42:**1040-1048.

20. VanDeusen Lukas C, Meterko MM, Mohr D, Seibert MN, Parlier R, Levesque O, Petzel RA: **Implementation of a clinical innovation: The case of advanced clinic access in the Department of Veterans Affairs.** *J Ambulatory Care Manage* 2008, **31:**94-108.

21. Cretin S, Shortell SM, Keeler EB: **An evaluation of collaborative interventions to improve chronic illness care. Framework and study design.** *Eval Rev* 2004, **28:**28-51.

22. Shortell SM, Rousseau DM, Gillies RR, Devers KJ, Simons TL: **Organizational assessment in intensive care units (ICUs): construct development, reliability, and validity of the ICU nurse-physician questionnaire.** *Med Care* 1991, **29:**709-726.

23. Hall CB, Tennen H, Wakefield DB, Brazil K, Cloutier MM: **Organizational assessment in paediatric primary care: development and initial validation of the primary care organizational questionnaire.** *Health Serv Manage Res* 2006, **19:**207-214.

24. Brazil K, Wakefield DB, Cloutier MM, Tennen H, Hall CB: **Organizational culture predicts job satisfaction and perceived clinical effectiveness in pediatric primary care practices.** *Health Care Manage Rev* 2010, **35:**365-371.

25. Cloutier MM, Wakefield DB, Tsimikas J, Hall CB, Tennen H, Brazil K: **Organizational Attributes of Practices Successful at a Disease Management Program.** *J Pediatr* 2009, **154:**290-295.

26. Caldwell DF, O'Reilly CA, III: **The determinants of team-based innovation in organizations. The role of social influence.** *Small Gr Res* 2003, **34:**497-517.

27. Strating MMH, Nieboer AP: **Norms for creativity and implementation in healthcare teams: testing the group innovation inventory.** *Int J Qual Health Care* 2010, **22:**275-282.
28. Campion MA, Medsker GJ, Higgs AC: **Relations between work group characteristics and effectiveness: Implications for designing effective work groups.** *Pers Psychol* 1993, **46:**823-850.
29. Campion MA, Papper EM, Medsker GJ: **Relations between work team characteristics and effectiveness: A replication and extension.** *Pers Psychol* 1996, **49:**429-452.
30. Savelsbergh CM, van der Heijden BI, Poell RF: **The development and empirical validation of a multidimensional measurement instrument for team learning behaviors.** *Small Gr Res* 2009, **40:**578-607.
31. Shaw JD, Duffy MK, Stark EM: **Interdependence and preference for group work: Main and congruence effects on the satisfaction and performance of group members.** *J Manage* 2000, **26:**259-279.
32. Stewart GL, Barrick MR: **Team structure and performance: Assessing the mediating role of intrateam process and the moderating role of task type.** *Acad Manage J* 2000, **43:**135-148.
33. Costa AC, Anderson N: **Measuring trust in teams: Development and validation of a multifaceted measure of formative and reflective indicators of team trust.** *Eur J Work Org Pscychol* 2011, **20:**119-154.
34. Dobson RT, Stevenson K, Busch A, Scott DJ, Henry C, Wall PA: **A quality improvement activity to promote interprofessional collaboration among health professions students.** *Am J Pharm Educ* 2009, **73:**64.
35. Clark PG: **Learning on interdisciplinary gerontological teams: intructional concepts and methods.** *Educ Gerontol* 1994, **20:**349-364.
36. Edmondson A: **Psychological safety and learning behavior in work teams.** *Adm Sci Q* 1999, **44:**350-383.
37. Nembhard IM, Edmondson AC: **Making it safe: the effects of leader inclusiveness and professional status on psychological safety and improvement efforts in health care teams.** *J Organ Behav* 2006, **27:**941-966.
38. Wilkens R, London M: **Relationships between climate, process, and performance in continuous quality improvement groups.** *J Vocat Behav* 2006, **69:**510-523.
39. Hackman JR: **The design of work teams.** In *Handbook of organizational behavior.* Edited by Lorsch JW. Englewood Cliffs, NJ: Prentice-Hall; 1987: 315-342
40. Lewis K: **Measuring transactive memory systems in the field: Scale development and validation.** *J Appl Psychol* 2003, **88:**587-604.

41. Michinov E: **Validation of a French version of the Transactive Memory Scale and its adaptation to an academic context.** *European Review of Applied Psychology/Revue Europeenne de Psychologie Appliquee* 2007, **57:**59-68.
42. Michinov E, Olivier-Chiron E, Rusch E, Chiron B: **Influence of transactive memory on perceived performance, job satisfaction and identification in anaesthesia teams.** *Br J Anaesth* 2008, **100:**327-332.
43. Lichtenstein R, Alexander J, Jinnett A, Ullman E: **Embedded intergroup relations in interdisciplinary teams: Effects on perceptions of level of team integration.** *J Appl Behav Sci* 1997, **33:**413-434.
44. Alexander JA, Lichtenstein R, Jinnett K, Wells R, Zazzali J, Liu D: **Cross-functional team processes and patient functional improvement.** *Health Serv Res* 2005, **40:**1335-1355.
45. Lubomski LH, Marsteller JA, Hsu YJ, Goeschel CA, Holzmueller CG, Pronovost PJ: **The team checkup tool: evaluating QI team activities and giving feedback to senior leaders.** *Jt Comm J Qual Patient Saf* 2008, **34:**619-623, 561.
46. Chan KS, Hsu YJ, Lubomski LH, Marsteller JA: **Validity and usefulness of members reports of implementation progress in a quality improvement initiative: Findings from the Team Check-up Tool (TCT).** *Implement Sci* 2011, **6**.
47. Mills PD, Weeks WB: **Characteristics of successful quality improvement teams: lessons from five collaborative projects in the VHA.** *Jt Comm J Qual Saf* 2004, **30:**152-162.
48. Weeks WB, Mills PD, Dittus RS, Aron DC, Batalden PB: **Using an improvement model to reduce adverse drug events in VA facilities.** *Jt Comm J Qual Improv* 2001, **27:**243-254.
49. Millward LJ, Jeffries N: **The team survey: a tool for health care team development.** *J Adv Nurs* 2001, **35:**276-287.
50. Schouten L, Grol R, Hulscher M: **Factors influencing success in quality improvement collaboratives: development and psychometric testing of an instrument.** *Implement Sci* 2010, **5:**84.
51. Thompson BM, Levine RE, Kennedy F, Naik AD, Foldes CA, Coverdale JH, Kelly PA, Parmelee D, Richards BF, Haidet P: **Evaluating the quality of learning-team processes in medical education: development and validation of a new measure.** *Acad Med* 2009, **84:**S124-127.
52. Bunderson JS, Sutcliffe KM: **Management team learning orientation and business unit performance.** *J Appl Psychol* 2003, **88:**552-560.
53. Jehn KA: **A multimethod examination of the benefits and detriments of intragroup conflict.** *Adm Sci Q* 1995, **40:**256-282.
54. Morrison EW: **Longitudinal study of the effects of information seeking on newcomer socialization.** *J Appl Psychol* 1993, **78:**173-183.

55. VandeWalle D: **Development and validation of a work domain goal orientation instrument.** *Educ Psychol Meas* 1997, **57:**995-1015.
56. VandeWalle D, Ganesan S, Challagalla GN, Brown SP: **An integrated model of feedback-seeking behavior: Disposition, context, and cognition.** *J Appl Psychol* 2000, **85:**996-1003.
57. Arnold JA, Arad S, Rhoades JA, Drasgow F: **The Empowering Leadership Questionnaire: the construction and validation of a new scale for measuring leader behaviors.** *J Organ Behav* 2000, **21:**249-269.
58. Bunderson JS, Sutcliffe KM: **Why some teams emphasize learning more than others: Evidence from business unit management teams.** In *Research on managing groups and teams. Volume* 4. Edited by Sondak H, Mannix EA, Neale MA. Oxford, England: Elsevier Science; 2002: 49–84
59. Bunderson J, Boumgarden P: **Structure and learning in self-managed teams: Why "bureaucratic" teams can be better learners.** *Organ Sci* 2010, **21:**609-624.
60. Coyle-Shapiro JAM, Morrow PC: **The role of individual differences in employee adoption of TQM orientation.** *J Vocat Behav* 2003, **62:**320-340.
61. Cook J, Wall T: **New work attitude measures of trust, organizational commitment and personal need non-fulfilment. Mar 1980 1980.** *J Occup Psychol* 1980, **Vol.53:**pp.
62. Driskell JE, Salas E, Hughes S: **Collective orientation and team performance: development of an individual differences measure.** *Hum Factors* 2010, **52:**316-328.
63. Hackman J: *Groups that work (and those that don't): creating conditions for effective teamwork.* San Francisco: Jossey Bass; 1990.
64. Hiller NJ, Day DV, Vance RJ: **Collective enactment of leadership roles and team effectiveness: A field study.** *Leadership Quart* 2006, **17:**387-397.
65. Yukl G, Lepsinger R: **Preliminary report on validation of the Managerial Practices Survey.** In *Measures of leadership.* Edited by Clark KE, Clark MB. West Orange, NJ: Leadership Library of America; 1990: 223−237
66. Hoegl M, Gemuenden HG: **Teamwork quality and the success of innovative projects: A theoretical concept and empirical evidence.** *Organ Sci* 2001, **12:**435-449.
67. Pinto MB, Pinto JK, Prescott JE: **Antecedents and consequences of project team cross-functional cooperation.** *Manage Sci* 1993, **39:**1281-1297.
68. Janssen O, Van de Vliert E, Veenstra C: **How task and person conflict shape the role of positive interdependence in management teams.** *J Manage* 1999, **25:**117-142.

69. Jehn KA, Greer L, Levine S, Szulanski G: **The effects of conflict types, dimensions, and emergent states on group outcomes.** *Group Decis Negot* 2008, **17:**465–495.

70. Pearson AW, Ensley MD, Amason AC: **An assessment and refinement of Jehn's intragroup conflict scale.** *Int J Confl Manage* 2002, **13:**110-126.

71. Kirkman BL, Rosen B: **Beyond self-management: antecedents and consequences of team empowerment.** *Acad Manage J* 1999, **42:**58-74.

72. Guzzo RA, Yost PR, Campbell RJ, Shea GP: **Potency in groups: Articulating a construct.** *Br J Soc Psychol* 1993, **32:**87-106.

73. Thomas KW, Tymon WG, Jr.: *Empowerment inventory.* Tuxedo, NY: Xicom; 1993.

74. Kirkman BL, Tesluk PE, Rosen B: **Assessing the incremental validity of team consensus ratings over aggregation of individual-level data in predicting team effectiveness.** *Pers Psychol* 2001, **54:**645-667.

75. Chen G, Kirkman BL, Kanfer R, Allen D, Rosen B: **A multilevel study of leadership, empowerment, and performance in teams.** *J Appl Psychol* 2007, **92:**331-346.

76. Kirkman BL, Rosen B, Tesluk PE, Gibson CB: **The impact of team empowerment on virtual team performance: the moderating role of face-to-face interaction.** *Acad Manage J* 2004, **47:**175-192.

77. Proenca EJ: **Team dynamics and team empowerment in health care organizations.** *Health Care Manage Rev* 2007, **32:**370-378.

78. Kuhn T, Poole MS: **Do conflict management styles affect group decision making? Evidence from a longitudinal field study.** *Hum Commun Res* 2000, **26:**558-590.

79. van den Bossche P, Gijselaers WH, Segers M, Kirschner PA: **Social and Cognitive Factors Driving Teamwork in Collaborative Learning Environments: Team Learning Beliefs and Behaviors.** *Small Gr Res* 2006, **37:**490-521.

80. Schippers MC, Den Hartog DN, Koopman PL, Wienk JA: **Diversity and team outcomes: the moderating effects of outcome interdependence and group longevity and the mediating effect of reflexivity.** *J Organ Behav* 2003, **24:**779-802.

81. Watkins KE, Marsick VJ: *Dimensions of the learning organization questionnaire.* Warwick: Partners for the Learning Organization; 1998.

82. van Dyck C, Frese M, Baer M, Sonnentag S: **Organizational Error Management Culture and Its Impact on Performance: A Two-Study Replication.** *J Appl Psychol* 2005, **90:**1228-1240.

83. Van Offenbeek M, Koopman PL: **Interaction and decision making in project teams.** In *Handbook of work group psychology.* Edited by West MA. Chichester, UK: John Wiley; 1996: 159-187

84. Van Woerkom M: **Critical reflection at work: Bridging individual and organizational learning. Unpublished doctoral dissertation.** University of Twente, Enschede, the Netherlands, 2003.

85. Schippers MC, Den Hartog DN, Koopman PL: **Reflexivity in teams: A measure and correlates.** *Appl Psychol Int Rev* 2007, **56:**189-211.

86. Swift TA, West MA: **Reflexivity and group processes: Research and practice**. The ESRC Centre for Organization and Innovation Sheffield 1998.

87. Schippers MC, Den Hartog DN, Koopman PL, van Knippenberg D: **The role of transformational leadership in enhancing team reflexivity.** *Hum Relat* 2008, **61:**1593-1616.

88. Adler-Milstein J, Neal K, Howell MD: **Residents' and nurses' perceptions of team function in the medical intensive care unit.** *J Crit Care* 2011, **26:**104.e107-115.

89. Tjosvold D, Wedley WC, Field RH: **Constructive controversy, the Vroom-Yetton model, and managerial decision-making.** *J Occup Behav* 1986, **7:**125-138.

90. Carless SA, De Paola C: **The measurement of cohesion in work teams.** *Small Gr Res* 2000, **31:**71-88.

91. van der Vegt G, Emans B, van de Vliert E: **Motivating effects of task and outcome interdependence in work teams.** *Group Organ Manage* 1998, **23:**124-143.

92. Sargent LD, Sue-Chan C: **Does Diversity Affect Group Efficacy? The Intervening Role of Cohesion and Task Interdependence.** *Small Gr Res* 2001, **32:**426-450.

93. Gibson CB, Randel AE, Earley PC: **Understanding group efficacy: An empirical test of multiple assessment methods.** *Group Organ Manage* 2000, **25:**67-97.

94. van den Bossche P, Gijselaers W, Segers M, Woltjer G, Kirschner P: **Team learning: Building shared mental models.** *Instr Sci* 2011, **39:**283-301.

95. Visschers-Pleijers AJSF, Dolmans DHJM, Wolfhagen IHAP, Van der Vleuten CPM: **Development and validation of a questionnaire to identify interactions that promote deep learning in PBL** In *Paper presented at the 10th European conference of research on learning and instruction; Padova, Italy*. 2003

96. van Offenbeek M: **Processes and outcomes of team learning.** *Eur J Work Org Pscychol* 2001, **10:**303-317.

97. Wageman R, Hackman J, Lehman E: **Team Diagnostic Survey: Development of an Instrument.** *J Appl Behav Sci* 2005, **41:**373-398.
